# Utilization of nicking properties of CRISPR-Cas12a effector for genome editing

**DOI:** 10.1038/s41598-024-53648-2

**Published:** 2024-02-09

**Authors:** Chan Hyoung Kim, Wi-jae Lee, Yeounsun Oh, Youngjeon Lee, Hyomin K. Lee, Jung Bae Seong, Kyung-Seob Lim, Sang Je Park, Jae-Won Huh, Young-Hyun Kim, Kyoung Mi Kim, Junho K. Hur, Seung Hwan Lee

**Affiliations:** 1 Department of Life Science, Chung-Ang University, Seoul, 06974 Republic of Korea; 2 National Primate Research Center (NPRC), Korea Research Institute of Bioscience and Biotechnology (KRIBB), Cheongju, 28116 Republic of Korea; 3 Department of Biological Sciences, Chungnam National University, Daejeon, Republic of Korea; 4 Department of Functional Genomics, KRIBB School of Bioscience, Korea University of Science and Technology (UST), Daejeon, Republic of Korea; 5 Department of Medicine, Major in Medical Genetics, Graduate School, Hanyang University, Seoul, 04763 Republic of Korea; 6 Futuristic Animal Resource and Research Center (FARRC), Korea Research Institute of Bioscience and Biotechnology (KRIBB), Cheongju, Republic of Korea; 7 Department of Genetics, College of Medicine, Hanyang University, Seoul, 04763 Republic of Korea; 8 Graduate School of Biomedical Science and Engineering, Hanyang University, Seoul, 04763 Republic of Korea

**Keywords:** Biotechnology, Molecular biology

## Abstract

The CRISPR-Cas nickase system for genome editing has attracted considerable attention owing to its safety, efficiency, and versatility. Although alternative effectors to Cas9 have the potential to expand the scope of genome editing, their application has not been optimized. Herein, we used an enhanced CRISPR-Cas12a nickase system to induce mutations by targeting genes in a human-derived cell line. The optimized CRISPR-Cas12a nickase system effectively introduced mutations into target genes under a specific directionality and distance between nickases. In particular, the single-mode Cas12a nickase system can induce the target-specific mutations with less DNA double-strand breaks. By inducing mutations in the Thymine-rich target genes in single- or dual-mode, Cas12a nickase compensates the limitations of Cas9 nickase and is expected to contribute to the development of future genome editing technologies.

The CRISPR-Cas system is known as a bacterial immune system and is receiving much attention for future in vivo applications because it can induce target-specific gene mutations^1,2^. Among these CRISPR-Cas systems, the Cas9 effector, whose mechanism was first identified^2^, has been optimized and broadly studied for gene editing^1^. Further application to therapeutics, various Cas9-based methods have been devised to accurately induce genome editing in vivo, including the human body^3,4^. Among these methods, the use of nickase produced by engineering the active site of Cas9 is currently gaining importance in various fields^5^. Particularly, the use of mono- or dual-type nickases for generation of random mutations^5,6^, and for single-base resolution correction such as base editing^7,8^ or prime editing^9^ is very important for development of genome editing technology and has a large ripple effect on the bio-medical field. Since the CRISPR-Cas9 system is well conserved in various species, Cas9 orthologs with a similar nicking mechanism have been applied in many studies to induce gene editing in vivo^10^. However, for other types of effectors, especially the Cas12 type, the mechanism of inducing a nick on the target DNA is not precisely known yet; and many studies are currently being performed to reveal the nicking property of the Cas12 system^11–14^.

Herein, CRISPR-Cas12a nickase was used to induce target-specific gene editing in human-derived cell lines. In this study, using a plasmid cleavage assay, we showed that the en-AsCas12a (R1226A) system can mainly form nicks on target DNA sequences. We used a nickase version with enhanced PAM recognition in the target sequence to improve the efficiency of DNA mutagenesis in human-derived cell line by effectively operate the CRISPR-Cas12a nickase system. In addition, the distance and directionality between nickases were optimized for better editing efficiency. Target specific indels (0.37 ± 0.22–48.28 ± 6.18%) were induced by using dual en-AsCas12a (R1226A) nickase for various gene sites, and it was shown that unintended off-target editing can be decreased for specific endogenous sites compared to the wt-Cas12a system. This study demonstrates that we have developed a Cas12a (R1226A)-based gene editing method that can act safely with reduced off-target activity in human-derived cell line. It also complements the shortcomings of wild-type Cas9 or Cas12a modules that directly induce double-strand breaks (DSBs), and has the potential to drive precise genome editing in a variety of organisms in the future.

## Results

### Production of the en-AsCas12a (R1226A) variant and identification of the nicking property

**Figure 1:**
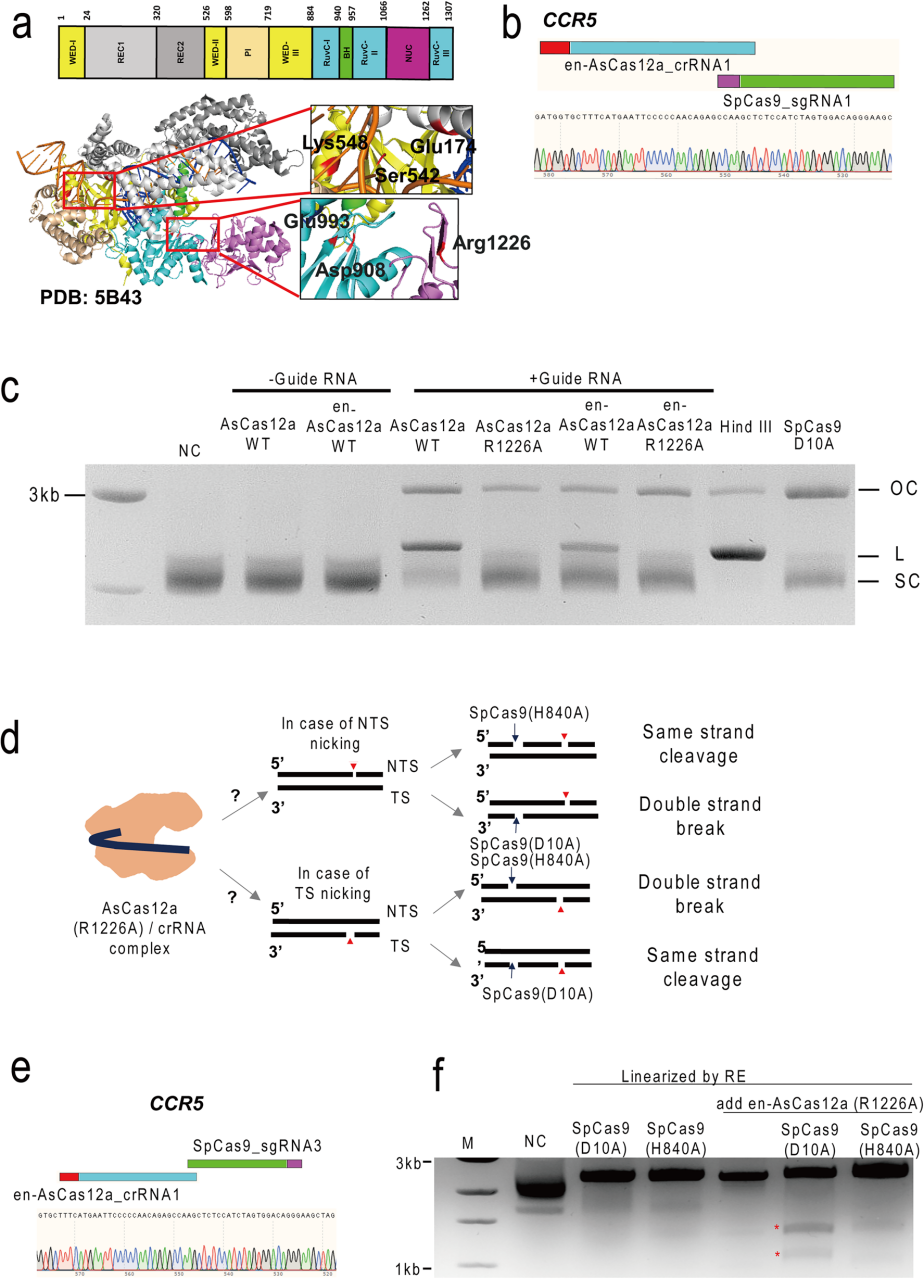
Confirmation of the nicking activity of en-AsCas12a (R1226A) module. (**a**) Structure of the AsCas12a-crRNA-target DNA complex (PDB: 5B43). Left inset: substituted amino acid residues in the AsCas12a protein around the PAM sequence (TTTN) (E174, S542, K548). Right inset: Active residues in the nuclease domain required for target DNA cleavage. (**b**, **c**) Sanger sequencing data of the plasmid containing the target nucleotide sequence (*CCR5*) (**b**). PAM and target sequences are shown in cyon (SpCas9) and yellow (AsCas12a) and dark blue (SpCas9) and gray (AsCas12a), respectively. Plasmid cleavage assay using recombinant proteins (en-AsCas12a, en-AsCas12a (R1226A), SpCas9 (D10A)) (**c**). NC: negative control, HindIII: Treated by restriction enzyme (HindIII), OC: open circular form, L: linear form, SC: super-coiled form. (–) guide RNA: only en-AsCas12a or en-AsCas12a (R1226A) protein treated, ( +) guide RNA: en-AsCas12a or en-AsCas12a (R1226A) protein and target-specific crRNA were co-treated. (**d**–**f**) Schematics depicting a method for identification of the cleaved strand formed by en-AsCas12a (R1226A) on target DNA (**d**). NTS and TS indicates non-target strand and target strand in target DNA, respectively. The red arrowhead indicates the nick formed by en-Cas12a (R1226A). Blue arrows indicate nicks formed by SpCas9 (D10A) or SpCas9 (H840A), respectively. Sanger sequencing data of the plasmid containing the target nucleotide sequence (*CCR5*) (**e**). *In-vitro* DNA cleavage assay for cleavage strand identification using en-AsCas12a (R1226A), SpCas9 (D10A), and SpCas9 (H840A) (**f**). Red asterisk indicates a cleaved DNA fragments. RE: restriction enzyme, NC: negative control, SpCas9 (D10A) or SpCas9 (H840A): SpCas9 nickases, en-AsCas12a (R1226A): en-AsCas12a nickase.

The CRISPR-Cas12a system consists of a single CRISPR RNA (crRNA) and Cas12a endonuclease that induces target DNA recognition^15,16^. The Cas12a effector recognizes a T-rich protospacer adjacent motif (PAM) among target sequences (Fig. 1a, left inset), crRNA and target DNA hybridization induces an R-loop, and finally the active site of the RuvC domain (Fig. 1a, right inset) induces DNA cleavage^17^. The recently reported enhanced AsCas12a (en-AsCas12a) system shows that engineered amino acids near the PAM sequence (TTTN) dramatically enhances target DNA recognition, thereby increasing genome editing efficiency^18^. We optimized a nickase effector based on the en-AsCas12a (E174R, S542R, K548R) system, hereafter en-AsCas12a (R1226A), to effectively induce target-specific gene mutations with single- or dual mode of DNA targeting. To confirm the nicking property at the *in-vitro* level, a cleavage assay was performed by targeting the plasmid containing the target (*EMX1, CCR5*) nucleotide sequence (Fig. 1b,c, Supplementary Supplementary Fig. 1a, Tables 1, 2). As previously reported^11,12^, the wild-type (WT) en-AsCas12a effector showed a minor nicking property while inducing DNA double strand break (Fig. 1c, Supplementary Fig. 1b). On the other hand, the en-AsCas12a (R1226A) form showed a typical activity as a DNA nickase by mainly inducing the open circular form of the plasmid (Fig. 1c). After confirming the nicking property of the en-Cas12a (R1226A) variant, we tried to determine whether en-Cas12a (R1226A) cleaves the target-strand or non-target strand on the target DNA. To this end, we co-treated the Cas12a (R1226A) variant with SpCas9 (D10A) or SpCas9 (H840A) nickases, whose cleavage points have already been identified, and the cleaved strand of target DNA by Cas12a (R1226A) variant was identified by the formation of double strand breaks (Fig. 1d). As a result, we found that when Cas12a (R1226A) variant, SpCas9 (D10A), and SpCas9 (H840A) nickases were simultaneously treated (Fig. 1e), double strand breaks were mainly formed in case of the Cas12a (R1226A) variant and SpCas9 (D10A) nickase were co-treated (Fig. 1f). Furthermore, we analyzed the product after cleavage using the Cas12a (R1226A) variant and compared it to Cas9 (D10A or H840A) and found that a cleavage was formed at a distance of 15–18 bp from the PAM sequence (TTTN), depending on the target sequence site (Supplementary Fig. 1c). Through these results, it can be seen that, like the wild-type Cas12a nickase, the en-Cas12a (R1226A) variant also mainly cleaves the non-target strand and induces nick formation on the target DNA (Supplementary Fig. 1d).

### Optimization of the distance and direction between dual Cas12a (R1226A) modules to effectively induce mutations in target DNA

**Figure 2:**
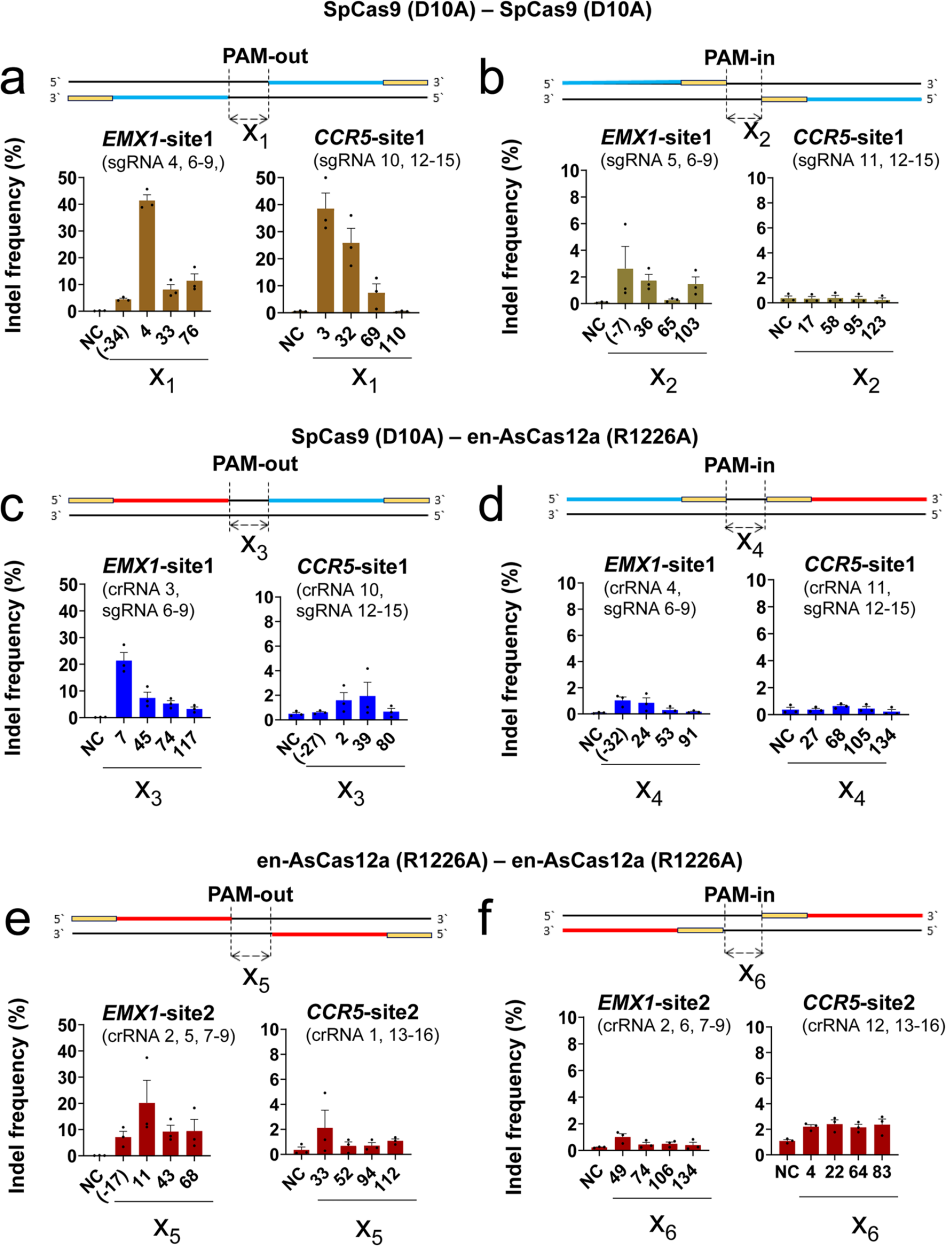
Optimization of cellular gene mutation by en-AsCas12a (R1226A). (**a**–**f**) Comparison of mutation induction efficiency on target genes (*EMX1, CCR5*) in human derived cell line (HeLa) by using SpCas9 (D10A) or en-AsCas12a nickase (R1226A) with PAM orientation dependency. (**a**, **c**, **e**) Comparison of mutation induction efficiency for target genes (*EMX1* and *CCR5*) using the dual nickase (dual SpCas9 (D10A) nickase (**a**), dual SpCas9 (D10A)-en-AsCas12a (R1226A) nickase combination (**c**), dual en-AsCas12a (R1226A) nickase (**e**)) method of PAM-out direction. (**b**, **d**, **f**) Comparison of mutation induction efficiency for target genes (*EMX1* and *CCR5*) using the dual nickase (dual SpCas9 (D10A) nickase (**b**), dual SpCas9 (D10A)-en-AsCas12a (R1226A) nickase combination (**d**), dual en-AsCas12a (R1226A) nickase (**f**)) method of PAM-in direction. Information about the targeted gene sites is given in parentheses under each gene name with the guide RNA number, and the corresponding sequences are given in Supplementary Tables 1–3. NC: negative control, X: end-to-end distance (bp) between protospacers for PAM-out orientation and between PAMs for PAM-in orientation, respectively. Negative value (-X) indicates overlapping between two targets. PAM sequences are indicated in yellow color. Protospacers for SpCas9 (D10A) and en-AsCas12a (R1226A) are indicated in blue and red, respectively. Each histogram was plotted by applying standard error of the mean values to three repeated experimental values (n = 3). SpCas9 (D10A): SpCas9 nickase, en-AsCas12a (R1226A): en-AsCas12a based nickase.

Based on these cleavage test results, target-specific mutations were induced on the target gene in a human-derived cell line (HeLa) using the dual-nickase-type AsCas12a (R1226A) or SpCas9 (D10A) effector (Fig. 2, Supplementary Fig. 2). Since en-AsCas12a, an improved form of wt-AsCas12a, has shown overall superior gene editing properties in human-derived cell lines in previous studies^18^, all subsequent experiments testing Cas12a nickase-related activity were based on the en-AsCas12a effector. To confirm the directionality issue of the CRISPR nickases, the mutation induction efficiency (%) was compared for the different combination according to the directionality of each nickase (Fig. 2, Supplementary Fig. 2a,b). For each combination of SpCas9 (D10A) and en-AsCas12a (R1226A) nickase, the indel frequency (%) in the target genes (*EMX1, CCR5*), which is induced in a PAM-out (Fig. 2a,c,e, Supplementary Fig. 2c) and PAM-in (Fig. 2b,d,f, Supplementary Fig. 2c) fashion, was analyzed by targeted amplicon sequencing (Supplementary Figs. 3, 6, Table 3). Due to the limited issue of PAM (TTTN) recognition when applying en-AsCas12a (R1226A)-based dual nickase at each gene site, we compared the gene editing efficiency at different locations. Consequently, the indel frequency (%) induced in the direction of the PAM-out fashion (mean 12.4% for *EMX1* and 5.8% for *CCR5*), (Fig. 2a,c,e) was higher than that in the PAM-in fashion (mean 0.9% for *EMX1* and 1.0% for *CCR5*), (Fig. 2b,d,f) for all nickase combinations. Particularly, as the distance between the PAMs of nickase combinations increased, the overall indel frequency (%) decreased; a significant difference was observed depending on the nucleotide sequence in the target gene (*EMX1, CCR5*) and orientation between nickases (Fig. 2, Supplementary Fig. 2c). Most of the induced mutation pattern shows large deletions, which is possibly generated by tandem nicking of sequential nickase binding (Supplementary Figs. 3, 6).

### Comparison of the efficiency of mutation induction at target sites between dual- and single-Cas12a (R1226A) variants

**Figure 3:**
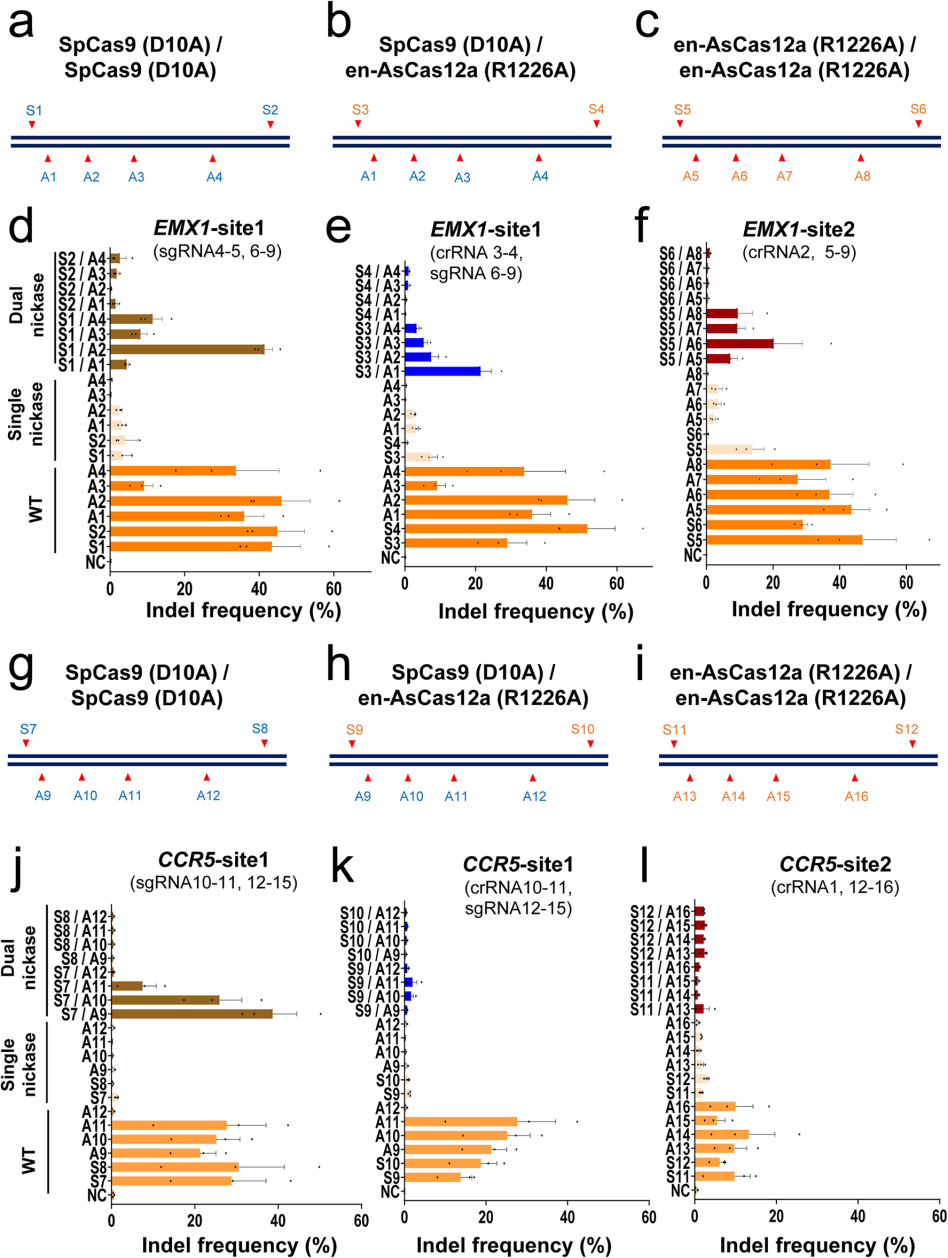
Direct comparison of mutagenic property in cell line for mono- or dual-nicking methods based on en-AsCas12a (R1226A) module. (**a**–**l**) Comparison of mutation induction efficiency of *EMX* and *CCR5* in cell line (HeLa) with different combinations of SpCas9 (D10A) or en-AsCas12a nickase (R1226A). (**a**–**c**) Schematic diagram of the target nucleotide sequence when mutations in the target gene (*EMX1*) are induced using the mono- or dual-nickase (SpCas9 (D10A) nickase only (**a**), SpCas9 (D10A) or en-AsCas12a (R1226A) nickase (**b**), en-AsCas12a (R1226A) nickase only (**c**)) method. Targeted positions are indicated in red arrowhead (Supplementary Tables 1–3), and discriminated by blue (SpCas9 (D10A), S1-S2, A1-A4) and orange (en-AsCas12a (R1226A), S3-S6, A5-A8) number, respectively. S1/A1-4, S3/A1-4, S5/A5-8: PAM-out orientation, S2/A1-4, S4/A1-4, S6/A5-8: PAM-in orientation. (**d**–**f**) Comparison of intracellular gene mutagenesis efficiencies (**d**–**f**) corresponding to mono- or dual-nickase targeting (**a**–**c**). (**g–i**) A schematic diagram of the target nucleotide sequence when mutations in the target gene (*CCR5*) are induced using the mono- or dual-nickase method [SpCas9 (D10A) nickase only (**g**), SpCas9 (D10A) or en-AsCas12a (R1226A) nickase (**h**), en-AsCas12a (R1226A) nickase only (**i**)]. Targeted positions are indicated in red arrowhead, and discriminated by blue (SpCas9 (D10A), S7-S8, A9-A12) and orange (en-AsCas12a (R1226A), S9-S12, A13-A16) number, respectively. S7/A9-12, S9/A9-12, S11/A13-16: PAM-out orientation, S8/A9-12, S10/A9-12, S12/A13-16: PAM-in orientation. (**j–l**) Comparison of intracellular gene mutagenesis efficiencies (**j**–**l**) corresponding to mono- or dual-nickase targeting (**g**–**i**). Indel frequency (%) from dual nickase (SpCas9 (D10A), dual nickase (SpCas9 (D10A) and en-AsCas12a (R1226A), dual nickase (en-AsCas12a (R1226A), single nickase and wild-type nuclease are indicated by brown, blue, red, peach, and orange color, respectively. NC: negative control. Each histogram was plotted by applying standard error of the mean values to three repeated experimental values (n = 3). SpCas9 (D10A): SpCas9 nickase, en-AsCas12a (R1226A): en-AsCas12a based nickase.

The indel frequency (%) induced by the combination of dual nickases, each optimized with respect to PAM orientation, was compared with that induced by a single nickase or WT effector (Fig. 3). Most of the mutations formed by treatment with the single/dual nickase or WT effector were deletions (Supplementary Figs. 4, 6). The mutation frequency was highest for wt-enCas12a (mean 37.6% for *EMX1* and 10.8% for *CCR5*), followed by dual-Cas12a (R1226A) (mean 6.6% for *EMX1* and 3.5% for *CCR5*) and single-Cas12a (R1226A) (mean 2.1% for *EMX1* and 0.6% for *CCR5*) (Fig. 3, Supplementary Figs. 3, 4, 6). As shown in Fig. 2, mutations were only effectively induced when binding in the PAM-out configuration was adopted using dual nickases (Fig. 3d–f,j, top). Notably, the targeted mutations formed by a single nickase were also observed for the dual nickase (Fig. 3, Supplementary Figs. 3, 4). The tendency of single nickase targeted mutation appears to be increased by the treatment of dual nickase rather than only single nickase treated (Fig. 3, Supplementary Figs. 3, 4, 6). Notably, when using the en-AsCas12a (R1226A) effector in a single nickase fashion, the editing efficiency (1.4–3.9%) was similar to that of the previously reported SpCas9 (D10A) nickase (0.4–2.2%), (Fig. 3d–f,j–l middle). Although the indel ratio (%) was lower than that for dual nickase, which is guided by two crRNAs, substantial indels (mean 2.1% for *EMX1* and 0.6% for *CCR5*) were induced by intracellular delivery with the single nickase en-AsCas12a (R1226A) using one crRNA.

### Target-specific genome editing with decreased off-target activity using the dual-en-AsCas12a (R1226A) variant

**Figure 4:**
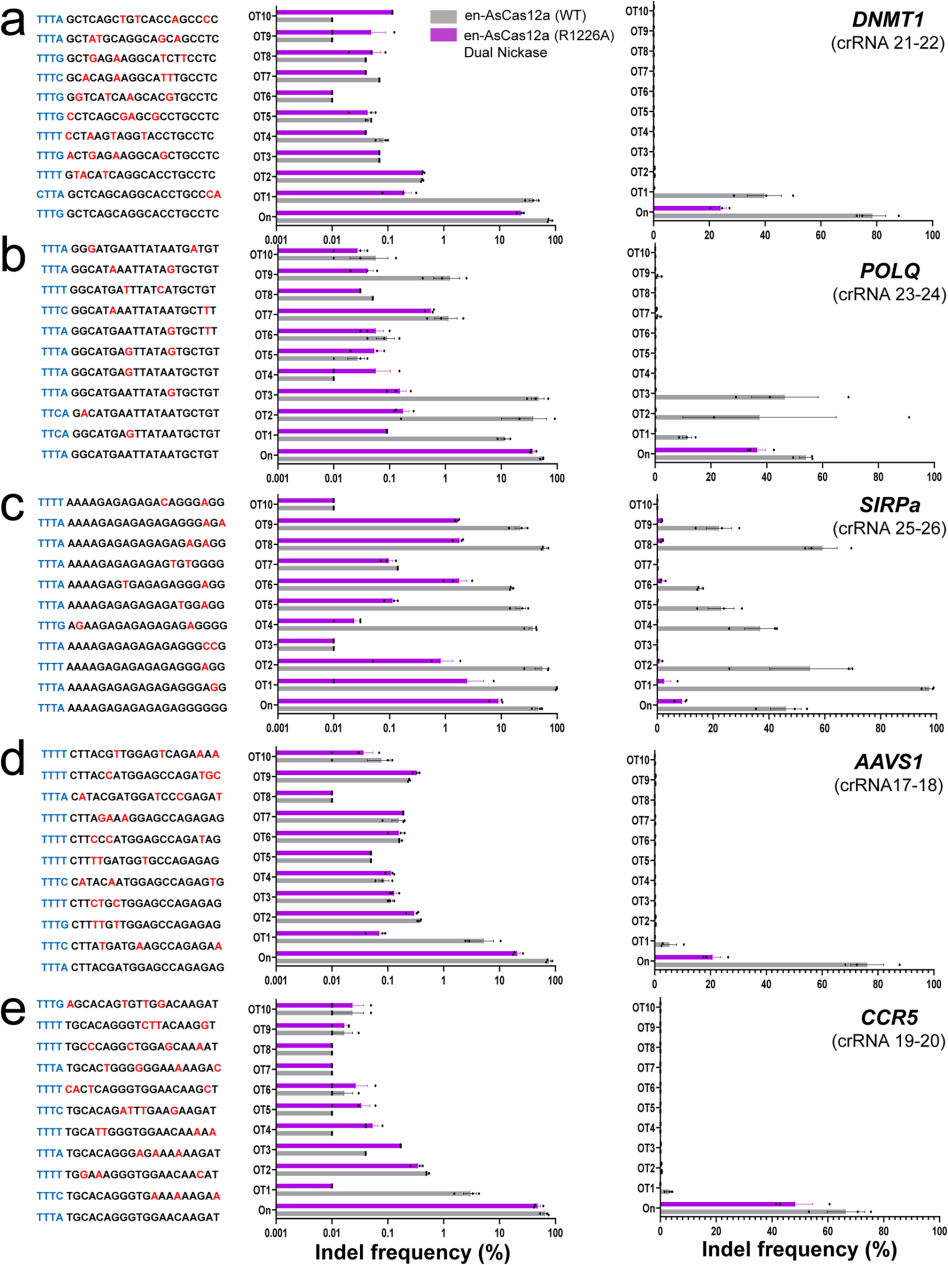
Comparison of on/off-target mutation induction between mono- or dual-en-AsCas12a (R1226A) and wild-type en-AsCas12a. (**a**–**e**) Left: Off-target candidate sequences selected *in-silico* for each target sequence (*DNMT1* (**a**), *POLQ* (**b**), *SIRPa* (**c**), *AAVS1* (**d**), and *CCR5* (**e**)). The PAM sequence and mismatched bases between the target and off-target sequences are shown in cyon and red, respectively. Middle and Right: Histogram showing each indel frequency (%) induced for target sequences and corresponding off-target sites by wild-type en-AsCas12a (WT) and dual-en-AsCas12a (R1226A). On and OT1-10 indicates the on- / off-target sites for each targeted locus (*DNMT1* (**a**), *POLQ* (**b**), *SIRPa* (**c**), *AAVS1* (**d**), and *CCR5* (**e**)). The middle and right histograms show the on- and off-target indel ratio (%) values of wild-type en-AsCas12a and dual nickase en-AsCas12a for the target genes in log-scale and actual values, respectively. Information about the targeted gene sites is given in parentheses under each gene name with the guide RNA number, and the corresponding sequences are given in Supplementary Tables 1–3. Each histogram was plotted by applying standard error of the mean values to three repeated experimental values (n = 3).

To determine whether unintended mutations are induced (i.e., off-target effects) by the dual- and single-en-Cas12a (R1226A) variants, on-/off-target indel ratio (%) was investigated for five target loci (*DNMT1*, *POLQ*, *SIRPa*, *AAVS1*, and *CCR5*) (Fig. 4, Supplementary Figs. 5, 7). First, off-target candidates with up to 3 mismatched sequences compare to those of each target gene were selected based on an *in-silico* analysis^19^, and targeted amplicons were prepared for each predicted off-target site (Fig. 4a–e, left). Then, the mutation frequency at each off-target site (%) was investigated by targeted amplicon sequencing (see Methods section, Supplementary Table 3). The indel mutations generated by dual en-Cas12a (R1226A) variants for each on-target and predicted off-target sites were compared with that of the wild-type en-Cas12a effector, and histograms were obtained (Fig. 4a–e, right). In a comparison of five target loci, the dual en-Cas12a (R1226A) nickase showed an editing efficiency of 27.7%, on average, compared with estimates of 64.2% for the wild-type en-Cas12a effector (WT). In comparison, for the selected off-target site, indel frequencies of 0.26% was induced by dual en-Cas12a (R1226A) variants, compared to a frequency of 9.14% for the wild-type en-Cas12a effector (WT) (Fig. 4a–e, right).

## Discussion

The structures of the target DNA and guide RNA-Cas12a complex revealed to date are similar to Cas9 in that they operate in a bi-lobed form^15,20,21^. However, the cleavage domains (RuvC), which cut the target sequence after PAM recognition or R-loop formation, are located in the target DNA in different forms. Based on these structures, the Cas12a effector has a fundamentally different target DNA cleavage mechanism from that of Cas9. Recently, a process for cleaving a target nucleotide sequence was proposed based on the structure of Cas12a and biochemical experiments^22,23^. Unlike Cas9, the Cas12a complex uses a single cleavage domain (RuvC) and is thought to induce double-strand breaks with successive cleavage of DNA strands. Among the variants obtained by the introduction of mutations in the cleavage domain (RuvC), the R1226A form nicks the target DNA^15^. Our *in-vitro* cleavage study reproduced the result that the Cas12a (R1226A) variant can induce non-target strand cleavage, and it was hypothesized that it forms a nick due to the difficulty in accessing the target strand after the cleavage of non-target strand by RuvC domain^22^. Consistent with this cleavage mechanism, the Cas12a (R1226A) variant that induces nicking to the target DNA remains weakly capable of inducing double-strand cleavage and is weakly identified as a background form in the results (Fig. 1f). Due to these mutations, the Cas12a (R1226A) variant has a very low DNA targeting efficiency (Supplementary Fig. 8), so in this study, we used the enhanced Cas12a (R1226A) variant to effectively induce mutation of target DNA in human-derived cell lines, although concentration-saturated *in-vitro* cleavage experiments show similar effects (Fig. 1c). Similar to previous results for Cas9-based nickases (D10A, H840A)^5^, two Cas12a (R1226A) nickases were bound in tandem to induce an effect similar to DNA repair following double-strand break in the target DNA. Gene mutations were effectively induced (20.7% for *AAVS1,* 48.2% for *CCR5,* and 24% for *DNMT1*). There was a significant difference in genome editing according to the directionality and distance between the dual Cas12a (R1226A) variants (PAM out: 6.3%, PAM in: 1.4%), similar to the experimental results for a Cas9-based nickase (D10A). To effectively induce indels on the target DNA, the directionality and distance between nickases were optimized. The Cas12a (R1226A) variant-based editing system had a few key advances. By using the dual Cas12a (R1226A) variant or a combination of the established Cas9 and dual Cas12a (R1226A) variant, it was possible to accurately induce indels by simultaneously nicking the T-rich target sequence, which was difficult to target with Cas9 alone. The previously established nickase tool has been extended in terms of PAM recognition, and Cas12a nickase variants with extended PAM recognition have the potential to further expand the targeting range of gene editing (Supplementary Fig. 9)^18,24^. Notably, when a single Cas12a (R1226A) nickase was used, a target-specific mutation was induced. Hence, first, the Cas12a (R1226A) variant, which operates as a single cleavage domain, has the potential to induce unintended double-strand breaks with a low probability. Second, as described in a recent paper, indels are also induced by nicks intentionally generated on the target by the Cas12a (R1226A) variant when multiple nicks occur during DNA replication or transcription, which can mimics double-strand break-triggered DNA repair^25^. Using this single Cas12a (R1226A) variant, it is thought that precise genome editing is possible by exogenously delivered donor-based homologous recombination or inter-homolog repair in the future^25,26^. The results of this study also demonstrated that the dual en-Cas12a (R1226A) variant has the potential to decrease the unintended off-target editing compared to that of wt-enCas12a. Although the average on-target mutagenesis efficiency was lower than that of wt-en-Cas12a, we significantly reduced off-target effects by applying the principle that indels can be induced only by the simultaneous induction of dual nicks near the off-target sequence. This shows that the Cas12a-based dual nickase system can be an another effective method to overcome the off-target effects caused by the Cas12a system itself, as it is less sensitive to mismatch formation between crRNA and target DNA at PAM distal region^27^. It is thought that this principle can be applied to other recently reported type V CRISPR-Cas systems able to induce target DNA mutations^28^.

Herein, we proposed a method for target gene editing by single or dual nicking based on the CRISPR-Cas12a system. Our results demonstrate the possibility of safely inducing mutations by single nick generation in vivo, unlike correction using homologous directed repair and non-homologous end joining after inducing DNA double-strand breaks. Furthermore, it provides a basis for replacing or supplementing technologies derived from the previous Guanine-rich PAM recognition-based CRISPR-Cas9 nickase, such as base editing^8^ or prime editing^9^. This study is expected to contribute to the induction of safe and efficient gene editing in various biological systems by complementing the limitations of existing Cas9 nickase-based approaches.

## Methods

### Purification of the recombinant wt-AsCas12a, en-AsCas12a (R1226A), and SpCas9 (D10A, H840A) proteins

For purification of en-AsCas12a (E174R, S542R, K548R), en-AsCas12a nickase (E174R, S542R, K548R, R1226A), and SpCas9 nickase (D10A) recombinant proteins, pET28a bacterial expression vectors encoding each protein were transformed into *E. coli* BL21(DE3) system. Transformed bacterial colonies were induced with isopropylthio-β-galactoside after culturing them in a 500 ml flask at 37 °C to OD = 0.6. After 48 h, the cultured bacteria were precipitated by centrifugation at 10,000x*g* at 4 °C; the culture medium was removed to obtain a bacterial pellet. Then, the pellets were treated with lysis buffer [20 mM Tris–HCl (pH 8.0), 300 mM NaCl, 1 mM PMSF, 10 mM β-mercaptoethanol, 1% TritonX-100] to obtain intracellular proteins and dissolving the bacterial cell membrane. To effectively remove the bacterial cell membrane, lysates were subjected to sonication twice in ice water for 3 min (Qsonica, model Q700, Amplitude: 30, Process time: 3 min) and then centrifuged for 10 min at 5,000 rpm (4 °C). Nitrilotriacetic acid (Ni–NTA) resin was pretreated with wash buffer [300 mM NaCl, 20 mM Tris–HCl (pH 8.0)] to purify the (6x) His-tag-linked target recombinant protein using an affinity column. The pellet of the centrifuged solution was discarded, and the remaining supernatant and the pretreated resin were reacted at 4 °C for 90 min. To remove non-specific binding components from the cell lysate solution mixed with Ni–NTA resin, washing was performed using a wash buffer ten times the volume of the lysate. Finally, to elute the target recombinant protein, the protein was separated from the Ni–NTA resin using an elution buffer [20 mM Tris–HCl (pH 8.0), 200 mM imidazole, 300 nM NaCl]; the eluted protein solution was concentrated (Amicon Ultra) and storage buffer [200 mM NaCl, 40% glycerol, 50 mM HEPES (pH 7.5), 1 mM DTT], aliquoted and stored at -80 °C.

### In-vitro transcription for guide RNA synthesis

To prepare crRNA and single-guide RNA (sgRNA) using *in-vitro* transcription (Supplementary Tables 1 and 2), sense and antisense DNA oligos, including target sequences for each guide RNA, were purchased from Macrogen. Sense and antisense DNA oligos were annealed under specific PCR conditions. After mixing the annealed DNA template with T7 RNA polymerase (NEB) and the reaction mixture [50 mM MgCl_2_, 100 mM rNTP (Jena Bio, NU-1014), RNase Inhibitor Murine, DEPC, 100 mM DTT, 10 × RNA polymerase reaction buffer], the reaction was carried out at 37 °C for 8 h. After the reaction, the DNA template was completely removed by reaction with DNase at 37 °C for 1 h, and only RNA was isolated using a column (MP Biomedicals, GENECLEAN® Turbo Kit). The purified RNA was lyophilized (2,000 rpm, -55 °C, 1 h) and stored for later use.

### In-vitro cleavage assay for nicking property analysis

PCR amplicons containing the target sequence were obtained from purified genomic DNA (HeLa cells) using DNA primers (PCR reaction conditions, Supplementary Table 3). Then, PCR amplicons were inserted into T-blunt vector (Solgent), transformed into DH-5α *E. coli* cells, purified using the Plasmid Maxi Kit (Qiagen), and used as a DNA template vector for cleavage experiments. The template vector (20 ng/μL) was incubated with purified recombinant Cas12a protein (50 ng/μL) and crRNA (75 ng/μL) complex at 37 °C for 1 h. Plasmid cleavage was confirmed by 0.8% agarose gel electrophoresis of the reaction mixture.

### Cell culture and transfection

To verify that the en-AsCas12a-based nickase (R1226A) system works on intracellular genes, the CMV promoter-based en-AsCas12a (R1226A) expression vector was delivered into the human-derived cell line (HeLa, ATCC (American Type Culture Collection) No. CCL-2). Basically, CMV-enAsCas12a (WT), CMV-enAsCas12a (R1226A), CMV-SpCas9 (WT), CMV-SpCas9 (D10A) vectors, and the corresponding U6-sgRNA (SpCas9) and U6-crRNA (en-AsCas12a) vectors were mixed and delivered into human-derived cell lines (HeLa, ATCC (American Type Culture Collection) No. CCL-2) to target nucleotide sequence on each genomic locus (*CCR5 and EMX1*). The HeLa cell line was subcultured in DMEM media [DMEM (Gibco), 10% FBS (Gibco)] every 48 h at 37 °C and 5% CO_2_ while maintaining 70% confluency. 1 × 10^5^ HeLa cells were seeded in a 24-well plate one day before transfection. On the day of transfection, a mixture of CMV-en-AsCas12a vector (1 µg), U6-crRNA vector (333 ng /CMV-SpCas9 vector (1 µg), U6-sgRNA vector (333 ng)/CMV-en-AsCas12a vector (1 µg), U6-crRNA vector (333 ng), CMV-SpCas9 vector (1 µg), and U6-sgRNA vector (333 ng) were delivered into the cells using 1.4 μL of lipofectamine (ThermoFisher) and 1 μL of P3000 reagent following the manufacturer’s protocols. Then, 48 h after transfection, genomic DNA was purified using a genomic DNA purification kit (Qiagen, DNeasy Blood & Tissue Kit) to analyze the editing efficiency of the targeted locus.

### Targeted amplicon sequencing and data analysis

To check whether the mutation of the target nucleotide sequence in the gene (*CCR5* and *EMX1*) was induced by the gene editing approaches, next-generation sequencing was performed. Initially, PCR was performed (denaturation: 98 °C for 30 s, primer annealing: 60 °C for 15 s, and elongation: 72 °C for 15 s for 35 cycles) using a DNA primer (Supplementary Table 3) corresponding to each gene to obtain a DNA amplicon. For barcoding each DNA amplicon, nested PCR was conducted to connect the adapter and index sequences at both ends of the amplicon (denaturation: 98 °C for 30 s, primer annealing: 62 °C for 15 s, and elongation: 72 °C for 15 s for 35 cycles). According to the manufacturer's manual, the amplicon mixture linked to the index sequence was pretreated and loaded into a mini-SEQ analyzer (Illumina MiniSeq system, SY-420-1001) to perform targeted amplicon sequencing. The saved Fastaq file was analyzed using Cas-Analyzer^29^, a web-based analysis tool, and the mutation induction efficiency (%) of the target gene was calculated (for Supplementary Figs. 2, 3, 4, 5). In addition, each indel frequency was calculated using CRISPResso2^30^ and converted into a phi-chart (Supplementary Figs. 6, 7).

## *In-silico* analysis of CRISPR effector target coverage

For target sites with Cas9 or Cas12a PAMs, we took into account that genome editing involves cleavage of DNA near NGG or TTTN PAMs and can be targeted in a sense / anti-sense direction because DNA is double helix. Therefore, for Cas9, the probability of existance of the 5'-NGG-3' sequence within 5 bases from the target base when targeting in the sense strand direction, or the probability of existance of 5'-CCN-3' when targeting in the anti-sense strand direction was calculated, respectively. Next, the probability of the AsCas12a targeting range was calculated by PAM existance of 5'-TTTN-3' or 5'-NAAA-3' sequence being present in the target site based on the sense or anti-sense manner. The probability of a target site containing both SpCas9 and AsCas12a PAMs and the probability of a site containing neither PAM was calculated as the same way.

### Supplementary Information

Supplementary Information.

## Data Availability

All the relevant data support the findings of this study are available from the corresponding author upon reasonable request. All the targeted amplicon sequencing data were deposited at NCBI Sequence Reads Archive database with accession number SRP339187.
