# Supplementary material for: Utilization of nicking properties of CRISPR-Cas12a effector for genome editing
Source: Sci Rep. 2024 Feb 9;14:3352. doi: 10.1038/s41598-024-53648-2 (PMC10858195; doi:10.1038/s41598-024-53648-2)
Supplement: Supplementary file 1 — Supplementary Information. [file 41598_2024_53648_MOESM1_ESM.pdf]

Utilization of nicking properties of CRISPR-Cas12a effector for genome editing

Chan Hyoung Kim, Wi-jae Lee, Yeounsun Oh, Youngjeon Lee, Hyomin K. Lee, Jung Bae Seong, Kyung-Seob Lim, Sang Je Park, Jae-Won Huh, Young-Hyun Kim, Kyoung Mi Kim, Junho K. Hur and Seung Hwan Lee

Supplementary Tables

Supplementary Table 1. Sequence information for crRNAs of CRISPR-Cas12a used in this study. Target sequences on the chromosome and each crRNA of en-AsCas12a for corresponding targets are indicated. PAM sequences (TTTN) in the target DNA are shown in cyon.

Target gene (guide no.)	CRISPR-Cas12a (enAsCas12a) target sequence (5′-3′)	crRNA sequence for enAsCas12a (5′-3′)
hCCR5- site2 **crRNA1(S11)**	TTTCATGAATTCCCCCAACAGAGCCAAG	**5'UAAUUUCUACUCUUGUAGAUAUGAAUUCCCCCAACAGAGCCAAG 3'**
hEMX1-site2 **crRNA 2(A6)**	TTTCCCTTACGACATCCAGACCCCTCTC	**5'UAAUUUCUACUCUUGUAGAUCCUUACGACAUCCAGACCCCUCUC 3'**
hEMX1- site1 **crRNA3(S3)**	TTTGTGGTTGCCCACCCTAGTCATTGGA	**5'UAAUUUCUACUCUUGUAGAUUGGUUGCCCACCCUAGUCAUUGGA** 3'
hEMX1- site1 **crRNA4(S4)**	TTTGTACTTTGTCCTCCGGTTCTGGAAC	**5'UAAUUUCUACUCUUGUAGAUUACUUUGUCCUCCGGUUCUGGAAC3'**
hEMX1- site2 **crRNA5(S5)**	TTTCATGAGCACATGGGAGGTGAAGAAG	**5'UAAUUUCUACUCUUGUAGAUAUGAGCACAUGGGAGGUGAAGAAG 3'**
hEMX1- site2 **crRNA6(S6)**	TTTCTTGGGCTGAGGAGGCCCTGAGTCA	**5'UAAUUUCUACUCUUGUAGAUUUGGGCUGAGGAGGCCCUGAGUCA 3'**

hEMX1- site2<br>**crRNA7(A5)**	TTTCTTCTTCACCTCCCATGTGCTCATG	**5'UAAUUUCUACUCUUGUAGAUUUCUUCA<br>CCUCCCAUGUGCUCAUG 3'**
hEMX1- site2<br>**crRNA8(A7)**	TTTCCTGACAAGCAAATCTGCATTCCTA	**5'UAAUUUCUACUCUUGUAGAUCUGACAA<br>GCAAAUCUGCAUUCCUA 3'**
hEMX1- site2<br>**crRNA9(A8)**	TTTGTCCCACTTGTCAGGATGAAGGTTT	**5'UAAUUUCUACUCUUGUAGAUUCCCACU<br>UGUCAGGAUGAAGGUUU 3'**
hCCR5- site1<br>**crRNA10(S9)**	TTTTGTGGGCAACATGCTGGTCATCCTC	**5'UAAUUUCUACUCUUGUAGAUGUGGGCA<br>ACAUGCUGGUCAUCCUC 3'**
hCCR5- site1<br>**crRNA11(S10)**	TTTGGAAATACAATGTGTCAACTCTTGA	**5'UAAUUUCUACUCUUGUAGAUGAAAUAC<br>AAUGUGUCAACUCUUGA 3'**
hCCR5- site2<br>**crRNA1(S11)**	TTTCATGAATTCCCCCAACAGAGCCAAG	**5'UAAUUUCUACUCUUGUAGAUAUGAAUU<br>CCCCCAACAGAGCCAAG 3'**
hCCR5- site2<br>**crRNA12(S12)**	TTTGCATTCATGGAGGGCAACTAAATAC	**5'UAAUUUCUACUCUUGUAGAUCAUUCAU<br>GGAGGGCAACUAAAUAC 3'**
hCCR5- site2<br>**crRNA13(A13)**	TTTGTAGTGAAGGGAAGGTTTGCTGCTA	**5'UAAUUUCUACUCUUGUAGAUUAGUGAA<br>GGGAAGGUUUGCUGCUA 3'**
hCCR5- site2<br>**crRNA14(A14)**	TTTGGCCAAGCAATGAAGTTTTGTAGTG	**5'UAAUUUCUACUCUUGUAGAUGCCAAGC<br>AAUGAAGUUUUGUAGUG 3'**
hCCR5- site2<br>**crRNA15(A15)**	TTTAATTGCCTACATAGATGTCTACATT	**5'UAAUUUCUACUCUUGUAGAUAUUGCCU<br>ACAUAGAUGUCUACAUU 3'**
hCCR5- site2<br>**crRNA16(A16)**	TTTATACATCAATAGGTTTTTAATTGCC	**5'UAAUUUCUACUCUUGUAGAUUACAUCA<br>AUAGGUUUUUAAUUGCC 3'**
hAAVS1<br>**crRNA17(S20)**	CTCCCACCCCCTGCCAAGCTCTCCCTCC	**5'UAAUUUCUACUCUUGUAGAUCACCCCC<br>UGCCAAGCUCUCCCUCC 3`**
hAAVS1<br>**crRNA18(A20)**	TTTGCTTACGATGGAGCCAGAGAGGATC	**5'UAAUUUCUACUCUUGUAGAUCUUACGA<br>UGGAGCCAGAGAGGAUC 3`**
hCCR5<br>**crRNA19(S21)**	TTTATGCACAGGGTGGAACAAGATGGAT	**5'UAAUUUCUACUCUUGUAGAUUGCACAG<br>GGUGGAACAAGAUGGAU 3`**
hCCR5<br>**crRNA20(A21)**	TTTGGCAGGGCTCCGATGTATAATAATT	**5'UAAUUUCUACUCUUGUAGAUGCAGGGC<br>UCCGAUGUAUAAUAAUU 3`**
hDNMT1<br>**crRNA21(S22)**	GTTCTAGACCCAGAGGCTCAAGTGAGCA	**5'UAAUUUCUACUCUUGUAGAUUAGACCC<br>AGAGGCUCAAGUGAGCA 3`**
hDNMT1<br>**crRNA22(A22)**	TTTGGCTCAGCAGGCACCTGCCTCAGCT	**5'UAAUUUCUACUCUUGUAGAUGCUCAGC<br>AGGCACCUGCCUCAGCU 3`**
hPOLQ<br>**crRNA23(S23)**	TTTAGGCATGAATTATAATGCTGTTGGC	**5'UAAUUUCUACUCUUGUAGAUGGCAUGA<br>AUUAUAAUGCUGUUGGC 3`**
hPOLQ<br>**crRNA24(A23)**	TTTATGCATGTTTCTGTTTAAAGACATC	**5'UAAUUUCUACUCUUGUAGAUUGCAUGU<br>UUCUGUUUAAAGACAUC 3`**
hSIRPa<br>**crRNA25(S24)**	TTTAAAAAGAGAGAGAGAGGGGGGAAAC	**5'UAAUUUCUACUCUUGUAGAUAAAAGAG<br>AGAGAGAGGGGGGAAAC 3`**

hSIRPa **crRNA26(A24)**	TTTTCTTATTTGTAGTCTTGGACTGACG	**5'UAAUUUCUACUCUUGUAGAUCUUAUUU GUAGUCUUGGACUGACG 3`**

Supplementary Table 2. Sequence information for single-guide RNA (sgRNA) of CRISPR-Cas9 used in this study. Target sequences on the chromosome and each sgRNA of SpCas9 for corresponding targets are indicated. PAM sequences (NGG) in the target DNA are shown in red.

Target gene (guide No.)	CRISPR-Cas9 (SpCas9) target sequence (5′-3′)	sgRNA sequence for SpCas9 (5′-3′)
hCCR5-site2 **sgRNA1**	TGTCCACTAGATGGAGAGCTTGG	**5'GUGUCCACUAGAUGGAGAGCUGUUUUAGAGCUAGA AAUAGCAAGUUAAAAUAAGGCUAGUCCGUUAUCAAC UUGAAAAAGUGGCACCGAGUCGGUGC** 3'
hEMX1-site2 **sgRNA2**	GGAATGCAGATTTGCTTGTCAGG	**5'GGGAAUGCAGAUUUGCUUGUCGUUUUAGAGCUAGA AAUAGCAAGUUAAAAUAAGGCUAGUCCGUUAUCAAC UUGAAAAAGUGGCACCGAGUCGGUGC 3'**
hCCR5-site2 **sgRNA3**	AGCTCTCCATCTAGTGGACAGGG	**5'GAGCUCUCCAUCUAGUGGACAGUUUUAGAGCUAGA AAUAGCAAGUUAAAAUAAGGCUAGUCCGUUAUCAAC UUGAAAAAGUGGCACCGAGUCGGUGC** 3'
hEMX1- site1 **sgRNA4(S1)**	CACGAAGCAGGCCAATGGGGAGG	**5'GCACGAAGCAGGCCAAUGGGGGUUUUAGAGCUAGA AAUAGCAAGUUAAAAUAAGGCUAGUCCGUUAUCAAC UUGAAAAAGUGGCACCGAGUCGGUGC** 3'
hEMX1- site1 **sgRNA5(S2)**	AGGTGTGGTTCCAGAACCGGAGG	**5'GAGGUGUGGUUCCAGAACCGGGUUUUAGAGCUAGA AAUAGCAAGUUAAAAUAAGGCUAGUCCGUUAUCAAC UUGAAAAAGUGGCACCGAGUCGGUGC** 3'
hEMX1- site1 **sgRNA6(A1)**	GACATCGATGTCCTCCCCATTGG	**5`GGACAUCGAUGUCCUCCCCAUGUUUUAGAGCUAGA AAUAGCAAGUUAAAAUAAGGCUAGUCCGUUAUCAAC UUGAAAAAGUGGCACCGAGUCGGUGC 3`**
hEMX1- site1 **sgRNA7(A2)**	TGCGCCACCGGTTGATGTGATGG	**5'GUGCGCCACCGGUUGAUGUGAGUUUUAGAGCUAGA AAUAGCAAGUUAAAAUAAGGCUAGUCCGUUAUCAAC UUGAAAAAGUGGCACCGAGUCGGUGC** 3'
hEMX1- site1 **sgRNA8(A3)**	TTCTTCTTCTGCTCGGACTCAGG	**5'GUUCUUCUUCUGCUCGGACUCGUUUUAGAGCUAGA AAUAGCAAGUUAAAAUAAGGCUAGUCCGUUAUCAAC UUGAAAAAGUGGCACCGAGUCGGUGC** 3'
hEMX1- site1 **sgRNA9(A4)**	GCCGTTTGTACTTTGTCCTCCGG	**5`GGCCGUUUGUACUUUGUCCUCGUUUUAGAGCUAGA AAUAGCAAGUUAAAAUAAGGCUAGUCCGUUAUCAAC UUGAAAAAGUGGCACCGAGUCGGUGC 3`**
hCCR5- site1 **sgRNA10(S7)**	ATGAACACCAGTGAGTAGAGCGG	**5'GAACACCAGUGAGUAGAGCGGGUUUUAGAGCUAGA AAUAGCAAGUUAAAAUAAGGCUAGUCCGUUAUCAAC UUGAAAAAGUGGCACCGAGUCGGUGC** 3'
hCCR5- site1 **sgRNA11(S8)**	TTGTATTTCCAAAGTCCCACTGG	**5'GUUGUAUUUCCAAAGUCCCACGUUUUAGAGCUAGA AAUAGCAAGUUAAAAUAAGGCUAGUCCGUUAUCAAC UUGAAAAAGUGGCACCGAGUCGGUGC** 3'

hCCR5- site1 **sgRNA12(A9)**	TGGTTTTGTGGGCAACATGCTGG	**5'GUGGUUUUGUGGGCAACAUGCGUUUUAGAGCUAGA AAUAGCAAGUUAAAAUAAGGCUAGUCCGUUAUCAAC UUGAAAAAGUGGCACCGAGUCGGUGC** 3'
hCCR5- site1 **sgRNA13(A10)**	TCATCCTGATAAACTGCAAAAGG	**5'GUCAUCCUGAUAAACUGCAAAGUUUUAGAGCUAGA AAUAGCAAGUUAAAAUAAGGCUAGUCCGUUAUCAAC UUGAAAAAGUGGCACCGAGUCGGUGC** 3'
hCCR5- site1 **sgRNA14(A11)**	TGACATCTACCTGCTCAACCTGG	**5'GUGACAUCUACCUGCUCAACCGUUUUAGAGCUAGA AAUAGCAAGUUAAAAUAAGGCUAGUCCGUUAUCAAC UUGAAAAAGUGGCACCGAGUCGGUGC** 3'
hCCR5- site1 **sgRNA15(A12)**	TCCTTCTTACTGTCCCCTTCTGG	**5'GUCCUUCUUACUGUCCCCUUCGUUUUAGAGCUAGA AAUAGCAAGUUAAAAUAAGGCUAGUCCGUUAUCAAC UUGAAAAAGUGGCACCGAGUCGGUGC** 3'
hEMX1- site1 **sgRNA16**	ATGTCGTAAGGGAAAGACTTAGG	**5`AUGUCGUAAGGGAAAGACUUGUUUUAGAGCUAGAA AUAGCAAGUUAAAAUAAGGCUAGUCCGUUAUCAACU UGAAAAAGUGGCACCGAGUCGGUGC 3`**

Supplementary Table 3. Sequence information for DNA primers used in this study. The sequence information of the primers used to amplify the on- or off-target in this study. The sequence of the forward and reverse adapter primers used in next generation sequencing is colored in green and cyon, respectively.

Target gene (primer direction)	DNA sequence (5′ to 3′)
hEMX1- site1 on-target F1	CTGGGGATCACTAAACTACAGT
hEMX1- site1 on-target R1	CCCTTTGTTTGAGAGGAACAG
hEMX1- site2 on-target F1	CCAGGGAGCCAAAGAGAAGT
hEMX1- site2 on-target R1	CTGGTGGCTTCTCCAGTTGA
hCCR5- site1 on-target F1	TGAGATGGTGCTTTCATGAAT
hCCR5- site1 on-target R1	GAAAATGAGAGCTGCAGGTG
hCCR5- site2 on-target F1	CAAAAAGGCTGAGCTGCACC
hCCR5- site2 on-target R1	GCAGGTAGATGTCAGTCATG
hEMX1- site1_Adaptor_F_V1	ACACTCTTTCCCTACACGACGCTCTTCCGATCTCAAGCAGCACTCTGCCCTC
hEMX1- site1_Adaptor_R_V1	GTGACTGGAGTTCAGACGTGTGCTCTTCCGATCTGGTGAAGGTGTGGTTCCAGA A
hEMX1- site1_Adaptor_F_V2	ACACTCTTTCCCTACACGACGCTCTTCCGATCTAGTCATTGGAGGTGACATCG
hEMX1- site1_Adaptor_R_V2	GTGACTGGAGTTCAGACGTGTGCTCTTCCGATCTCTCCTGAGTTTCTCATCTGTG
hEMX1- site2_Adaptor_F_V1	ACACTCTTTCCCTACACGACGCTCTTCCGATCTCACCGGCAGAATGGTGGCAT
hEMX1- site2_Adaptor_R_V1	GTGACTGGAGTTCAGACGTGTGCTCTTCCGATCTCAGGGCCCGCTGAGCTCTTG
hEMX1- site2_Adaptor_F_V2	ACACTCTTTCCCTACACGACGCTCTTCCGATCTGGGAGGTGAAGAAGAAAGAG
hEMX1- site2_Adaptor_R_V2	GTGACTGGAGTTCAGACGTGTGCTCTTCCGATCTGCTTAAGGCTGAGCCTGCAA
hCCR5- site1_Adaptor_F_V1	ACACTCTTTCCCTACACGACGCTCTTCCGATCT CCTGCCAAAAAATCAATGTGAAG

hCCR5- site1_Adaptor_R_V1	GTGACTGGAGTTCAGACGTGTGCTCTTCCGATCTGAAGGGGACAGTAAGAAGGA A
hCCR5- site1_Adaptor_F_V2	ACACTCTTTCCCTACACGACGCTCTTCCGATCT GCTGGTCATCCTCATCCTG
hCCR5- site1_Adaptor_R_V2	GTGACTGGAGTTCAGACGTGTGCTCTTCCGATCTGAAGATTCCAGAGAAGAAGCC
hCCR5- site1_Adaptor_F_V3	ACACTCTTTCCCTACACGACGCTCTTCCGATCTCGCTCTACTCACTGGTGTTCA
hCCR5- site1_Adaptor_R_V3	GTGACTGGAGTTCAGACGTGTGCTCTTCCGATCTGAGCCCTGTCAAGAGTTGAC
hCCR5- site2_Adaptor_F_V1	ACACTCTTTCCCTACACGACGCTCTTCCGATCTATTTGGTGAGATGGTGCTTTC
hCCR5- site2_Adaptor_R_V1	GTGACTGGAGTTCAGACGTGTGCTCTTCCGATCTCCCTCCATGAATGCAAACTG
hCCR5- site2_Adaptor_F_V2	ACACTCTTTCCCTACACGACGCTCTTCCGATCTCCATCTAGTGGACAGGGAAGC
hCCR5- site2_Adaptor_R_V2	GTGACTGGAGTTCAGACGTGTGCTCTTCCGATCTCCACCCTGTGCATAAATAAAAA G
hAAVS1_on-target F1	CCTGGTGAACACCTAGGACG
hAAVS1_on-target R1	CTATGTCCACTTCAGGACAGC
hCCR5_ on-target F1	ACCATGCTTGACCCAGTTTC
hCCR5_ on-target R1	AAACACAGCATGGACGACAG
hDNMT1_ on-target F1	ACACAACAGCTTCATGTCAG
hDNMT1_ on-target R1	TTGGCTTGGAGATCAAGCTT
hAAVS1_Adaptor_F	ACACTCTTTCCCTACACGACGCTCTTCCGATCTCTTCCCTCCCACCCCCTG
hAAVS1_Adaptor_R	GTGACTGGAGTTCAGACGTGTGCTCTTCCGATCTCCCCATCCTTAGGCCTCCT
hCCR5_Adaptor_F	ACACTCTTTCCCTACACGACGCTCTTCCGATCTCAATGTAGACATCTATGTAGGCA A
hCCR5_Adaptor_R	GTGACTGGAGTTCAGACGTGTGCTCTTCCGATCTCTGCGATTTGCTTCACATTG
hDNMT1_Adaptor_F	ACACTCTTTCCCTACACGACGCTCTTCCGATCTGCAGAGTGCTAAGGGAACGT
hDNMT1_Adaptor_R	GTGACTGGAGTTCAGACGTGTGCTCTTCCGATCTAAGTGCTTAGAGCAGGCGTG
hAAVS1_off-target1_F	ATCCAGGGGGTTGGAATATC
hAAVS1_off-target1_R	TGCCTGAGAGCAGGTCTTTT
hAAVS1_off-target1_Adaptor_F	ACACTCTTTCCCTACACGACGCTCTTCCGATCTGGTTATCTGTTAATGATAGCCTG
hAAVS1_off-target1_Adaptor_R	GTGACTGGAGTTCAGACGTGTGCTCTTCCGATCTCACAAGCCCATGAAGACTGG
hAAVS1_off-target2_F	TCAGCCGGTTGTCACTGTAG
hAAVS1_off-target2_R	GCTGTCTGAGGCACAAATCA
hAAVS1_off-target2_Adaptor_F	ACACTCTTTCCCTACACGACGCTCTTCCGATCTATCTGCAAAATCGGAAATGC
hAAVS1_off-target2_Adaptor_R	GTGACTGGAGTTCAGACGTGTGCTCTTCCGATCTAATGCCTAGCGCAAAGAAGA
hAAVS1_off-target3_F	TTCTATCGTTGTGGCTGCTG
hAAVS1_off-target3_R	CTGTGGCTGCCTGTTGTCTA
hAAVS1_off-target3_Adaptor_F	ACACTCTTTCCCTACACGACGCTCTTCCGATCTTGGCTGGAGTTCTTGGAGTT
hAAVS1_off-target3_Adaptor_R	GTGACTGGAGTTCAGACGTGTGCTCTTCCGATCTAAGGGTGGCAGCAATCTCTA
hAAVS1_off-target4_F	ATGTCGAGGAGATCCTGGTG
hAAVS1_off-target4_R	ACGGCCCCTTCTCTACTCTT
hAAVS1_off-target4_Adaptor_F	ACACTCTTTCCCTACACGACGCTCTTCCGATCTAGAACAGGGAAAAGCCATCA
hAAVS1_off-target4_Adaptor_R	GTGACTGGAGTTCAGACGTGTGCTCTTCCGATCTGTCTCCATCACGACAGCTCA
hAAVS1_off-target5_F	TGCATGCACAGTTCACAAGA
hAAVS1_off-target5_R	AGAGCTGCTCACCAGACCAT
hAAVS1_off-target5_Adaptor_F	ACACTCTTTCCCTACACGACGCTCTTCCGATCTTGCTAAAATGAGCCTTTGGA
hAAVS1_off-target5_Adaptor_R	GTGACTGGAGTTCAGACGTGTGCTCTTCCGATCTGGGAAGGAGAGAGGATGGAC
hAAVS1_off-target6_F	CCACAAGGGCAAGGATTAGA

hAAVS1_off-target6_R	TTTCTTTGGGCAATTTCCAG
hAAVS1_off-target6_Adaptor_F	ACACTCTTTCCCTACACGACGCTCTTCCGATCTTCCACTTGTTATCAACCCATCA
hAAVS1_off-target6_Adaptor_R	GTGACTGGAGTTCAGACGTGTGCTCTTCCGATCTTTGTTCATGTGCTTCCTTGG
hAAVS1_off-target7_F	CAAATCTCTCCTGGCTCCTG
hAAVS1_off-target7_R	CCTGGTGAAGACAGACAGCA
hAAVS1_off-target7_Adaptor_F	ACACTCTTTCCCTACACGACGCTCTTCCGATCTTGGACTTCCATCTCCTGGTC
hAAVS1_off-target7_Adaptor_R	GTGACTGGAGTTCAGACGTGTGCTCTTCCGATCTAGAGAGAGGAAGCGGAGACA
hAAVS1_off-target8_F	CATGGGATAATGGTGGAAGG
hAAVS1_off-target8_R	AAATCAATTGAGGGGACGTG
hAAVS1_off-target8_Adaptor_F	ACACTCTTTCCCTACACGACGCTCTTCCGATCT GCATCACCTGCATCTTTGAA
hAAVS1_off-target8_Adaptor_R	GTGACTGGAGTTCAGACGTGTGCTCTTCCGATCT CTGATCGCCACTTTGCCTAT
hAAVS1_off-target9_F	ATCCTTCCAATGTGGTCCAG
hAAVS1_off-target9_R	GCAGGTATAATCGCGGACAT
hAAVS1_off-target9_Adaptor_F	ACACTCTTTCCCTACACGACGCTCTTCCGATCT TTCAACTGCAGCACACATGA
hAAVS1_off-target9_Adaptor_R	GTGACTGGAGTTCAGACGTGTGCTCTTCCGATCT CCACAACAATGGCTGCTTC
hAAVS1_off-target10_F	CCTCCCTCCACTATGAGCAG
hAAVS1_off-target10_R	TTGTTCTGTCCAAGCCACTG
hAAVS1_off-target10_Adaptor_F	ACACTCTTTCCCTACACGACGCTCTTCCGATCT CAGGGAGGTGAGTAGGGTGA
hAAVS1_off-target10_Adaptor_R	GTGACTGGAGTTCAGACGTGTGCTCTTCCGATCT TCTGCAGTTGGCAGTGAAAG
hCCR5_off-target1_F	CAAGCAATTCTTGTGCCTCA
hCCR5_off-target1_R	TCCAGGCCCTGTATACTTGC
hCCR5_off-target1_Adaptor_F	ACACTCTTTCCCTACACGACGCTCTTCCGATCTAGGGTCAACATTGCAAGGAG
hCCR5_off-target1_Adaptor_R	GTGACTGGAGTTCAGACGTGTGCTCTTCCGATCTTCAAAGCCATTCTGGAAAAGA
hCCR5_off-target2_F	GGCTGCTTCTTGGACTCAAC
hCCR5_off-target2_R	AGCGAGACCCTGTCTCAAAA
hCCR5_off-target2_Adaptor_F	ACACTCTTTCCCTACACGACGCTCTTCCGATCTAGTCGGTCTTTGGTCACTGC
hCCR5_off-target2_Adaptor_R	GTGACTGGAGTTCAGACGTGTGCTCTTCCGATCTTGTCTTCGAAAGTGGAATGC
hCCR5_off-target3_F	CTGCACTTTAGGAGGCCAAG
hCCR5_off-target3_R	CAATCCCAGTTAAGCCCAAA
hCCR5_off-target3_Adaptor_F	ACACTCTTTCCCTACACGACGCTCTTCCGATCTGCAATGTGAAGTCTCGGACA
hCCR5_off-target3_Adaptor_R	GTGACTGGAGTTCAGACGTGTGCTCTTCCGATCTGATAGCCAGTGGGCCTCTC
hCCR5_off-target4_F	CAGAAGCGGAACAGCCTAAC
hCCR5_off-target4_R	CTTGGCTCTGGGCTAAACAG
hCCR5_off-target4_Adaptor_F	ACACTCTTTCCCTACACGACGCTCTTCCGATCT AGCCATTCTGAAGAGCTGGA
hCCR5_off-target4_Adaptor_R	GTGACTGGAGTTCAGACGTGTGCTCTTCCGATCT TCTGAGGACCACCTCCCTTA
hCCR5_off-target5_F	AACTGCTTGCACCCAAGTCT
hCCR5_off-target5_R	TGCTCAGAGATGCCTTTCCT
hCCR5_off-target5_Adaptor_F	ACACTCTTTCCCTACACGACGCTCTTCCGATCT AACTGCTTGCACCCAAGTCT
hCCR5_off-target5_Adaptor_R	GTGACTGGAGTTCAGACGTGTGCTCTTCCGATCT GAGAGGTGGAAAATGGAACC
hCCR5_off-target6_F	CATTTCCCCCAGCCTATACA
hCCR5_off-target6_R	GGCCAACATGGTGAAACTCT
hCCR5_off-target6_Adaptor_F	ACACTCTTTCCCTACACGACGCTCTTCCGATCT AACCATGAAGTGGCAAAACC
hCCR5_off-target6_Adaptor_R	GTGACTGGAGTTCAGACGTGTGCTCTTCCGATCT AAACCTTTGACCCTGCTCAT
hCCR5_off-target7_F	ACGAGGTCAGGAGTTCGAGA
hCCR5_off-target7_R	ATCCACGAGGGTTTTCTGTG

hCCR5_off-target7_Adaptor_F	ACACTCTTTCCCTACACGACGCTCTTCCGATCT TTTCCTCCTGGTCTTTTTCC
hCCR5_off-target7_Adaptor_R	GTGACTGGAGTTCAGACGTGTGCTCTTCCGATCT CCACTGCTTAGGATGGATGG
hCCR5_off-target8_F	TGGTGAAACCCCGTCTCTAC
hCCR5_off-target8_R	AACCTACTGGGCATGCTTTG
hCCR5_off-target8_Adaptor_F	ACACTCTTTCCCTACACGACGCTCTTCCGATCT GCTGAGGCAGGAGAATCACT
hCCR5_off-target8_Adaptor_R	GTGACTGGAGTTCAGACGTGTGCTCTTCCGATCTTGGTGACACTGAGCAGAATGA
hCCR5_off-target9_F	TGCACAAGGACAAAGCTCAC
hCCR5_off-target9_R	GGAGCTGACTGGCTCTCTGT
hCCR5_off-target9_Adaptor_F	ACACTCTTTCCCTACACGACGCTCTTCCGATCT ACAAGAAAGCAGGGACCTCA
hCCR5_off-target9_Adaptor_R	GTGACTGGAGTTCAGACGTGTGCTCTTCCGATCT AGCCTGGGTAAGCTGCACTA
hCCR5_off-target10_F	TTCCCAGCTACTGGCTCTTC
hCCR5_off-target10_R	TTGGGGCCAAGCTAGAGATA
hCCR5_off-target10_Adaptor_F	ACACTCTTTCCCTACACGACGCTCTTCCGATCT GTCAGCAGGAGAAGATATGG
hCCR5_off-target10_Adaptor_R	GTGACTGGAGTTCAGACGTGTGCTCTTCCGATCT AATGTCTTCATGTTACAGGG
hDNMT1_off-target1_F	CTGAGCTGGTATCCAAGATGC
hDNMT1_off-target1_R	GCATTGTCATTAGAACCACAAATC
hDNMT1_off-target1_Adaptor_F	ACACTCTTTCCCTACACGACGCTCTTCCGATCTGCAGAAGTGAGTCTTGCTGAG
hDNMT1_off-target1_Adaptor_R	GTGACTGGAGTTCAGACGTGTGCTCTTCCGATCTCAGAATCTGTGCACTCGGAG
hDNMT1_off-target2_F	ACAAAATTAGCTGGGCATGG
hDNMT1_off-target2_R	ATGTCTGCCAGGTACCCATC
hDNMT1_off-target2_Adaptor_F	ACACTCTTTCCCTACACGACGCTCTTCCGATCTTCAGTTGCATGACCCTGAGA
hDNMT1_off-target2_Adaptor_R	GTGACTGGAGTTCAGACGTGTGCTCTTCCGATCTTGTGGAAGTGAAGAATCCAATG
hDNMT1_off-target3_F	AAAGTGGAGACAGGGTGGTG
hDNMT1_off-target3_R	TTGTGCCTGTGTGAGGGATA
hDNMT1_off-target3_Adaptor_F	ACACTCTTTCCCTACACGACGCTCTTCCGATCT GGGGCAACAGTTTCATTGTC
hDNMT1_off-target3_Adaptor_R	GTGACTGGAGTTCAGACGTGTGCTCTTCCGATCT GGTATTTGGATAGGCCACGA
hDNMT1_off-target4_F	TAGCCCTTGCCTACTGAGGA
hDNMT1_off-target4_R	GGAGGGGAGAGAAACTGAGG
hDNMT1_off-target4_Adaptor_F	ACACTCTTTCCCTACACGACGCTCTTCCGATCT GGAGTTAAGGGTAGATCCTA
hDNMT1_off-target4_Adaptor_R	GTGACTGGAGTTCAGACGTGTGCTCTTCCGATCT GGGTTGGTGCCCTAAAAATC
hDNMT1_off-target5_F	AGAGGGGGCTAAAGGACAGA
hDNMT1_off-target5_R	TAGGGATGAGGACCAGGTTG
hDNMT1_off-target5_Adaptor_F	ACACTCTTTCCCTACACGACGCTCTTCCGATCT GCAGGGAGTGGATAGATGGA
hDNMT1_off-target5_Adaptor_R	GTGACTGGAGTTCAGACGTGTGCTCTTCCGATCT GGCTATGCAGATGAGGCACT
hDNMT1_off-target6_F	GAGACACCTGCCAGCTAAGG
hDNMT1_off-target6_R	ACAAGCTCACCCACTTCCAC
hDNMT1_off-target6_Adaptor_F	ACACTCTTTCCCTACACGACGCTCTTCCGATCT ACCACTGCCGTACAGAGGAG
hDNMT1_off-target6_Adaptor_R	GTGACTGGAGTTCAGACGTGTGCTCTTCCGATCT CAAGCTCACCCACTTCCACT
hDNMT1_off-target7_F	CCCATTTCTCCCAGATTCCT
hDNMT1_off-target7_R	CTCGGCTGCAAAGTCCTATC
hDNMT1_off-target7_Adaptor_F	ACACTCTTTCCCTACACGACGCTCTTCCGATCT CTCCCTAAAAGCCGCAAAC
hDNMT1_off-target7_Adaptor_R	GTGACTGGAGTTCAGACGTGTGCTCTTCCGATCT TTTCCAAACACCACCCTACA
hDNMT1_off-target8_F	CGGGCATTGTTCTACCACTT
hDNMT1_off-target8_R	AAGACCTGGCTACTGCGATG

hDNMT1_off-target8_Adaptor_F	ACACTCTTTCCCTACACGACGCTCTTCCGATCT CAAGAAAGGACGTGGGAAGA
hDNMT1_off-target8_Adaptor_R	GTGACTGGAGTTCAGACGTGTGCTCTTCCGATCT GGCACTTCAGACCCTGAATC
hDNMT1_off-target9_F	AGGGATTCTCACAGCCCTTT
hDNMT1_off-target9_R	CACCACCTTGCTGGATTTCT
hDNMT1_off-target9_Adaptor_F	ACACTCTTTCCCTACACGACGCTCTTCCGATCT GAGCGGAGCTTTGTCATCTC
hDNMT1_off-target9_Adaptor_R	GTGACTGGAGTTCAGACGTGTGCTCTTCCGATCT GGGCACCTAGGAGGAGAAAC
hDNMT1_off-target10_F	TTCCTGTGACCTCTGGTTCC
hDNMT1_off-target10_R	GCATGAGTAAAGGCCTGGAG
hDNMT1_off-target10_Adaptor_F	ACACTCTTTCCCTACACGACGCTCTTCCGATCT GAAGTTCAGCCCCTTCAGTG
hDNMT1_off-target10_Adaptor_R	GTGACTGGAGTTCAGACGTGTGCTCTTCCGATCT TTGGAGAACGGCTTCGTAAT
hPOLQ_on-target_F	TGTCACATGAAGTCCCCAAA
hPOLQ_on-target_R	AGGTCCCCGACACACTACAG
hPOLQ_on-target_Adaptor_F	ACACTCTTTCCCTACACGACGCTCTTCCGATCT GCTGTGATGTGCCTTATGGA
hPOLQ_on-target_Adaptor_R	GTGACTGGAGTTCAGACGTGTGCTCTTCCGATCT TTAGGTTCCTGCAAGCCTCT
hSIRPa_on-target_F	GAGGTACAGGGAGGGCATTT
hSIRPa_on-target_R	TGATGTGGCTCCTGTGAGAG
hSIRPa_on-target_Adaptor_F	ACACTCTTTCCCTACACGACGCTCTTCCGATCT GCTGGGGAGTGACATGATCT
hSIRPa_on-target_Adaptor_R	GTGACTGGAGTTCAGACGTGTGCTCTTCCGATCT TGCGAGAGGAAGGGAGTCTA
hPOLQ_off-target1_F	CCCTGCTTTTGCAGTCATTT
hPOLQ_off-target1_R	TGCTTCCAGCCAAGCTTTAT
hPOLQ_off-target1_Adaptor_F	ACACTCTTTCCCTACACGACGCTCTTCCGATCT AACTGCAAGAAGACAGTGATGG
hPOLQ_off-target1_Adaptor_R	GTGACTGGAGTTCAGACGTGTGCTCTTCCGATCT CCCCAGGTCTCATCTTCATC
hPOLQ_off-target2_F	CCGGCCTAGATGTTGTTGTT
hPOLQ_off-target2_R	CCACCATGCTTGGCTAATTT
hPOLQ_off-target2_Adaptor_F	ACACTCTTTCCCTACACGACGCTCTTCCGATCT TGCTCCTAAGTGCAAGAACG
hPOLQ_off-target2_Adaptor_R	GTGACTGGAGTTCAGACGTGTGCTCTTCCGATCTTTCCAACCAGCAGTACATGAAG
hPOLQ_off-target3_F	GAGACCAGGTTTTGCCATGT
hPOLQ_off-target3_R	GCCCATTCTCGTTATTTATGG
hPOLQ_off-target3_Adaptor_F	ACACTCTTTCCCTACACGACGCTCTTCCGATCT CCCAAGCACAGTACTGAAGTG
hPOLQ_off-target3_Adaptor_R	GTGACTGGAGTTCAGACGTGTGCTCTTCCGATCTCAACACTTTCATCAACCAATCAA
hPOLQ_off-target4_F	AGGTGACACTCTGCCGTCTT
hPOLQ_off-target4_R	CATGTAACGGGGTGTGTGTG
hPOLQ_off-target4_Adaptor_F	ACACTCTTTCCCTACACGACGCTCTTCCGATCT GGCTGTGATGTGCCTTACTG
hPOLQ_off-target4_Adaptor_R	GTGACTGGAGTTCAGACGTGTGCTCTTCCGATCT GATCTCGGCTCACTGCAAG
hPOLQ_off-target5_F	TTTTCCAGGACTGGGTTGAG
hPOLQ_off-target5_R	GGAACGTCAGTAAGCCACCA
hPOLQ_off-target5_Adaptor_F	ACACTCTTTCCCTACACGACGCTCTTCCGATCT GCAGGCCTCTGGTCACATAG
hPOLQ_off-target5_Adaptor_R	GTGACTGGAGTTCAGACGTGTGCTCTTCCGATCT AAAATGGTCCCCAAACACTG
hPOLQ_off-target6_F	GAGGACAAAGCCAAAAGCTG
hPOLQ_off-target6_R	GAATGGATGTGCCAGGAAGT
hPOLQ_off-target6_Adaptor_F	ACACTCTTTCCCTACACGACGCTCTTCCGATCT CCCCTGGACACAACATTTTC
hPOLQ_off-target6_Adaptor_R	GTGACTGGAGTTCAGACGTGTGCTCTTCCGATCT TGTGATGTGCCTTATGGAGAA
hPOLQ_off-target7_F	TTAGACCAGTGGCACCCATT

hPOLQ_off-target7_R	AAACCTTGAACCCTGGGAGT
hPOLQ_off-target7_Adaptor_F	ACACTCTTTCCCTACACGACGCTCTTCCGATCT TGCAGATGATATTGCCCTTC
hPOLQ_off-target7_Adaptor_R	GTGACTGGAGTTCAGACGTGTGCTCTTCCGATCT CCCTGGTTCAAAGCCATGTA
hPOLQ_off-target8_F	GAGTCAGCCCCTCAATTCTG
hPOLQ_off-target8_R	ATACAAGCACTGGGCCCTAA
hPOLQ_off-target8_Adaptor_F	ACACTCTTTCCCTACACGACGCTCTTCCGATCT TGCGCCATTACAAGAACATC
hPOLQ_off-target8_Adaptor_R	GTGACTGGAGTTCAGACGTGTGCTCTTCCGATCT CTGGACTATGAGGCCAGCAC
hPOLQ_off-target9_F	CTTTGGGCAACCAGGTAAAA
hPOLQ_off-target9_R	TTAAGGATGTGCGCAGAGTG
hPOLQ_off-target9_Adaptor_F	ACACTCTTTCCCTACACGACGCTCTTCCGATCT CTGCAAGCCAATGGTCATAA
hPOLQ_off-target9_Adaptor_R	GTGACTGGAGTTCAGACGTGTGCTCTTCCGATCT GGCTATAATGTGCCTTCTGGA
hPOLQ_off-target10_F	CAACTGTGCTGACTCCCCTA
hPOLQ_off-target10_R	GGACACAGGCGCAGATAAAT
hPOLQ_off-target10_Adaptor_F	ACACTCTTTCCCTACACGACGCTCTTCCGATCT ACCCAGTTGGGATAAATTGC
hPOLQ_off-target10_Adaptor_R	GTGACTGGAGTTCAGACGTGTGCTCTTCCGATCT CAGAAAAGGGAAGAGGGAGA
hSIRPa_off-target1_F	CATCTCCTGGGTTCAAGCAT
hSIRPa_off-target1_R	ACCACACCTGGCCCATATTA
hSIRPa_off-target1_Adaptor_F	ACACTCTTTCCCTACACGACGCTCTTCCGATCT CCCGGCCCATATTGACTTTA
hSIRPa_off-target1_Adaptor_R	GTGACTGGAGTTCAGACGTGTGCTCTTCCGATCT TCTGGCATTCAGATTCTGTC
hSIRPa_off-target2_F	TTTGCTCCCCGTCTTAGGTA
hSIRPa_off-target2_R	CCAATGCCTGGCTAATTGTT
hSIRPa_off-target2_Adaptor_F	ACACTCTTTCCCTACACGACGCTCTTCCGATCT AACCAAATGCTGGTTCTTCG
hSIRPa_off-target2_Adaptor_R	GTGACTGGAGTTCAGACGTGTGCTCTTCCGATCT TTGAACTCCTGGGCTCAAGT
hSIRPa_off-target3_F	CTGTGGGCCCTTGTGTTACT
hSIRPa_off-target3_R	TCTAGCCTGGGCAACAGAGT
hSIRPa_off-target3_Adaptor_F	ACACTCTTTCCCTACACGACGCTCTTCCGATCT TCTCCTGACCTCATGATCCA
hSIRPa_off-target3_Adaptor_R	GTGACTGGAGTTCAGACGTGTGCTCTTCCGATCT TGGGGAAGAGAGAAAAGGTG
hSIRPa_off-target4_F	GGCTTCAAGGCCTCTTTTCT
hSIRPa_off-target4_R	TGCAGCTCTTTGGAACTGAC
hSIRPa_off-target4_Adaptor_F	ACACTCTTTCCCTACACGACGCTCTTCCGATCT GAAAAGGGGGCTGTGTGTAA
hSIRPa_off-target4_Adaptor_R	GTGACTGGAGTTCAGACGTGTGCTCTTCCGATCT AAGGCAAAATCAGCAGCTTG
hSIRPa_off-target5_F	AGGCAGGTGGATTGTTTGAG
hSIRPa_off-target5_R	CAATGAAGCACCATGGTTTG
hSIRPa_off-target5_Adaptor_F	ACACTCTTTCCCTACACGACGCTCTTCCGATCT AGCTACTGAGGAGGCTGAGG
hSIRPa_off-target5_Adaptor_R	GTGACTGGAGTTCAGACGTGTGCTCTTCCGATCT GCCACGCACAAAGGATATTT
hSIRPa_off-target6_F	CCTGAACCACTGCCTTTTTC
hSIRPa_off-target6_R	AGAGGGGAGAGCAAGAGAGG
hSIRPa_off-target6_Adaptor_F	ACACTCTTTCCCTACACGACGCTCTTCCGATCT GCAAACTCCATAGAACTCCAC
hSIRPa_off-target6_Adaptor_R	GTGACTGGAGTTCAGACGTGTGCTCTTCCGATCT TTTCATCAAAAGGGTGTCCA
hSIRPa_off-target7_F	TCTCACCTAGCCCTGGAAGA
hSIRPa_off-target7_R	CACCCAGCCTTCATTCTTGT
hSIRPa_off-target7_Adaptor_F	ACACTCTTTCCCTACACGACGCTCTTCCGATCT CAAAAGGCATAAGGACAGCC
hSIRPa_off-target7_Adaptor_R	GTGACTGGAGTTCAGACGTGTGCTCTTCCGATCT GGAATCAGCGGCACAACTAA
hSIRPa_off-target8_F	GATCTTCAGGCTGTCCTTGG
hSIRPa_off-target8_R	GTGCCAGCCTTTTCAGACTC

hSIRPa_off-target8_Adaptor_F	ACACTCTTTCCCTACACGACGCTCTTCCGATCT GGCTTTTACCCCATACACCA
hSIRPa_off-target8_Adaptor_R	GTGACTGGAGTTCAGACGTGTGCTCTTCCGATCT TCATGACCTGACCTGAAGCA
hSIRPa_off-target9_F	TTGGAGAGCACACTTCGTTG
hSIRPa_off-target9_R	AGCTGCCATTAACCCAGATG
hSIRPa_off-target9_Adaptor_F	ACACTCTTTCCCTACACGACGCTCTTCCGATCT AACAACCCTGTGTGGTAGGC
hSIRPa_off-target9_Adaptor_R	GTGACTGGAGTTCAGACGTGTGCTCTTCCGATCT TAGCTGCATTTGCCAATCTT
hSIRPa_off-target10_F	GGCAGGAGAATCATTTGAGG
hSIRPa_off-target10_R	TGTGATGGAGTCTCGCTCTG
hSIRPa_off-target10_Adaptor_F	ACACTCTTTCCCTACACGACGCTCTTCCGATCT CAGTCTGGGTGACAAAGCAA
hSIRPa_off-target10_Adaptor_R	GTGACTGGAGTTCAGACGTGTGCTCTTCCGATCT TAACAGGCATGAGCCATCAC

Supplementary Figures

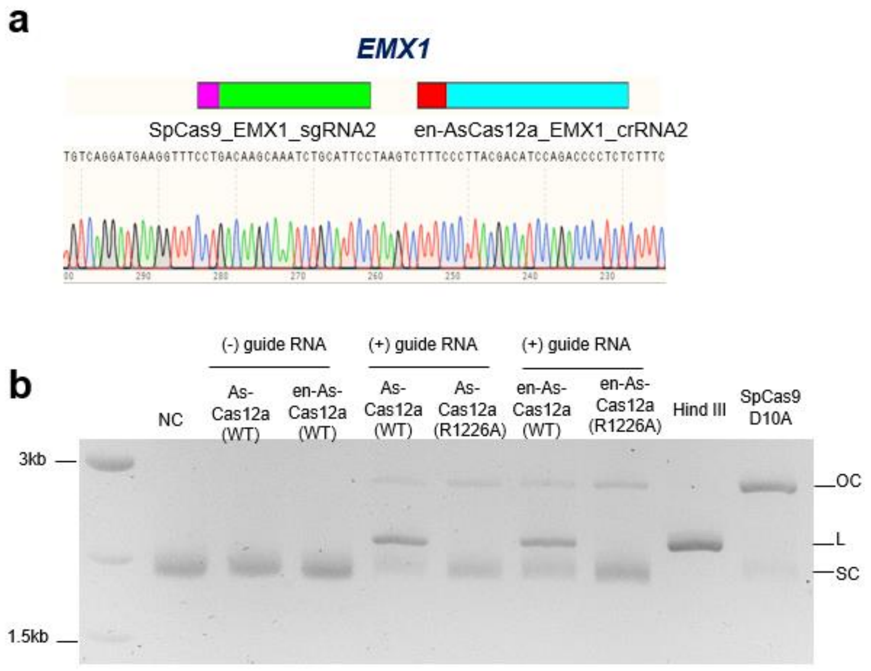

c

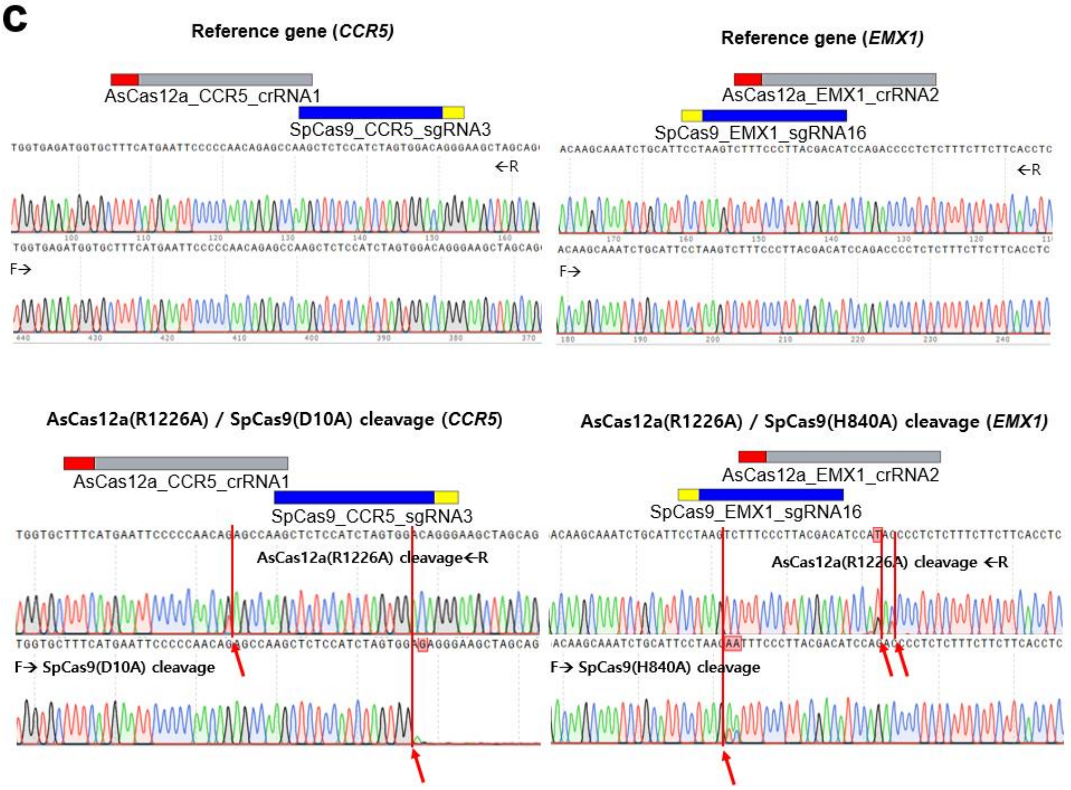
Reference gene (CCR5)
AsCas12a_CCR5_crRNA1
SpCas9_CCR5_sgRNA3
←R
F→
Reference gene (EMX1)
AsCas12a_EMX1_crRNA2
SpCas9_EMX1_sgRNA16
←R
F→
AsCas12a(R1226A) / SpCas9(D10A) cleavage (CCR5)
AsCas12a_CCR5_crRNA1
SpCas9_CCR5_sgRNA3
AsCas12a(R1226A) cleavage←R
F→ SpCas9(D10A) cleavage
AsCas12a(R1226A) / SpCas9(H840A) cleavage (EMX1)
AsCas12a_EMX1_crRNA2
SpCas9_EMX1_sgRNA16
AsCas12a(R1226A) cleavage ←R
F→ SpCas9(H840A) cleavage

d

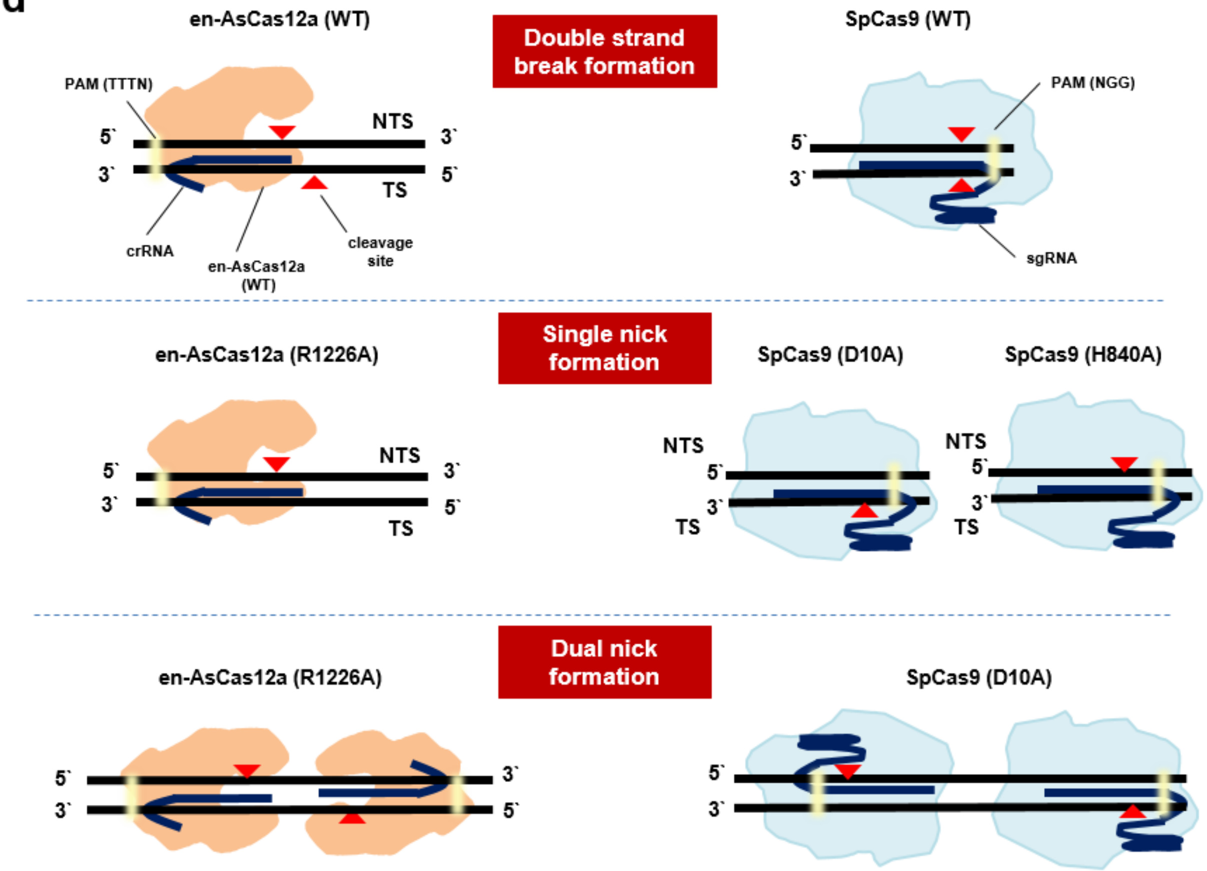
en-AsCas12a (WT)
Double strand break formation
SpCas9 (WT)
PAM (TTTN)
NTS
TS
crRNA
en-AsCas12a (WT)
cleavage site
PAM (NGG)
sgRNA
en-AsCas12a (R1226A)
Single nick formation
SpCas9 (D10A)
SpCas9 (H840A)
NTS
TS
en-AsCas12a (R1226A)
Dual nick formation
SpCas9 (D10A)

Supplementary Figure 1. Analysis of nicking properties of en-AsCas12a nickase (R1226A) by *in-vitro* cleavage experiment. (a) Sanger sequencing data of the plasmid containing the target nucleotide sequence (*EMX1*). PAM and targeted sequences are shown in cyon (SpCas9) and yellow (AsCas12a) and dark blue (SpCas9) and gray (AsCas12a), respectively. (b) Plasmid cleavage assay using recombinant proteins (en-AsCas12a, en-AsCas12a (R1226A), SpCas9 (D10A)). HindIII: restriction enzyme, OC: open circular form, L: linear form, SC: super-coiled form. –Guide RNA: only en-AsCas12a or en-AsCas12a (R1226A) protein treated, +Guide RNA: en-AsCas12a or en-AsCas12a (R1226A) protein and target-specific crRNA treated. (c) Identification of the cleavage site of en-AsCas12a nicksae (R1226A) using run-off sequencing. Sequencing results were compared to reference after cleavage using en-AsCas12a nicksae (R1226A) and Cas9 nickase (D10A or H840A) for the *CCR5* and *EMX1* genes, respectively. PAM (TTTN), protospacer sequence for AsCas12a and PAM (NGG), protospacer for SpCas9 is indicated by red, gray, yellow and blue, respectively. F: Sanger sequencing results with primers in the forward direction, R: Sanger sequencing results with primers in the reverse direction, Red arrow: A-tailing formed by polymerase run-off at the DNA nicking site. (d) Schematic of DNA cleavage patterns induced by en-AsCas12a / SpCas9 effector. Left: Schematics of the DNA cleavage by wild-type en-AsCas12a effector (Top), single en-AsCas12a (R1226A) nickase (middle), and dual en-AsCas12a (R1226A) nickase (bottom). Right: Schematics of the DNA cleavage by wild-type SpCas9 effector (Top), single SpCas9 (D10A or H840A) nickase (middle), and dual SpCas9 (D10A) nickase (bottom). Red arrows indicate cleavage induced on DNA. Yellow box indicates PAM (TTTN) sequence for AsCas12a and PAM (NGG) for SpCas9, respectively. NTS: Non-target strand, TS: Target strand.

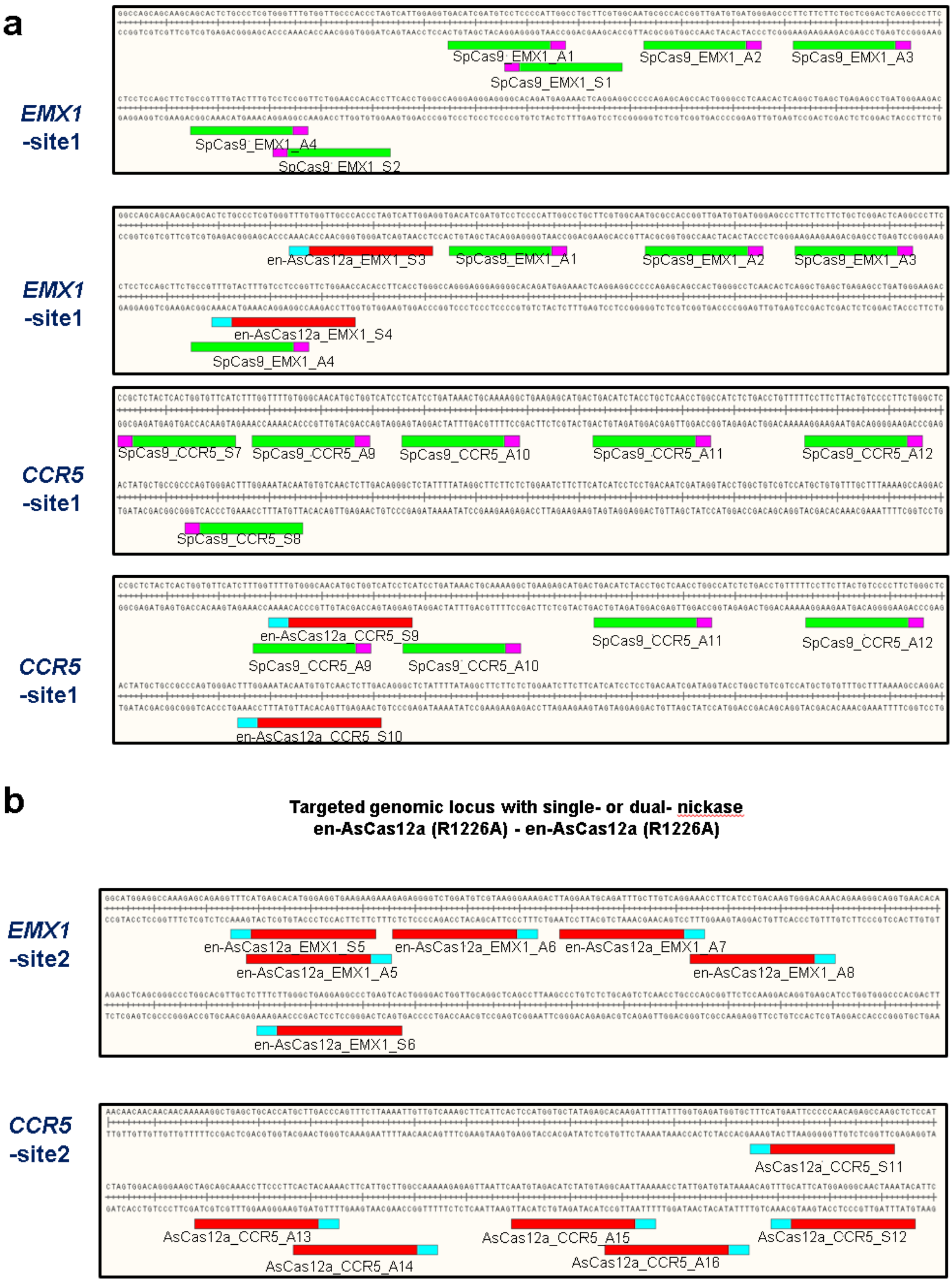
a
EMX1 -site1
SpCas9_EMX1_A1
SpCas9_EMX1_A2
SpCas9_EMX1_A3
SpCas9_EMX1_S1
SpCas9_EMX1_A4
SpCas9_EMX1_S2
EMX1 -site1
en-AsCas12a_EMX1_S3
SpCas9_EMX1_A1
SpCas9_EMX1_A2
SpCas9_EMX1_A3
en-AsCas12a_EMX1_S4
SpCas9_EMX1_A4
CCR5 -site1
SpCas9_CCR5_S7
SpCas9_CCR5_A9
SpCas9_CCR5_A10
SpCas9_CCR5_A11
SpCas9_CCR5_A12
SpCas9_CCR5_S8
CCR5 -site1
en-AsCas12a_CCR5_S9
SpCas9_CCR5_A11
SpCas9_CCR5_A12
SpCas9_CCR5_A9
SpCas9_CCR5_A10
en-AsCas12a_CCR5_S10
b
Targeted genomic locus with single- or dual- nickase
en-AsCas12a (R1226A) - en-AsCas12a (R1226A)
EMX1 -site2
en-AsCas12a_EMX1_S5
en-AsCas12a_EMX1_A6
en-AsCas12a_EMX1_A7
en-AsCas12a_EMX1_A5
en-AsCas12a_EMX1_A8
en-AsCas12a_EMX1_S6
CCR5 -site2
AsCas12a_CCR5_S11
AsCas12a_CCR5_A13
AsCas12a_CCR5_A15
AsCas12a_CCR5_S12
AsCas12a_CCR5_A14
AsCas12a_CCR5_A16

C

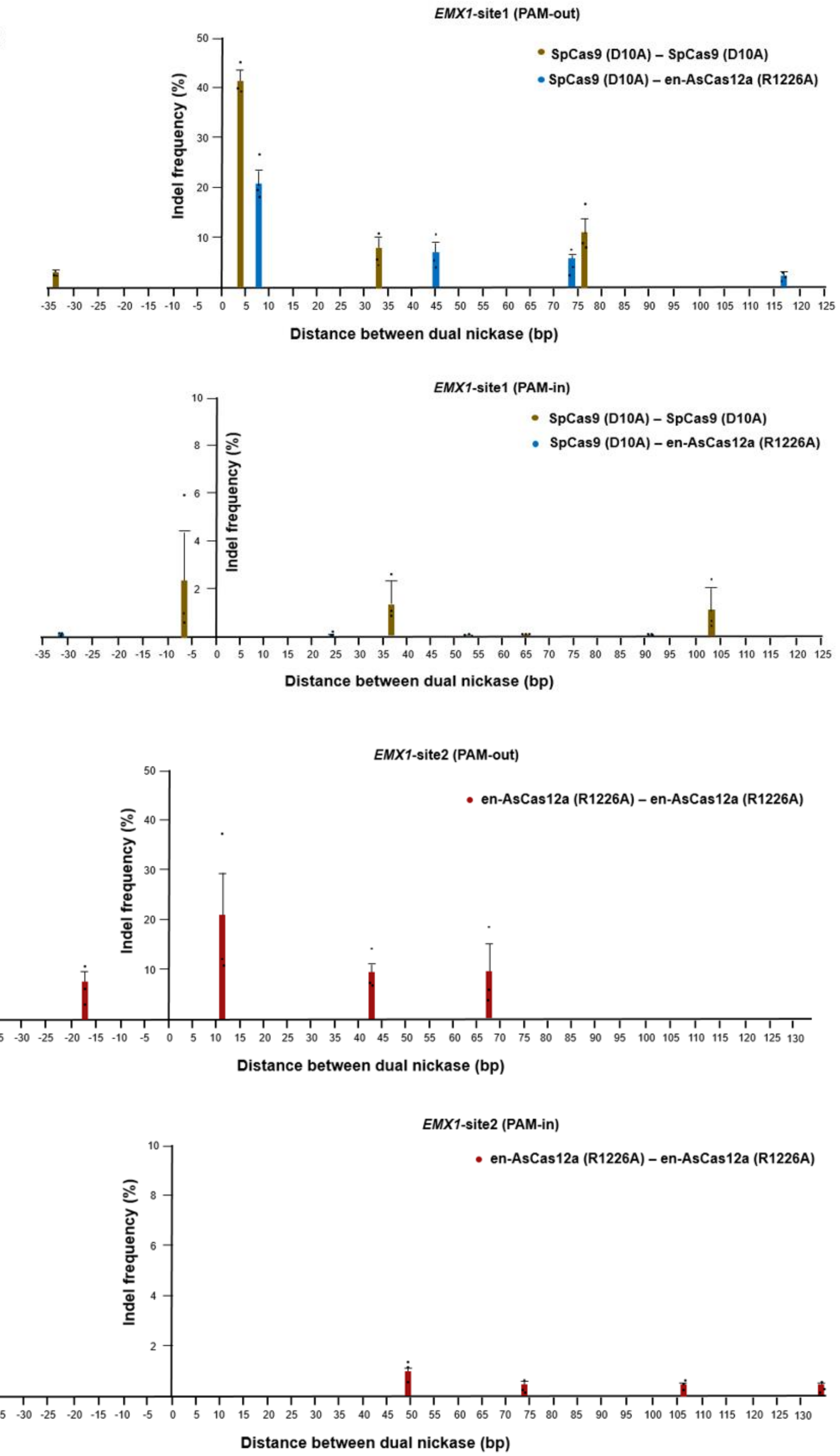

EMX1-site1 (PAM-out)
SpCas9 (D10A) – SpCas9 (D10A)
SpCas9 (D10A) – en-AsCas12a (R1226A)
Indel frequency (%)
Distance between dual nickase (bp)
EMX1-site1 (PAM-in)
SpCas9 (D10A) – SpCas9 (D10A)
SpCas9 (D10A) – en-AsCas12a (R1226A)
Indel frequency (%)
Distance between dual nickase (bp)
EMX1-site2 (PAM-out)
en-AsCas12a (R1226A) – en-AsCas12a (R1226A)
Indel frequency (%)
Distance between dual nickase (bp)
EMX1-site2 (PAM-in)
en-AsCas12a (R1226A) – en-AsCas12a (R1226A)
Indel frequency (%)
Distance between dual nickase (bp)

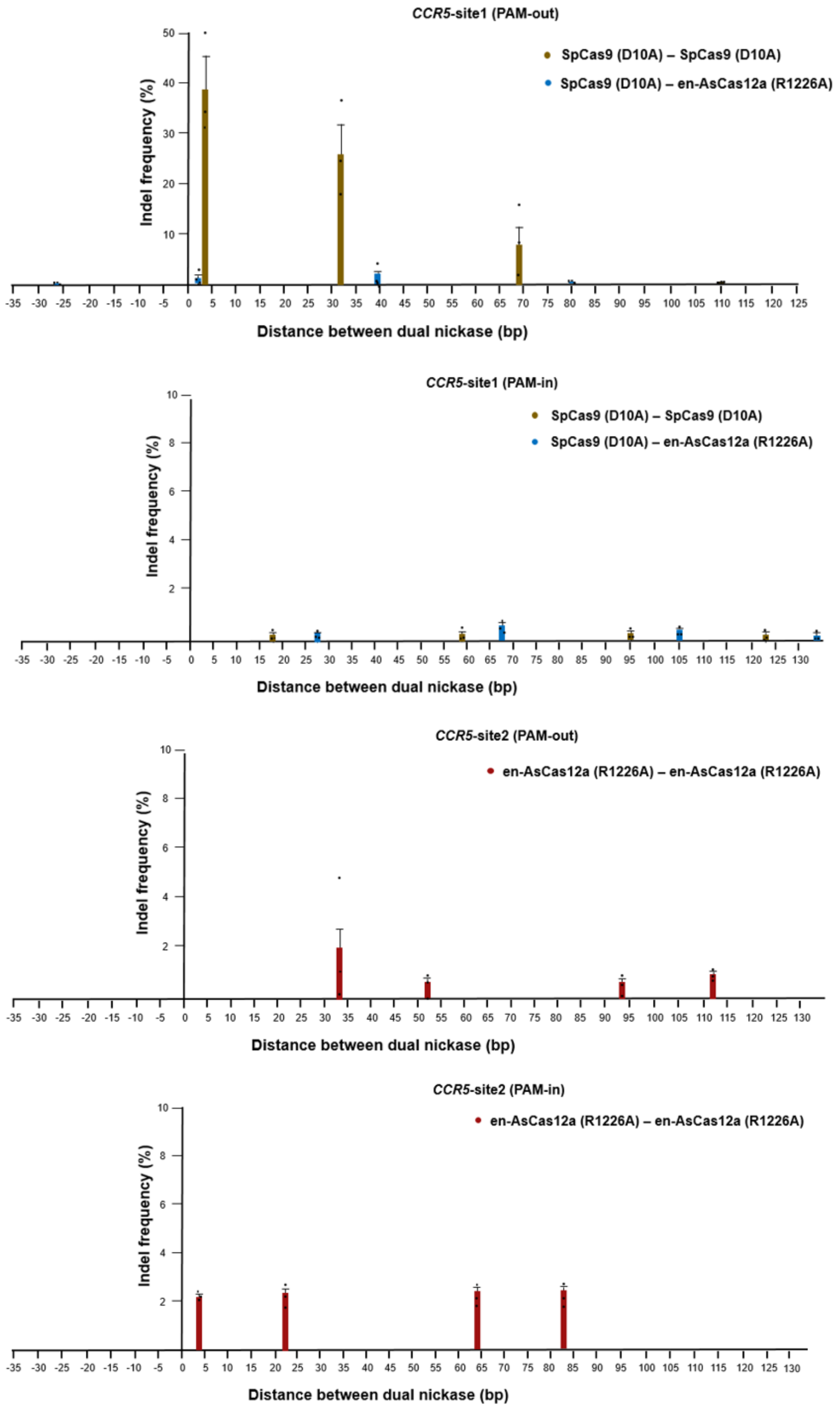

Supplementary Figure 2. Schematics of the targeting positions on genomic DNA for SpCas9 (D10A) or en-AsCas12a (R1226A) nickases. (a, b) PAM and protospacer

sequences recognized by SpCas9 (D10A) nickase are shown in magenta and pale green, respectively. The PAM and protospacer sequences recognized by en-AsCas12a (R1226A) nickase are shown in cyon and red, respectively. (c) Indel frequency (%) induced by dual en-AsCas12a (R1226A) and SpCas9 (D10A) nickase as a function of the distance between the actual nickase target sequences. Negative value in X-axis indicates the overlap between nickases. Each histogram was plotted by applying standard error of the mean values to three repeated experimental values (n = 3).

Figure 2a, 3d

Endogenous locus_indel patterns of *EMX1* -site1

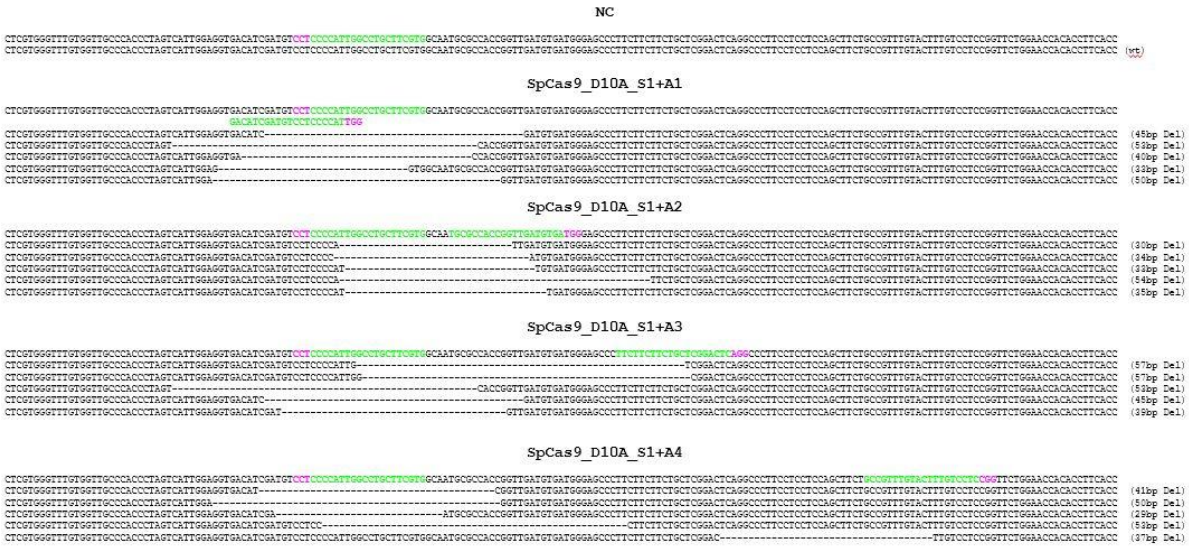

Figure 2b, 3d

Endogenous locus_indel patterns of *EMX1* site1

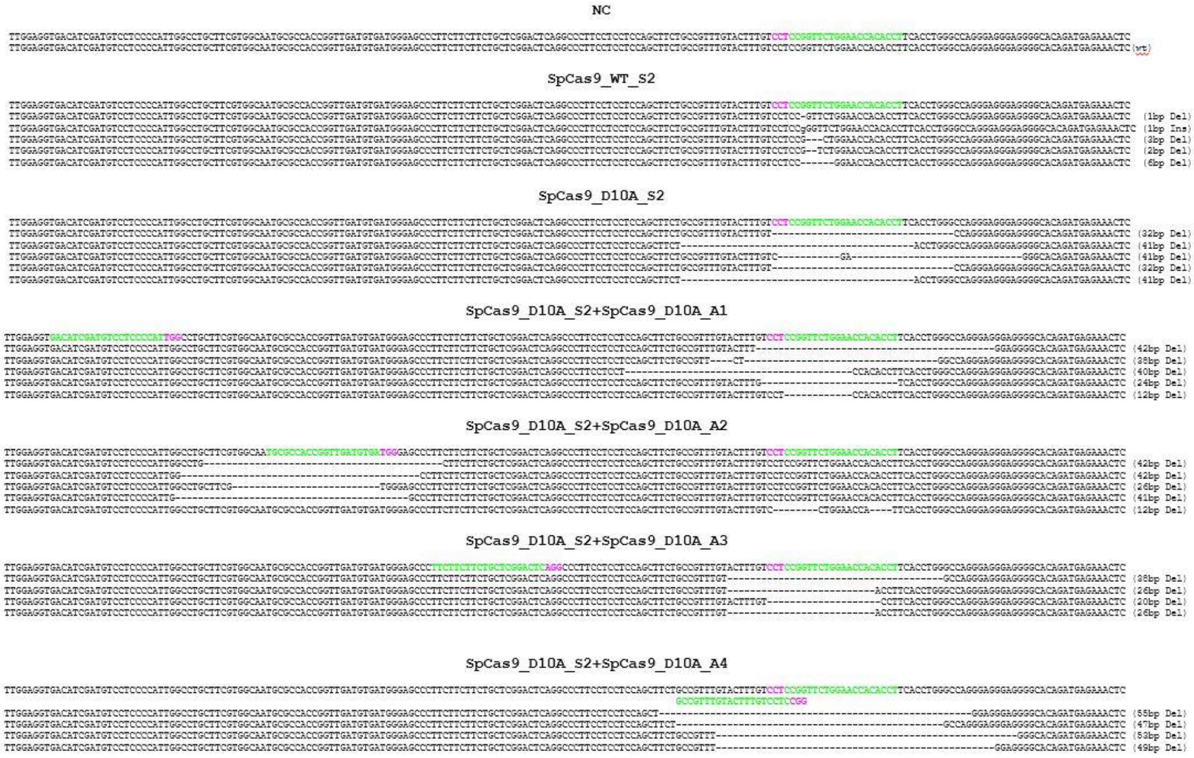

Figure 2c, 3e

Endogenous locus_indel patterns of *EMX1* site1

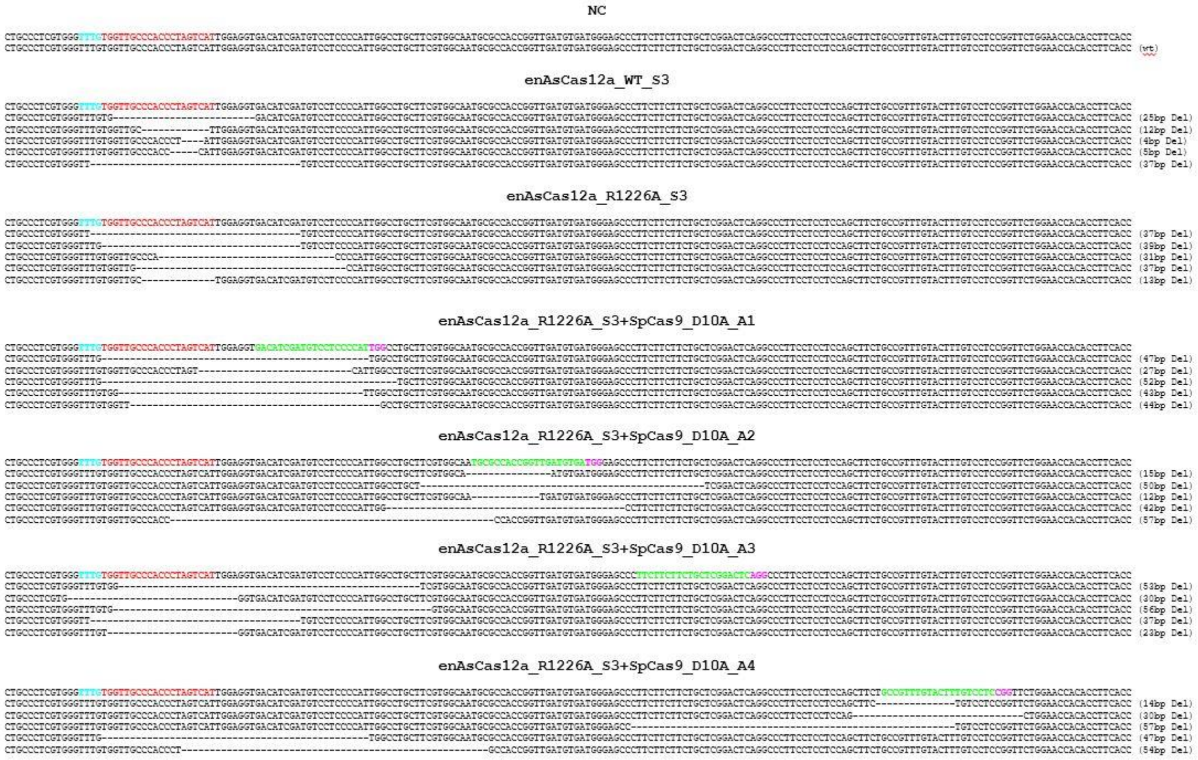

Figure 2c, 3e

Endogenous locus_indel patterns of *EMX1* site1

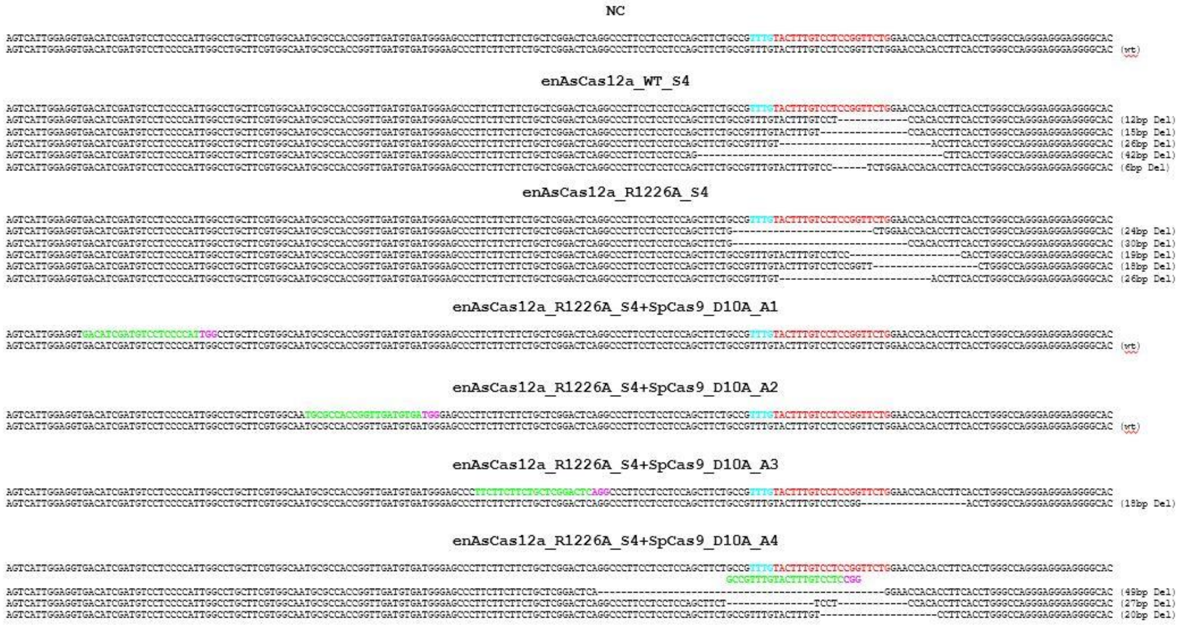

Figure 2e, 3f

Endogenous locus_indel patterns of *EMX1* site2

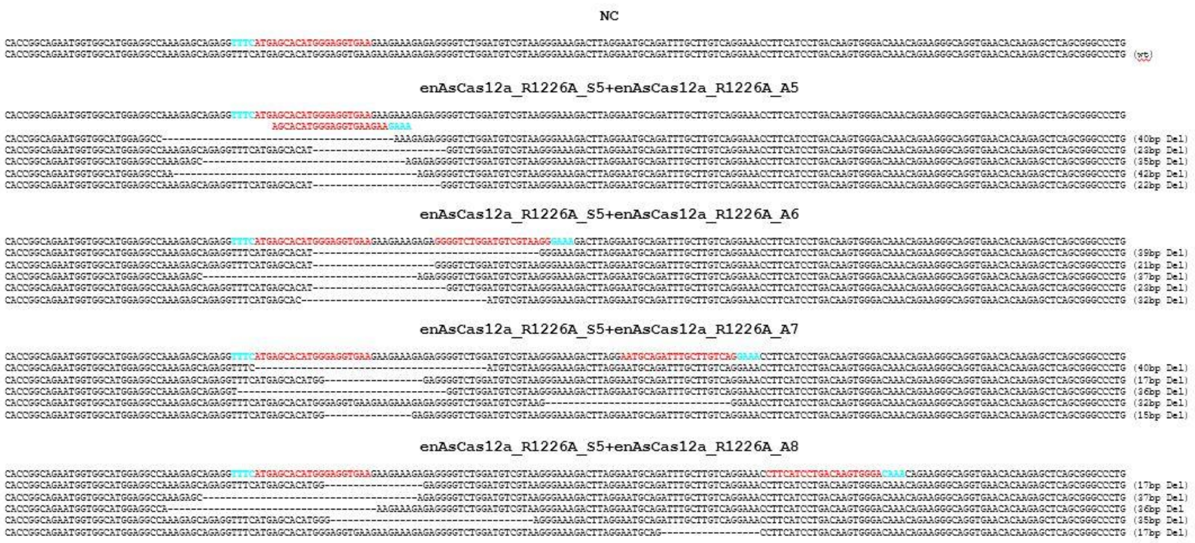

Figure 2e, 3f

Endogenous locus_indel patterns of *EMX1* site2

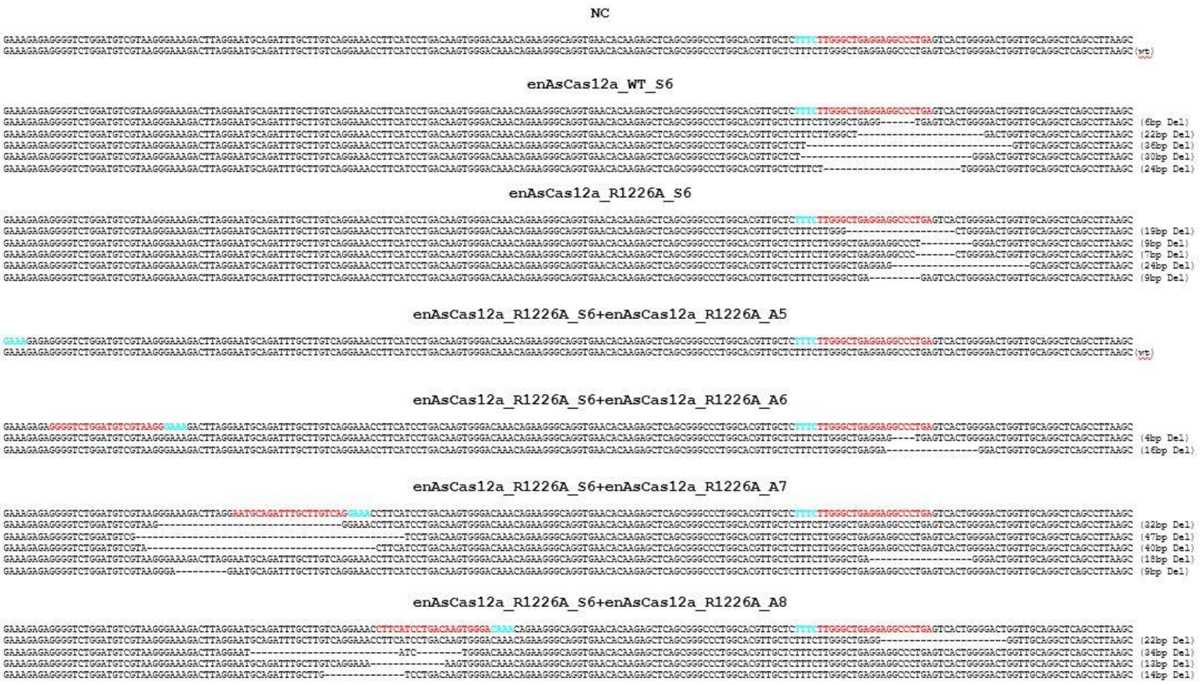

Figure 2a, 3j

Endogenous locus_indel patterns of *CCR5* site1

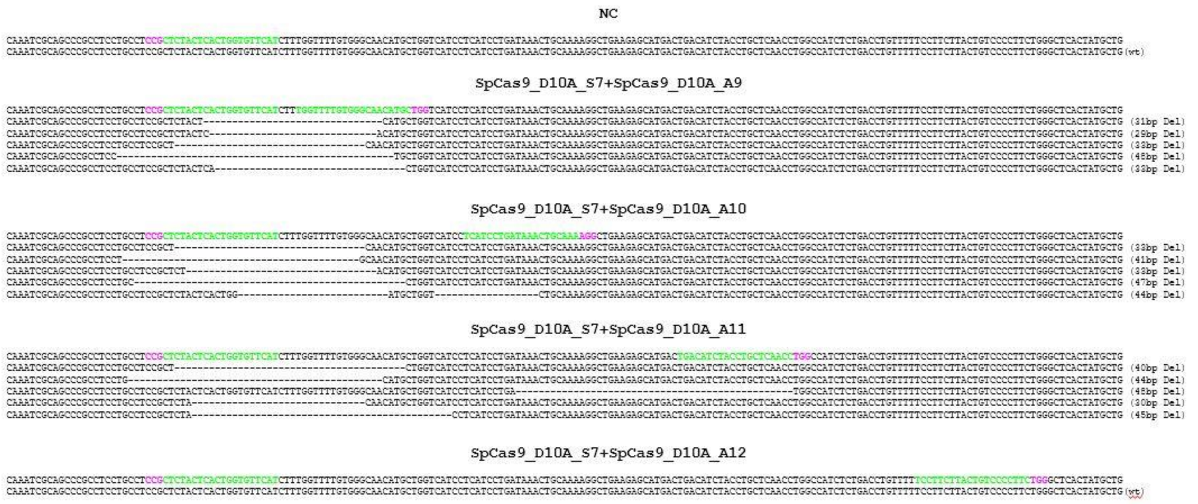

Figure 2b, 3j

Endogenous locus_indel patterns of *CCR5* site1

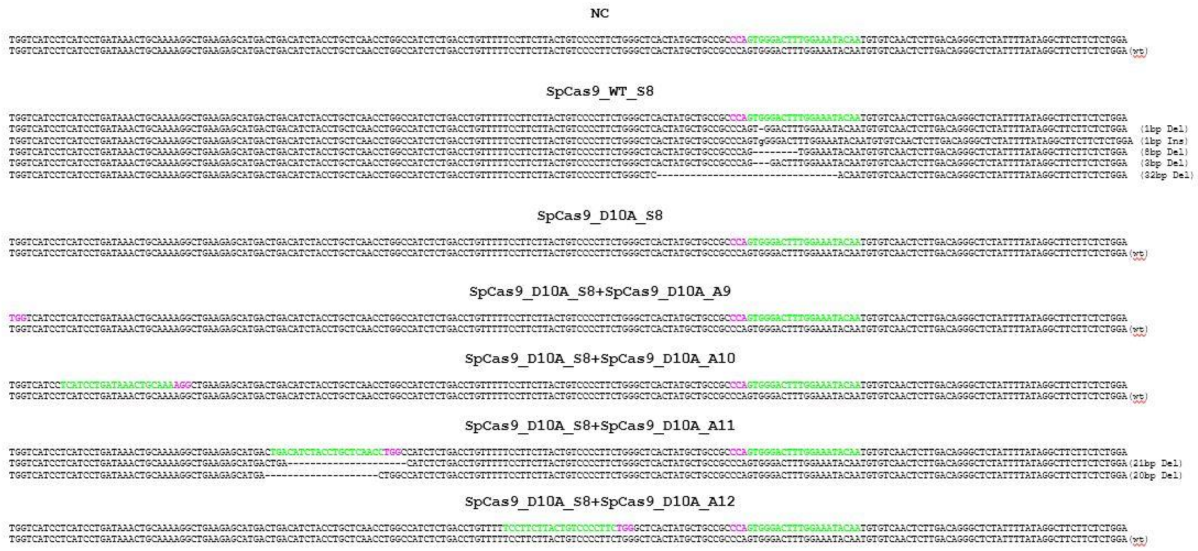

Figure 2c, 3k

Endogenous locus_indel patterns of *CCR5* site1

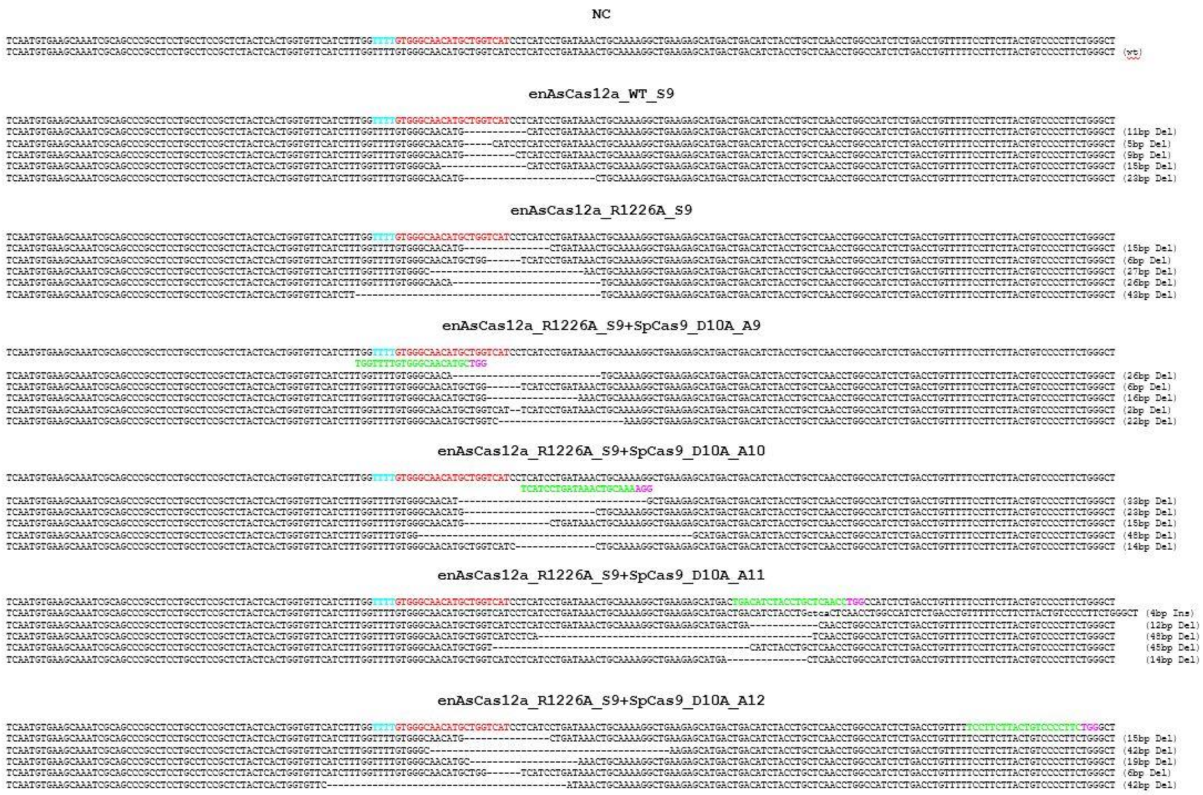

Figure 2d, 3k

Endogenous locus_indel patterns of *CCR5* site1

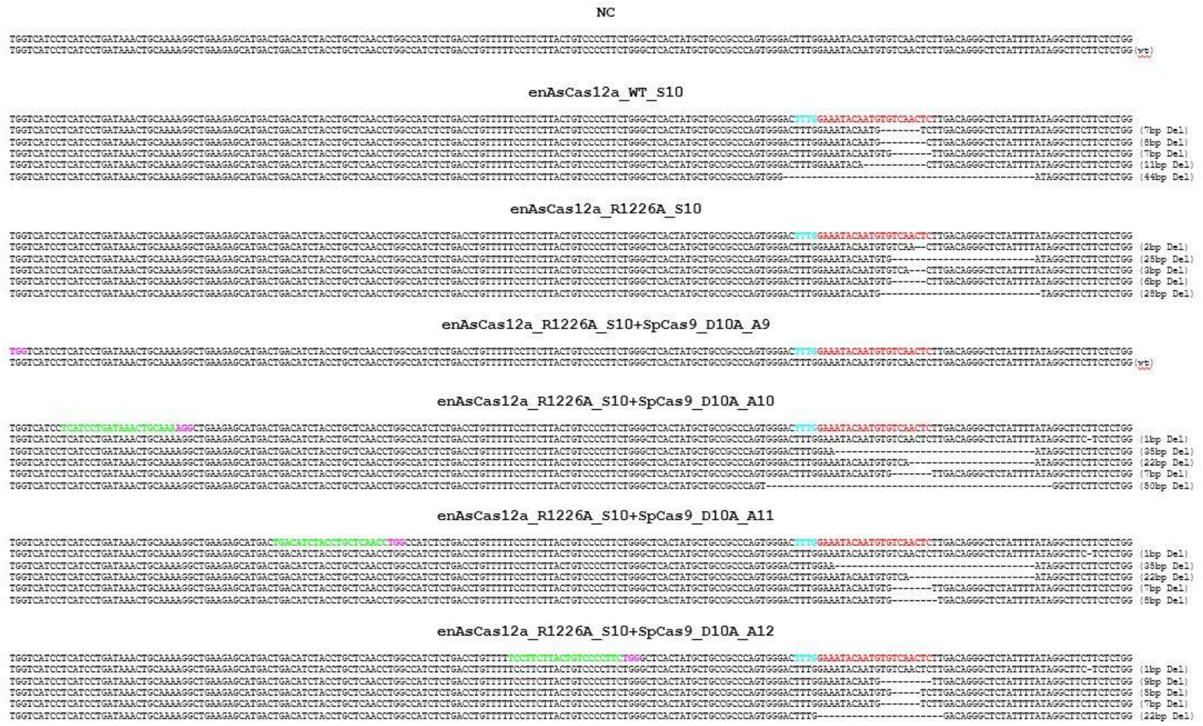

Figure 2e, 3l

Endogenous locus_indel patterns of *CCR5* site2

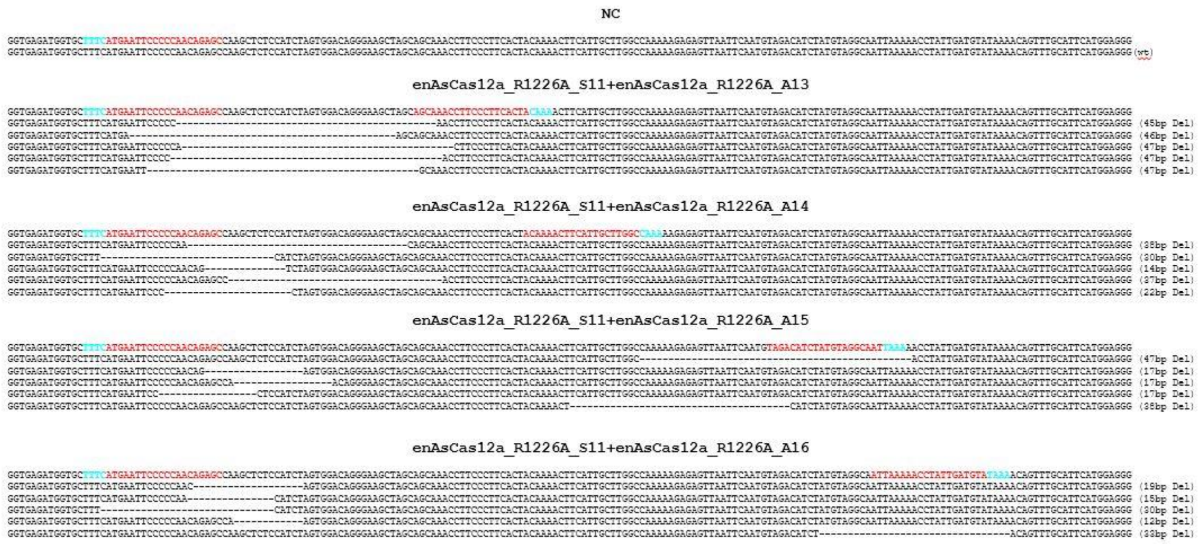

Figure 2f, 3l

Endogenous locus_indel patterns of *CCR5* site2

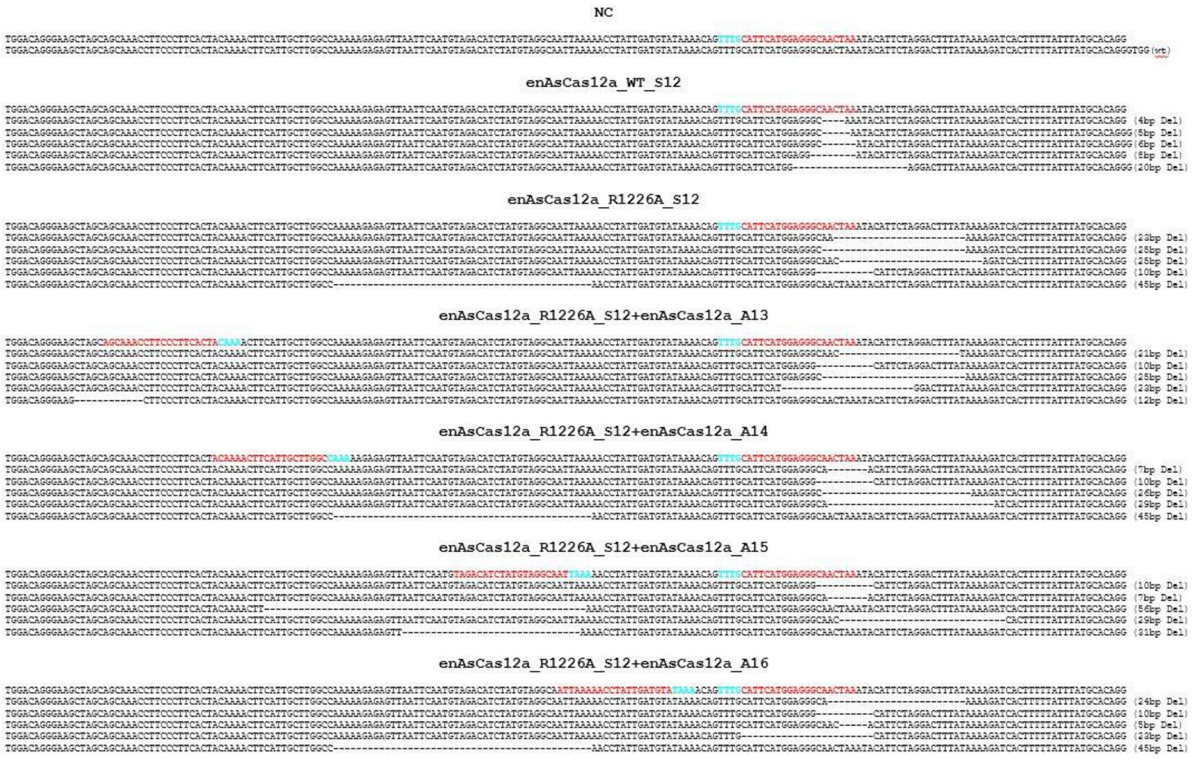

Supplementary Figure 3. Representative indel patterns induced by SpCas9 (D10A) or en-AsCas12a (R1226A) nickases on endogenous genes (*EMX1, CCR5*). In main text figures 2 and 3, each corresponding representative indel form is indicated in the upper left corner of the sequencing result. PAM and protospacer sequences

recognized by SpCas9 (D10A) nickase are shown in magenta and pale green, respectively. The PAM and protospacer sequences recognized by en-AsCas12a (R1226A) nickase are shown in cyon and red, respectively. Deleted sequences are indicated by dotted lines.

Figure 3d

Endogenous locus_indel patterns of *EMX1* site1

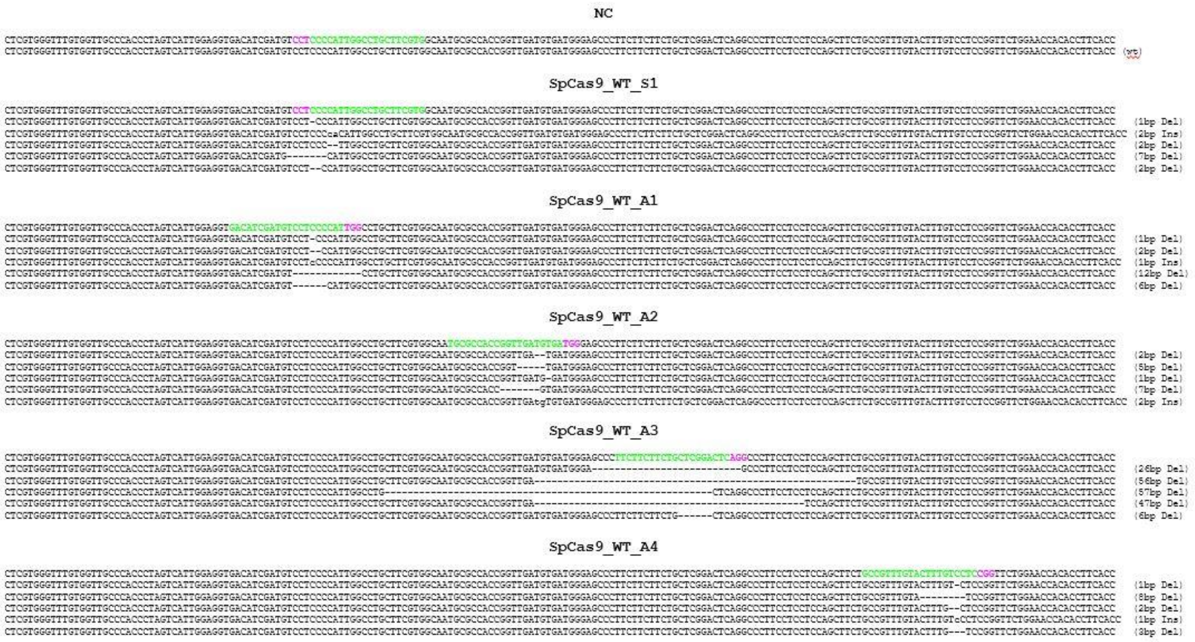

Figure 3d

Endogenous locus_indel patterns of *EMX1* site1

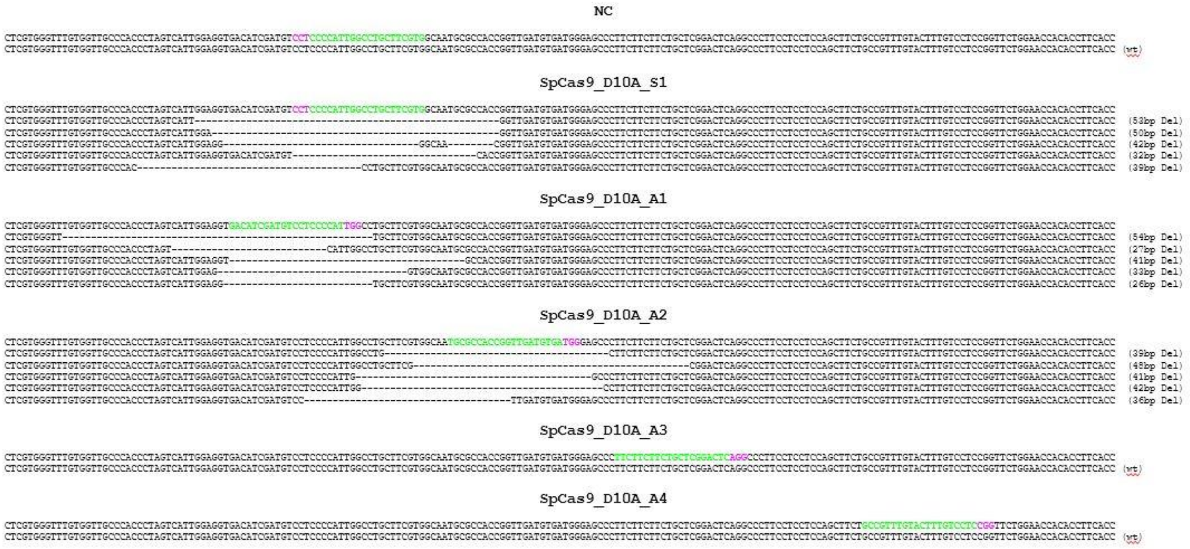

Figure 3f

Endogenous locus_indel patterns of *EMX1* site2

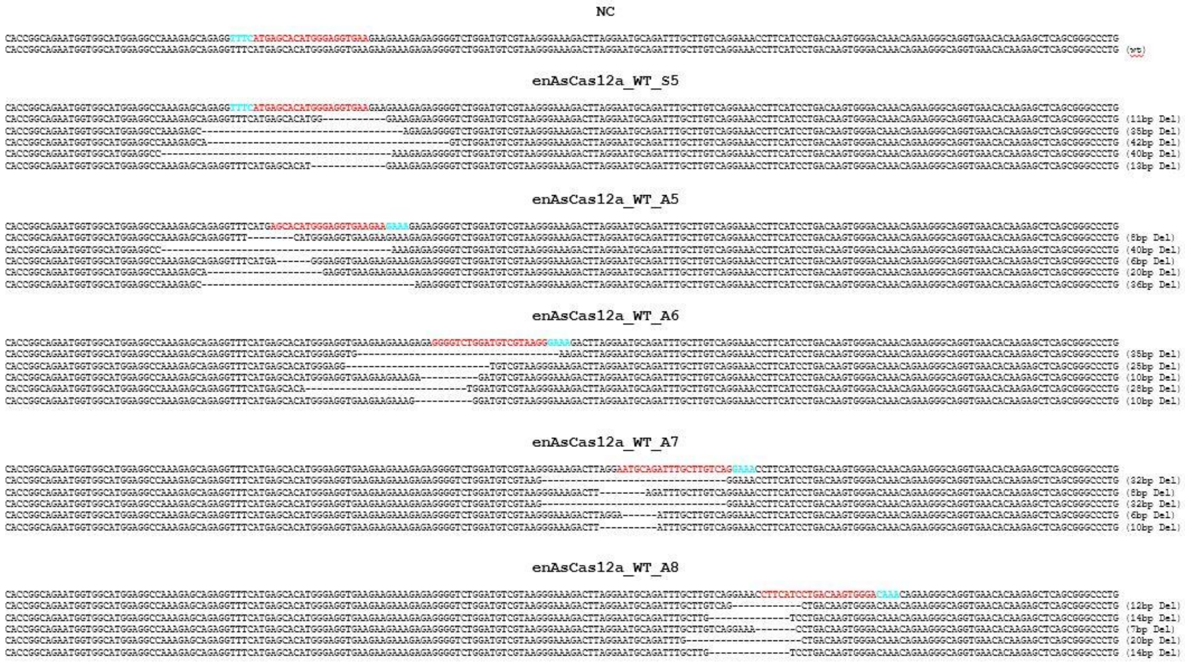

Figure 3f

Endogenous locus_indel patterns of *EMX1* site2

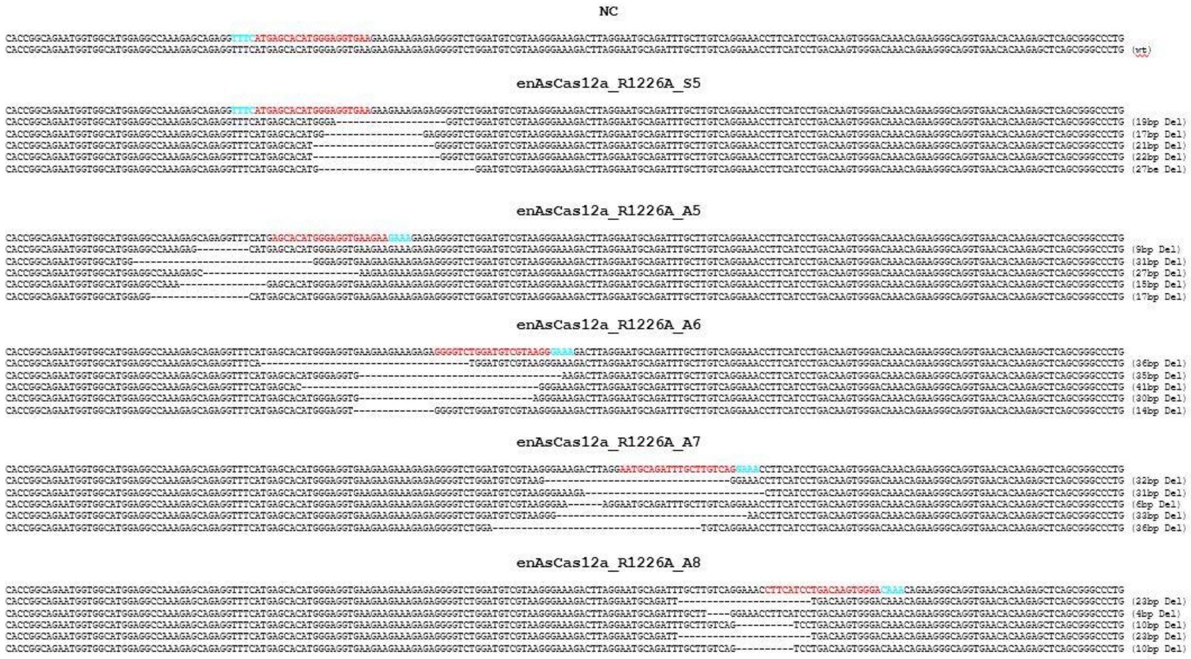

Figure 3j

Endogenous locus_indel patterns of *CCR5* site1

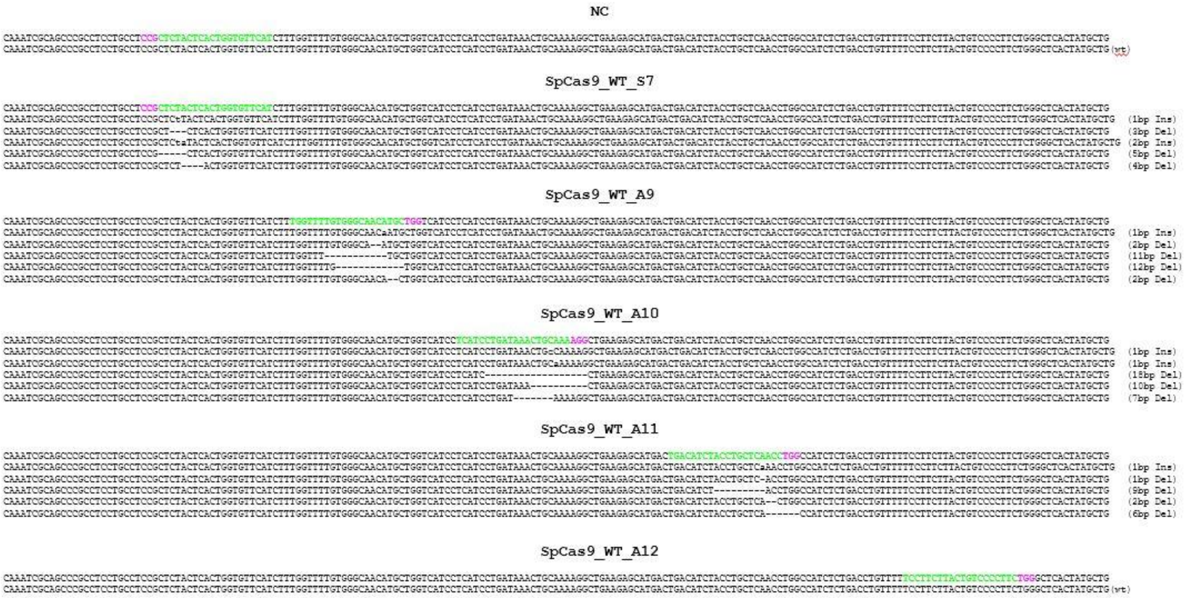

Figure 3j

Endogenous locus_indel patterns of *CCR5* site1

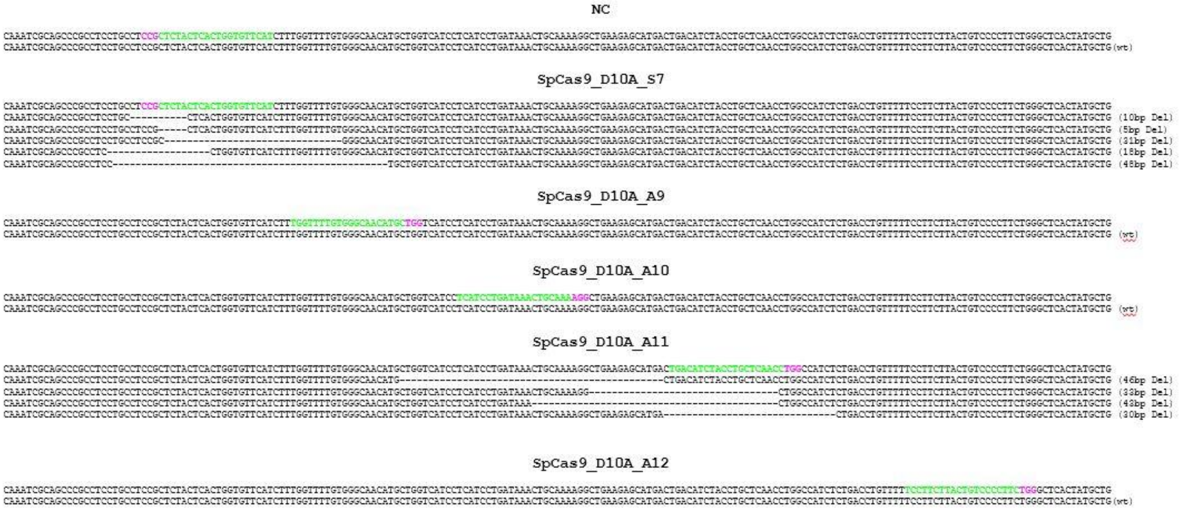

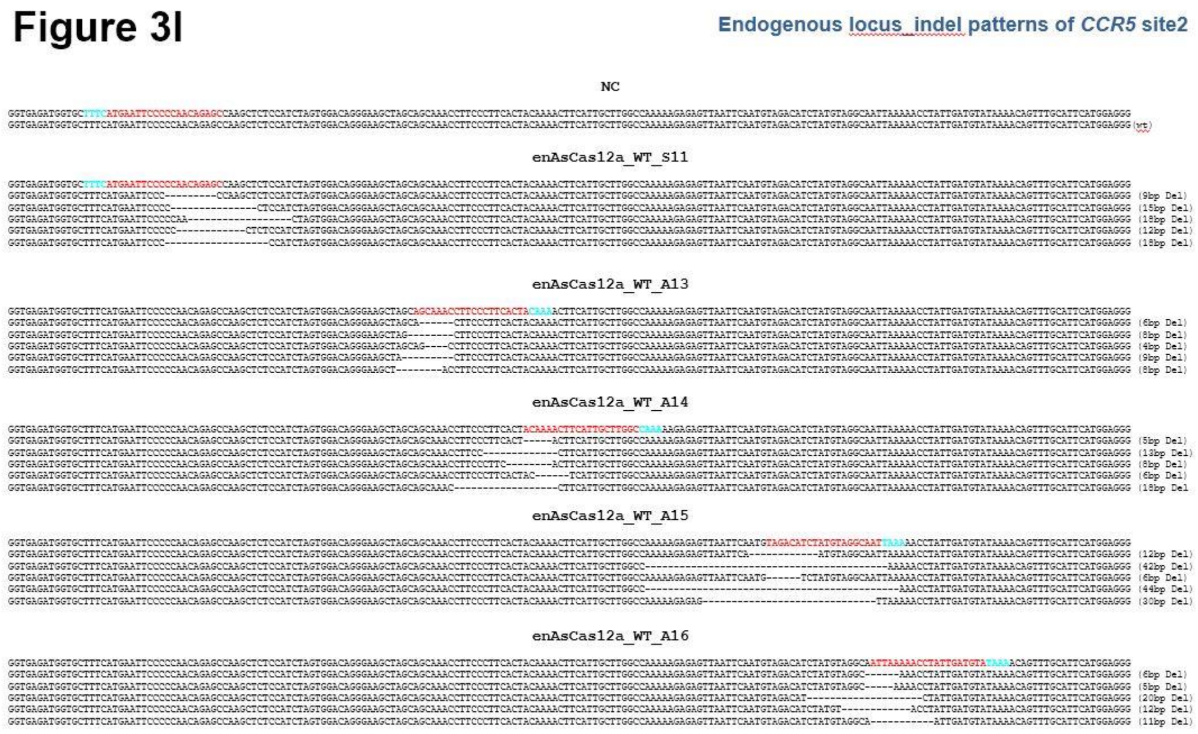

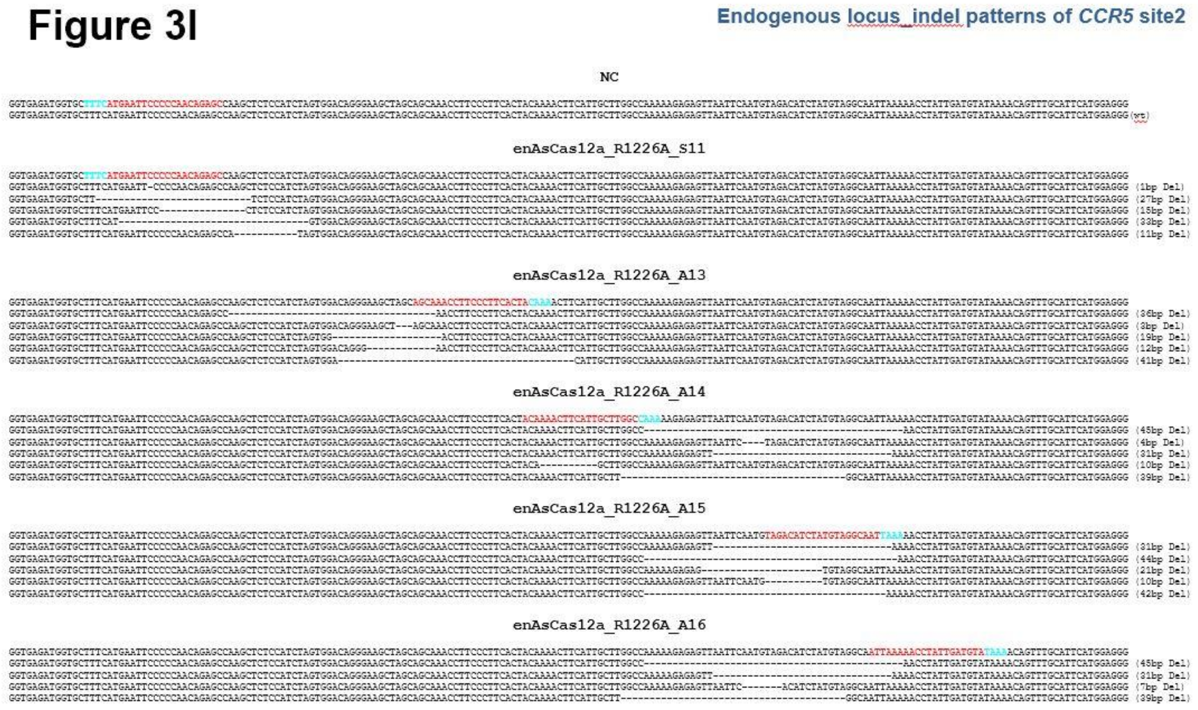

Supplementary Figure 4. Representative indel patterns induced by SpCas9 (D10A) or en-AsCas12a (R1226A) nickases on endogenous genes (*EMX1, CCR5)*. In main text Figure 3, each corresponding representative indel type is shown and matched number is indicated in the upper left corner of the sequencing result. PAM

and protospacer sequences recognized by SpCas9 (D10A) nickase are shown in magenta and pale green, respectively. The PAM and protospacer sequences recognized by en-AsCas12a (R1226A) nickase are shown in cyon and red, respectively. Deleted sequences are indicated by dotted lines.

Figure 4a

AAVS1

Indel read / Total read
4119/15689 (26.25%)

```
PAM        S20
CTCCCACCCCCTGCCAAGCTCTCCCTCCCAGGATCCTCTCTGGCTCCATCGTAAGCAAA
GAGGGTGGGGGACGGTTCGAGAGGGAGGGTCCTAGGAGAGACCGAGGTAGCATTCGTTT
                                             A20       PAM

CTCCCACCCCCTGCCAA-------------------------GCTCCATCGTAAGCAAA  (6.92%)
CTCCCACCCCCTG--------------------------------CCATCGTAAGCAAA  (5.07%)
CTCCCACCCCCTGCC-------------------------------------AAGCAAA  (4.81%)
CTCCCACCCCCTGCCAAGCT-----------------------CTCCATCGTAAGCAAA  (2.96%)
CTCCCACCCCCTGCCAAGCTCTCC-------------------CTCCATCGTAAGCAAA  (2.50%)
CTCCCACCCCCTGCCAAG---------------------CTGGCTCCATCGTAAGCAAA  (2.02%)
CTCCCACCCCCTGCCAAG-------------------CTCTGGCTCCATCGTAAGCAAA  (1.82%)
CTCCCACCCCCTGCCAAGCTCTCCCTC------------------CCATCGTAAGCAAA  (1.36%)
CTCCCACCCCCTGCCA-------------------------------ATCGTAAGCAAA  (1.24%)
CTCCCACCCCCTGCCAAGCTCTC----------------------CCATCGTAAGCAAA  (1.07%)
CTCCCACCCCCTGCCAAG---------------------------CCATCGTAAGCAAA  (0.92%)
CTCCCACCCCCT------------------------------------------GCAAA  (0.90%)
CTCCCACCCCCTGCCAAGCTCTCCC-----------------------TCGTAAGCAAA  (0.90%)
CTCCCACCCCCTGCCAAG----------------------------CATCGTAAGCAAA  (0.90%)
CTCCCACCCCCTGCCAAGC---------------------------------AAGCAAA  (0.87%)
CTCCCACCCCCTGCCAAGCTCTCCCTCCCAGGA-----------TCCATCGTAAGCAAA  (0.80%)
CTCCCACCCCCTG---------------------------------CATCGTAAGCAAA  (0.78%)
CTCCCACCCCCTGC---------------------------------------AGCAAA  (0.75%)
CTCCCACCCCCTGCCAAG-----------------CTCTCTGGCTCCATCGTAAGCAAA  (0.66%)
CTCCCACCCCCTGCCAAGC-----------------------------TCGTAAGCAAA  (0.66%)
CTCCCACCCCCTGCCAAGCTCTCCCTC------------CTGGCTCCATCGTAAGCAAA  (0.63%)
CTCCCACCCCCTGC-----------------------------------------CAAA  (0.63%)
CTCCCACCCC---------------------------------CTCCATCGTAAGCAAA  (0.61%)
CTCCCACCCCCTGCCAAGC-----------------------------------GCAAA  (0.61%)
CTCCCACCCCCTGCCAAGCTCTCCCTCCCA----------------CATCGTAAGCAAA  (0.56%)
CTCCCACCCCCTGCCAAGCTCTCC---------------CTGGCTCCATCGTAAGCAAA  (0.49%)
CTCCCACCCCCTGC-----------------------------CTCCATCGTAAGCAAA  (0.49%)
CTCCCACCCCCTGCCAAGCTCTCCCTCCCAG---------TGGCTCCATCGTAAGCAAA  (0.49%)
CTCCCACCCCCTGCCAAGCTCTCCCTCCCAG--------CTGGCTCCATCGTAAGCAAA  (0.49%)
CTCCCACCCCCTGCCAAGCTCTCCCTCCCA---------CTGGCTCCATCGTAAGCAAA  (0.39%)
CTCCCACCCCCTGCCAAGCTCTCCCTCCCA--------------TCCATCGTAAGCAAA  (0.39%)
CTCCCACCCCCTGCCAA---------------------------CCCATCGTAAGCAAA  (0.36%)
CTCCCACCCCCTGCCAAGCTC--------------------------------------  (0.36%)
CTCCCACCCCCTG----------------------------------------------  (0.36%)
CTCCCACCCCCTGCCAAGCTCTCCCTCC---------------CTCCATCGTAAGCAAA  (0.36%)
CTCCCACCCCCTGCCAAGCTCTCCCTCCCA-----------GGCTCCATCGTAAGCAAA  (0.36%)
CTCCCACCCCCTGCCAAG------------GGATCCTCTCTGGCTCCATCGTAAGCAAA  (0.34%)
CTCCCACCCCCTGCCAAGCTCTCCCTCC-----------CTGGCTCCATCGTAAGCAAA  (0.32%)
CTCCCACCCCCTGC-------------------------CTGGCTCCATCGTAAGCAAA  (0.32%)
CTCCCACCCCCTGCCAAGCTCTCCCTCCCA------------GCTCCATCGTAAGCAAA  (0.32%)
CTCCCACCCCCTGCCAAGCT--------------------TG------------GCAAA  (0.29%)
CTCCCACCCCCTGCCAA------------------------GGCTCCATCGTAAGCAAA  (0.27%)
CTCCCACCCCCTGCC--------------------------------------------  (0.27%)
```

Figure 4b

Indel read / Total read
7148/16877 (42.35%)

CCR5

```
PAM            S21

TTTATGCACAGGGTGGAACAAGATGGATTATCAAGTGTCAAGTCCAATCTATGACATCAATTATTATACATCGGAGCCCTGCCAAA
AAATACGTGTCCCACCTTGTTCTACCTAATAGTTCACAGTTCAGGTTAGATACTGTAGTTAATAATATGTAGCCTCGGACGGTTT

                                                                           A21     PAM

TTTATGCACAGGGTGGA--------------------------------------------------ACATCGGAGCCCTGCCAAA (5.11%)
TTTATGCACAGGGTGGAAC----------------------------------------------ATACATCGGAGCCCTGCCAAA (2.94%)
TTTATGCACAGGGTGGAACA---------------------------------------------ATACATCGGAGCCCTGCCAAA (2.50%)
TTTATGCACAGGGTGGAACAAGATGGATTATCAAGTGTCAAGTCCAATCTATG--------------ACATCGGAGCCCTGCCAAA (2.48%)
TTTATGCACAGGGTGGAACAAG-------------------------------------------ATACATCGGAGCCCTGCCAAA (2.17%)
TTTATGCACAGGGTGGAACAAGATGGATTATC---------------------------------ATACATCGGAGCCCTGCCAAA (2.11%)
TTTATGCACAGGGT----------------------------------------------------------GGAGCCCTGCCAAA (2.00%)
TTTATGCACAGGGTGGAACAAGATGG-------------------------------------------ATCGGAGCCCTGCCAAA (1.79%)
TTTATGCACAGGGTG-----------------------------------------------------CATCGGAGCCCTGCCAAA (1.62%)
TTTATGCACAGGGTGGAA---------------------------------------CAATTATTATACATCGGAGCCCTGCCAAA (1.32%)
TTTATGCACAGGGTGGAACAAGATGGATTATCAAGTGTCAAGTCCA-----------------------ATCGGAGCCCTGCCAAA (1.16%)
TTTATGCACAGGGTGGAACAAGA--------------------------------------------------------TGCCAAA (1.09%)
TTTATGCACAGGGTGGAACAAGATGG------------------------------------ATTATACATCGGAGCCCTGCCAAA (1.08%)
TTTATGCACAGGGTGGAACAAGATGGA------------------------------------------ATCGGAGCCCTGCCAAA (0.94%)
TTTATGCACAGGGTGGAACAAG--------------------------------------------TACATCGGAGCCCTGCCAAA (0.92%)
TTTATGCACAGGG-----------------------------------------------------TACATCGGAGCCCTGCCAAA (0.90%)
TTTATGCACAGGGTGGAACAAGATG------------------------------------------ACATCGGAGCCCTGCCAAA (0.88%)
TTTATGCACAGGGTGGAAC------------------------------------------------------GAGCCCTGCCAAA (0.88%)
TTTATGCACAGGGTGGAACAAGATGGATTATCAAGTGTCAAGTCCAATC---------------TATACATCGGAGCCCTGCCAAA (0.88%)
TTTATGCACAGGGTGG---------------------------------------------------ACATCGGAGCCCTGCCAAA (0.85%)
TTTATGCACAGGG-------------------------------------------------------CATCGGAGCCCTGCCAAA (0.85%)
TTTATGCACAGGGTGG-------------------------------------------------ATACATCGGAGCCCTGCCAAA (0.80%)
TTTATGC------------------------------------------------------------ACATCGGAGCCCTGCCAAA (0.78%)
TTTATGCACAGGGTGGAACAAGATGGATTATCAAGTGTCAAGTCCAATCTATGAC----------ATACATCGGAGCCCTGCCAAA (0.78%)
TTTATGCACAGGGT-----------------------------------------------------------GAGCCCTGCCAAA (0.78%)
TTTATGCACAGGGTGGAACAAGA-----------------------------------------TATACATCGGAGCCCTGCCAAA (0.77%)
TTTATGCACAGGGTGGAACAAGATGGATTATCAAGTGTCAAGTCCAATCTATGACATCA---ATTATACATCGGAGCCCTGCCAAA (0.76%)
TTTATGCACAGGGTGGAAC-------------------------------------------ATTATACATCGGAGCCCTGCCAAA (0.74%)
TTTATGCACAGGGTGGAACAAGATGGA--------------------------------------------CGGAGCCCTGCCAAA (0.70%)
TTTATGCACAGGG---------------------------------------------------TATACATCGGAGCCCTGCCAAA (0.69%)
TTTATGCACAGGGTGG-----------------------------------------------------ATCGGAGCCCTGCCAAA (0.69%)
TTTATGCACAGGGTGGAAC------------------------------------------------ACATCGGAGCCCTGCCAAA (0.67%)
TTTATGCACAGG--------------------------------------------------------------------GCCAAA (0.64%)
TTTATGCACAGGGTGGAACAAG-----------------------------------------------ATCGGAGCCCTGCCAAA (0.64%)
TTTATGCACAGGGTGGAACAAGATGGATTATCAAGTGT-------------------------------------GCCCTGCCAAA (0.63%)
TTTATGCACAGGGTGGAACAAGATGGATTATCAAGTGTCAAGTCCAATC-----------------TACATCGGAGCCCTGCCAAA (0.63%)
TTTATGCACAGGGTGG--------------------------------------------------TACATCGGAGCCCTGCCAAA (0.60%)
TTTATGCACAGGGTG---------------------------------------------------TACATCGGAGCCCTGCCAAA (0.59%)
TTTATGCACAGGGTGGAACAAGATGGA-------------------------------------------------CCCTGCCAAA (0.57%)
TTTATGCACAGGGTGGAACAAGATGGATTATCAAGTGTCAAGTCCA-------------------ATACATCGGAGCCCTGCCAAA (0.56%)
TTTATGCACAGGGTGGAACAAGATGGATTATCAAGTGTCAAG----------------------------TCGGAGCCCTGCCAAA (0.53%)
TTTATGCACAGGGTGGAACA-----------------------------------------------ACATCGGAGCCCTGCCAAA (0.52%)
TTTATGCACAGG----------------------GTGTCAAGTCCAATCTATGACATCAATTATTATACATCGGAGCCCTGCCAAA (0.52%)
TTTATGCACAGGGGG-------------------------------------------------------TCGGAGCCCTGCCAAA (0.52%)
```

Figure 4c

```
PAM          S22                 Indel read / Total read                    DNMT1
                                  2601/10596 (24.54%)
GTTCTAGACCCAGAGGCTCAAGTGAGCAGCTGAGGCAGGTGCCTGCTGAGCCAAA
CAAGATCTGGGTCTCCGAGTTCACTCGTCGACTCCGTCCACGGACGACTCGGTTT
                                        A22          PAM

G------------------------------------------------------ (9.07%)
GTTCTAGACCCAGAGGCTCAAG--------TGAGGCAGGTGCCTGCTGAGCCAAA (6.88%)
GTTCTAGACCCAGAGGCTCAA-----------------GTGCCTGCTGAGCCAAA (6.46%)
GTTCTAGACCCAGAGGCTCAAG------------------------TGAGCCAAA (4.27%)
GT----------------------------------------------------- (3.34%)
GTTCTAGACCCA-------------------GAGGCAGGTGCCTGCTGAGCCAAA (2.58%)
GTTCTAGACCCAG------------------------------------------ (2.42%)
GTTCTAGACCCAGAGGCT-----------------CAGGTGCCTGCTGAGCCAAA (2.38%)
GTTCTAGACCCAGA----------------------------------------- (2.31%)
GTTCTAGACCCAGAGGCTCAAGTGAGCA-CTGAGGCAGGTGCCTGCTGAGCCAAA (1.96%)
GTTCTAGACCCA------------------------------------------- (1.88%)
GTTCTAG------------------------------------------------ (1.88%)
GTTCTAGACCCAGAGGCTCAAGTGA---------GCAGGTGCCTGCTGAGCCAAA (1.85%)
GTTCTAGACCCAGAGGCTCAAGTG---AGCTGAGGCAGGTGCCTGCTGAGCCAAA (1.85%)
-------------------------------------GGTGCCTGCTGAGCCAAA (1.77%)
G---------------------------------------------TGAGCCAAA (1.65%)
GTTCTAGACCCAGAGGCTCAAGT-----GCTGAGGCAGGTGCCTGCTGAGCCAAA (1.61%)
GTTCTAGACCCAGAGGCTCAAGTG------------AGGTGCCTGCTGAGCCAAA (1.58%)
GTTCTAGACCCAGAGGCTCAAGTGAGCA---GAGGCAGGTGCCTGCTGAGCCAAA (1.38%)
GTTCTAGACCC-------------------------------------------A (1.31%)
GTTC-----------------------------------TGCCTGCTGAGCCAAA (1.31%)
GTTCTAGACCCAGAGGC-------------------------------------- (1.27%)
GTTCTAGACCCAGAGGCT------------------------------------- (1.11%)
GTTCTAGA------------------------------------------CCAAA (1.04%)
GTTCTAGACCC-------------------------------------AGCCAAA (1.04%)
GTTCT-------------------------------------------AGCCAAA (0.92%)
GTTCTAGACCCAGAGGCTCAAGTGAGCA------GCAGGTGCCTGCTGAGCCAAA (0.88%)
GTTCTAGACCCAG-----------------------AGGTGCCTGCTGAGCCAAA (0.88%)
GTT------------------------------------TGCCTGCTGAGCCAAA (0.85%)
GTTCTAGACCCAGAGGCTCAAGTGAGC-----AGGCAGGTGCCTGCTGAGCCAAA (0.81%)
--------------------------CAGCTGAGGCAGGTGCCTGCTGAGCCAAA (0.81%)
GTTCTAGACCCAGAG-----------------------------GCTGAGCCAAA (0.81%)
GTTCAA-------------------------GAGGCAGGTGCCTGCTGAGCCAAA (0.77%)
GTTCTAGAC-----------------------------------------CCAAA (0.73%)
------CACC-------------------------GAGGTGCCTGCTGAGCCAAA (0.73%)
GTTCTAGACCCAGAGGCTCAAGTGAGCA-------CTGGTGCCTGCTGAGCCAAA (0.73%)
GTTCTAGACCCAGAGGCTCAAGTGAGC-----AGTCAGGTGCCTGCTGAGCCAAA (0.65%)
GTTCTAGACCCAGAGGCTCAAGTGAGCA----------------GCTGAGCCAAA (0.65%)
GTTCTAGACC------------------------GCAGGTGCCTGCTGAGCCAAA (0.62%)
GTTCTAGACCCAGAGGCTCAAGTGAGCA----------GTGCCTGCTGAGCCAAA (0.62%)
GTTCTAGACCCAGAGGCTCAAGT-------------------------------- (0.62%)
GTTCTAGACCCAGAGGCTCA----------------AGGTGCCTGCTGAGCCAAA (0.58%)
GTT-----------------------------------------------CCAAA (0.58%)
```

Supplementary Figure 5. Representative indel patterns induced by dual-en-AsCas12a (R1226A) nickases on endogenous genes (*AAVS1, CCR5, DNMT1*). In main text Figure 4, three representative indel type is shown and matched number is indicated in the upper left corner of the sequencing result. Deleted sequences are indicated by dotted lines. The PAM and protospacer sequences recognized by en-AsCas12a (R1226A) nickase are shown in red and black upper line, respectively.

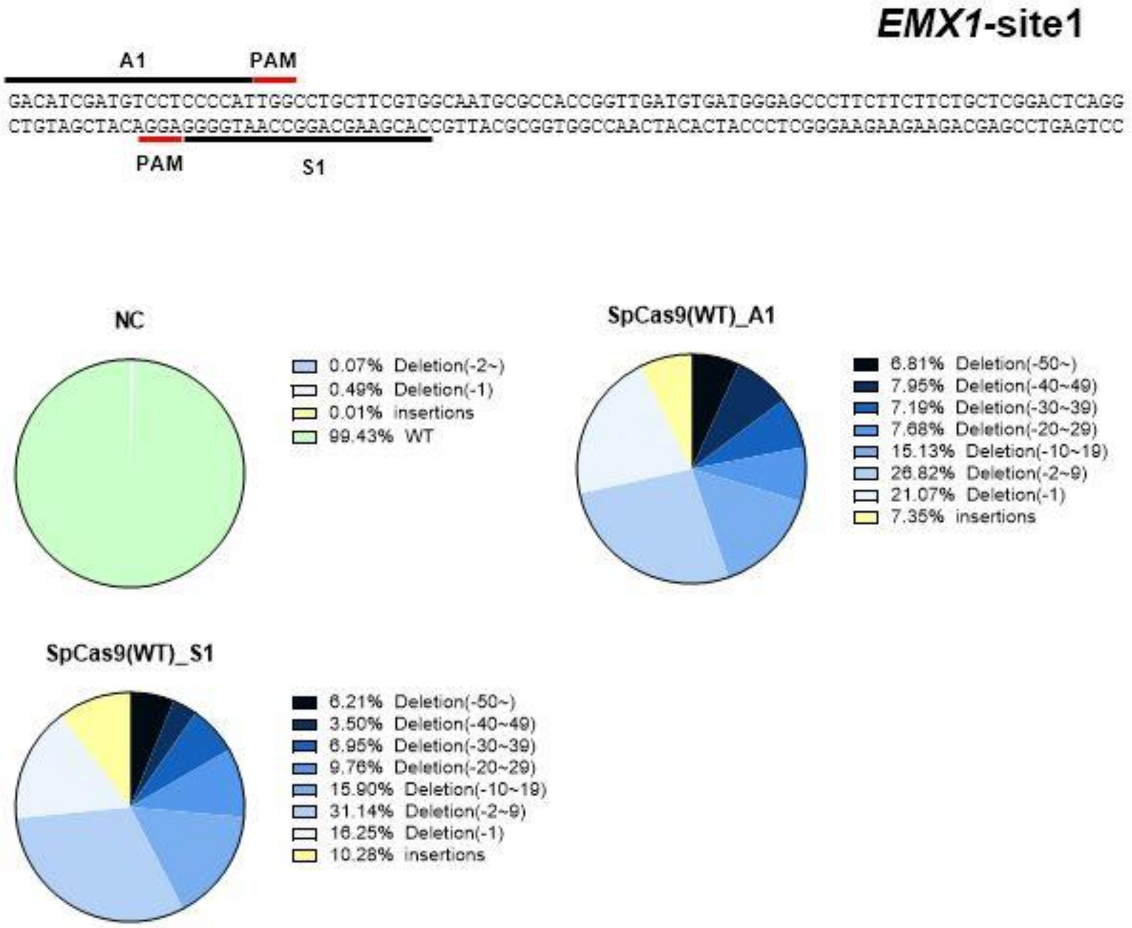

Figure 2a-b, 3d

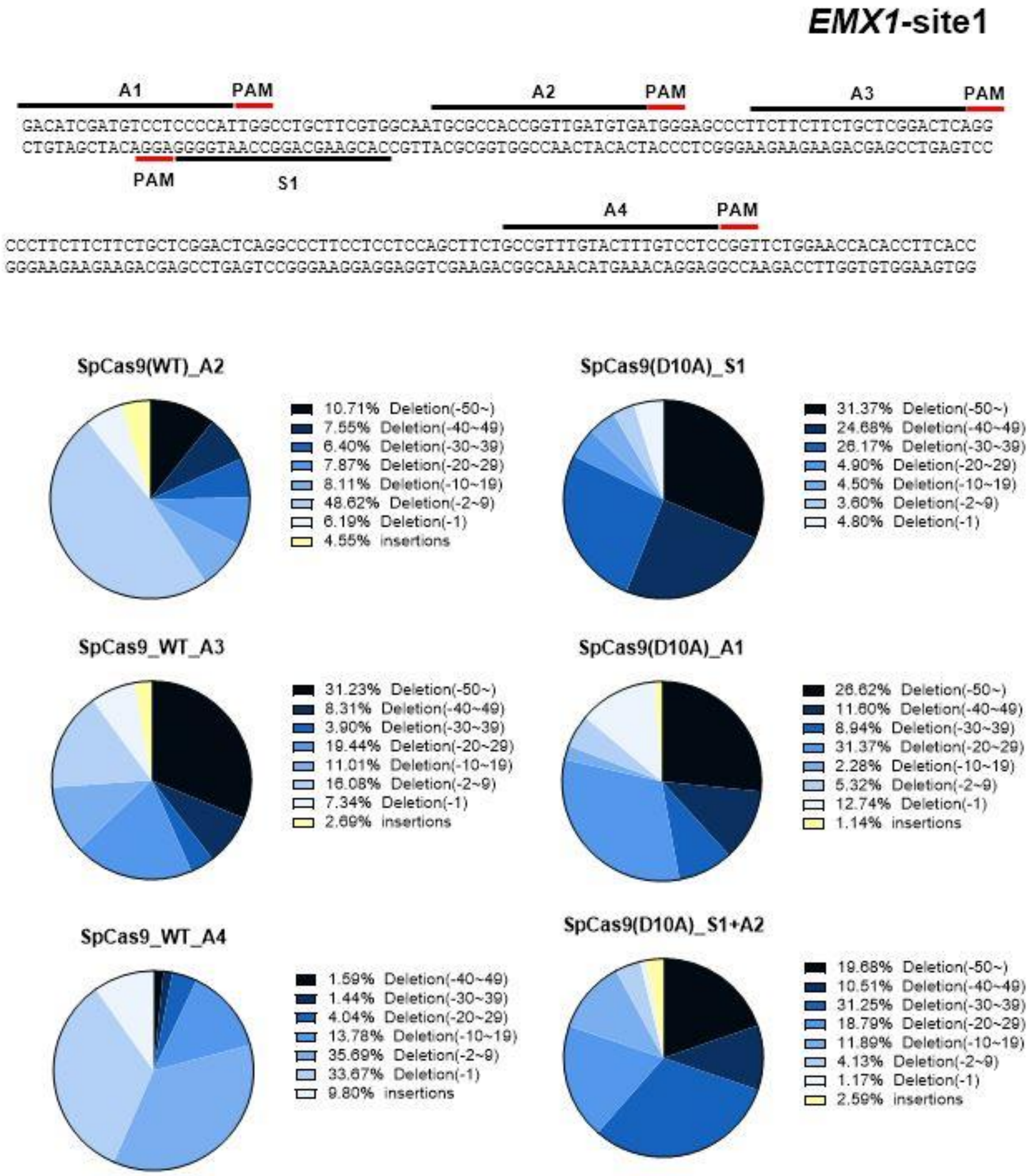

Figure 2a-b, 3d

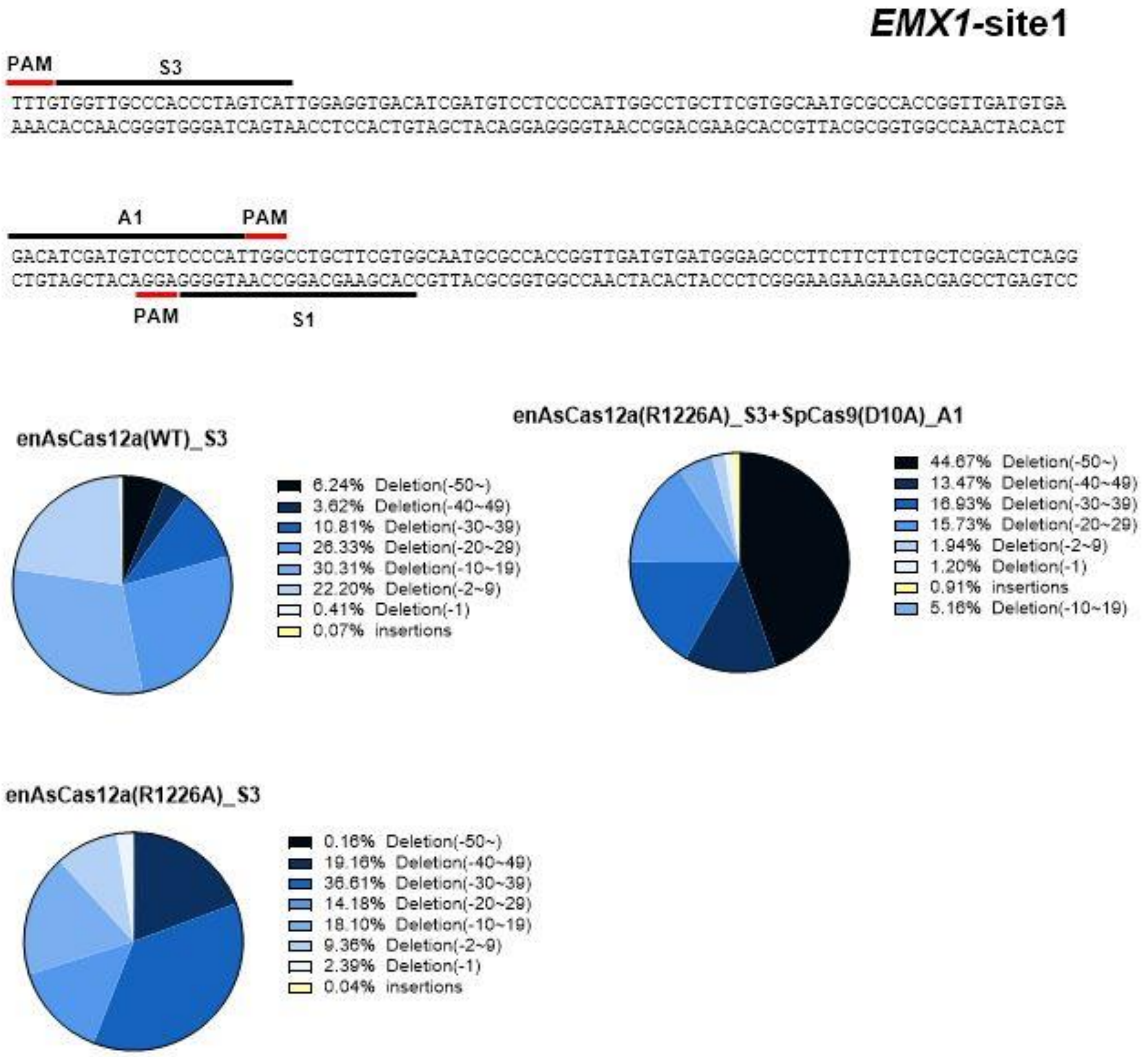

Figure 2c-d, 3e

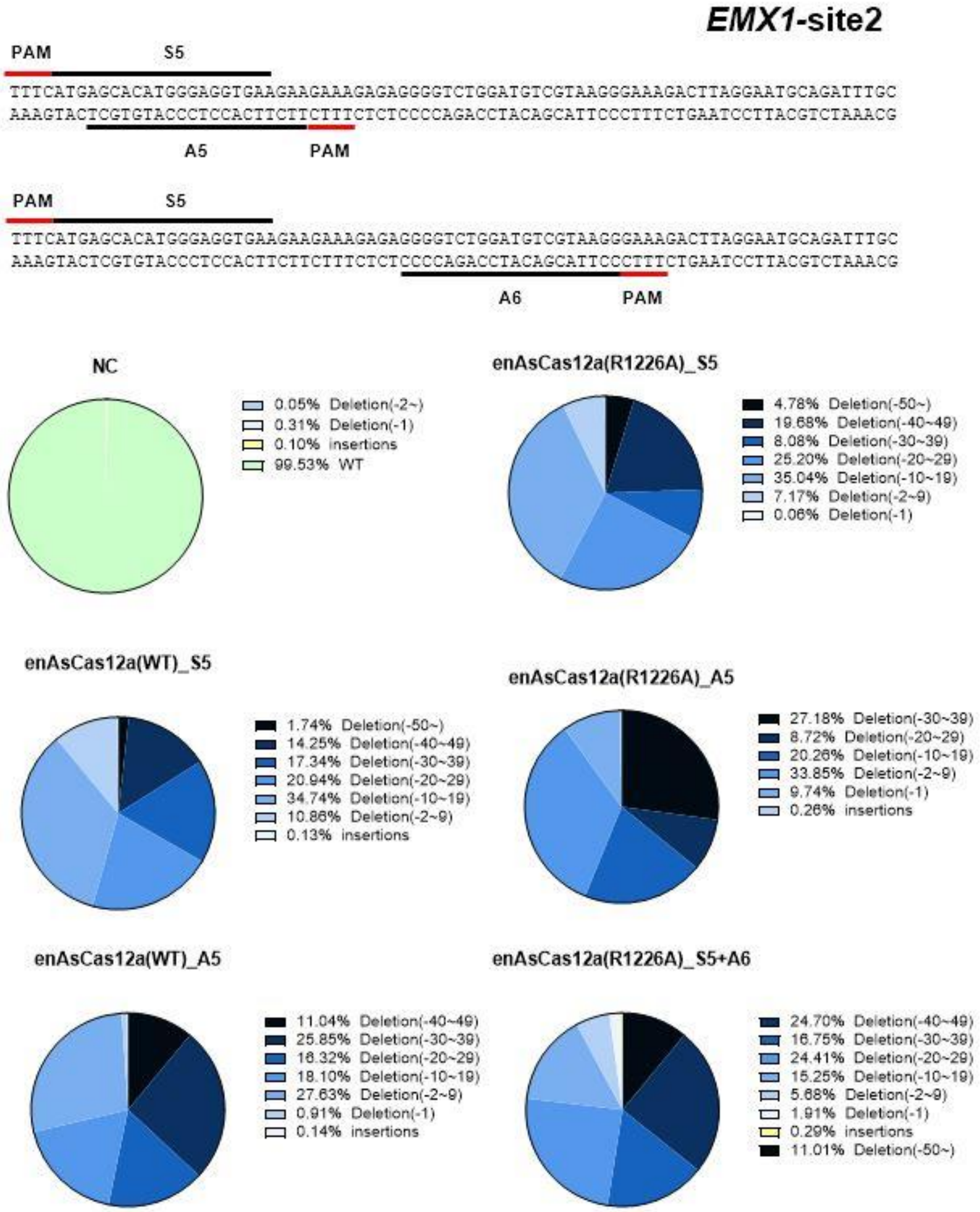

Figure 2e-f, 3f

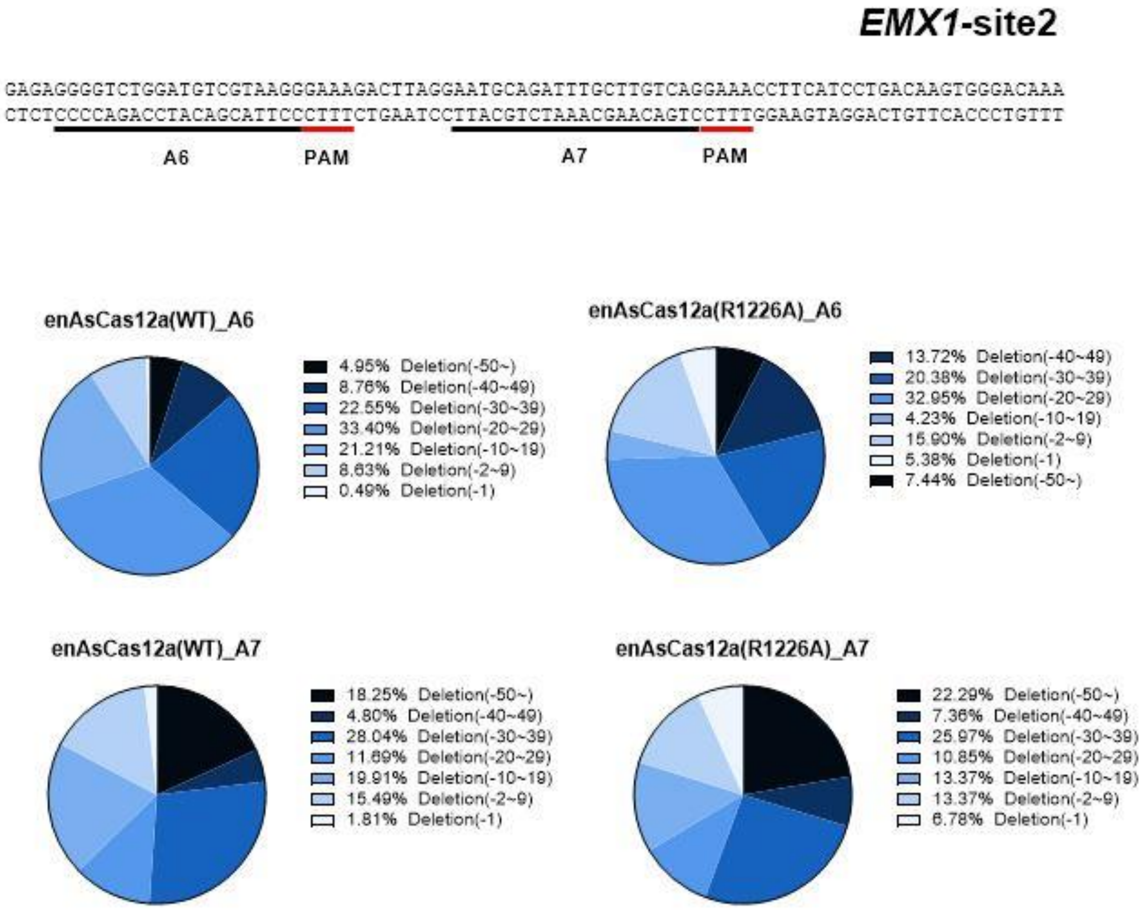

Figure 2e-f, 3f

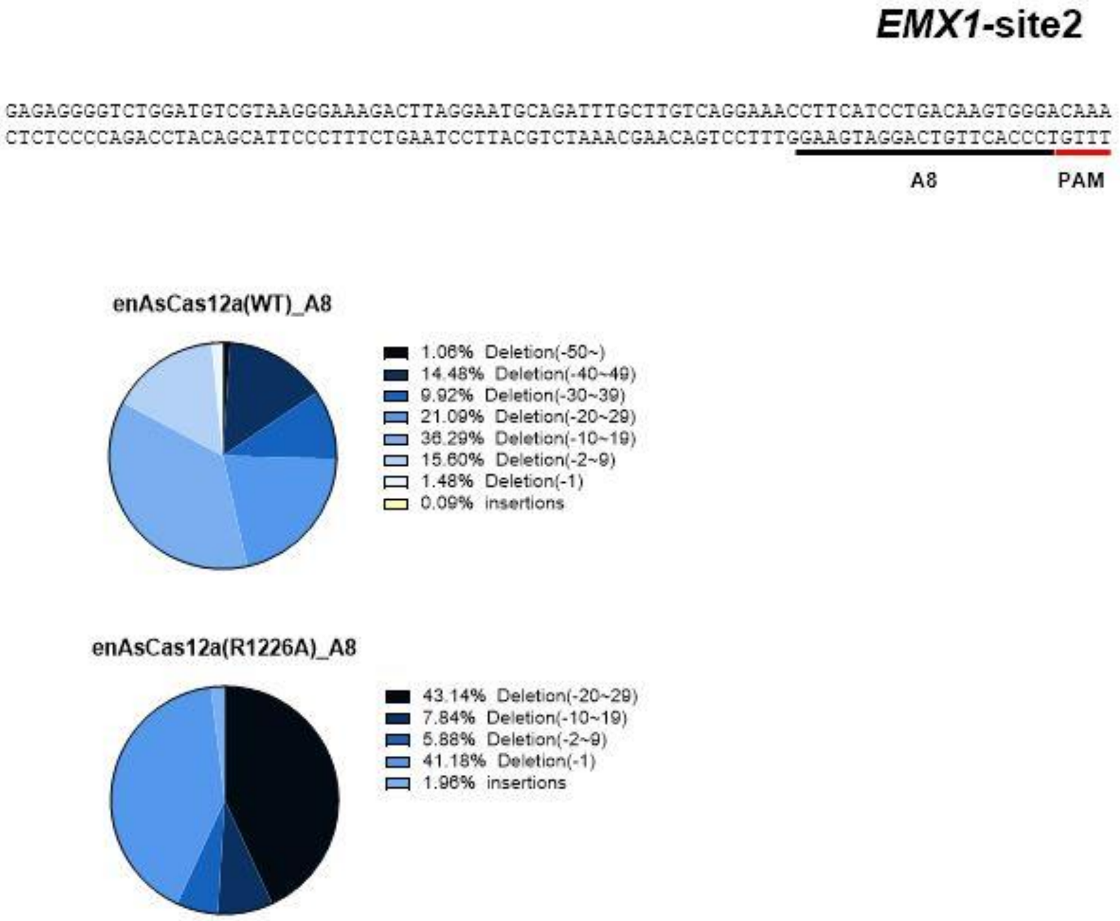

Figure 2e-f, 3f

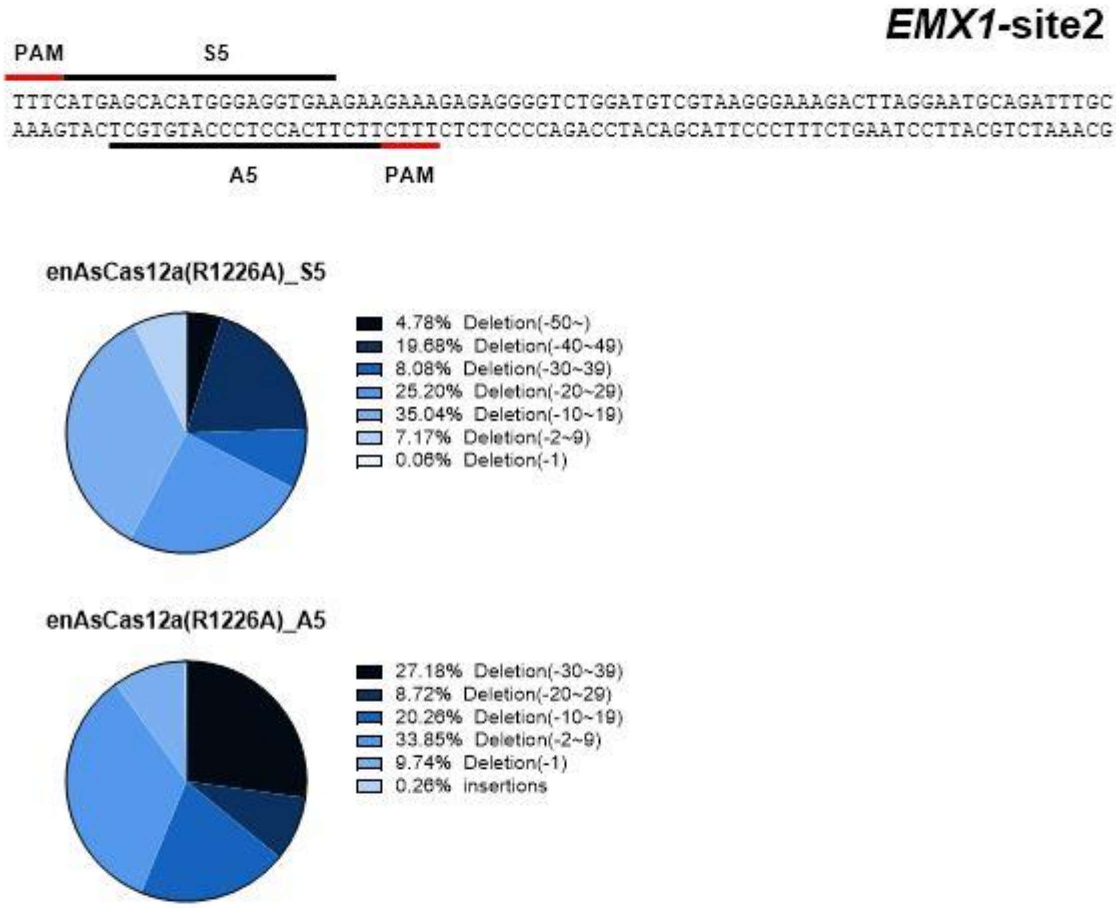

Figure 2e-f, 3f

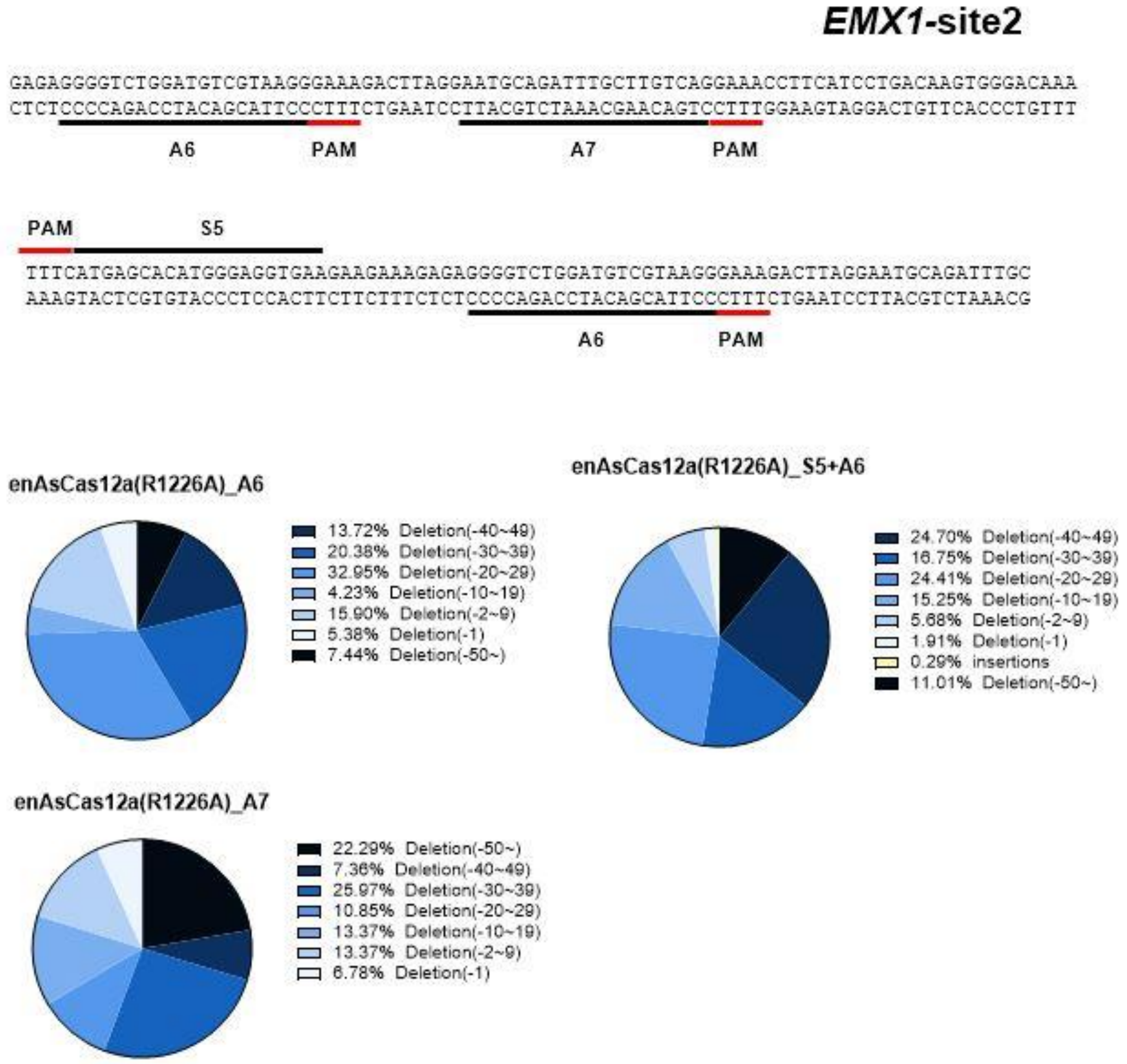

Figure 2e-f, 3f

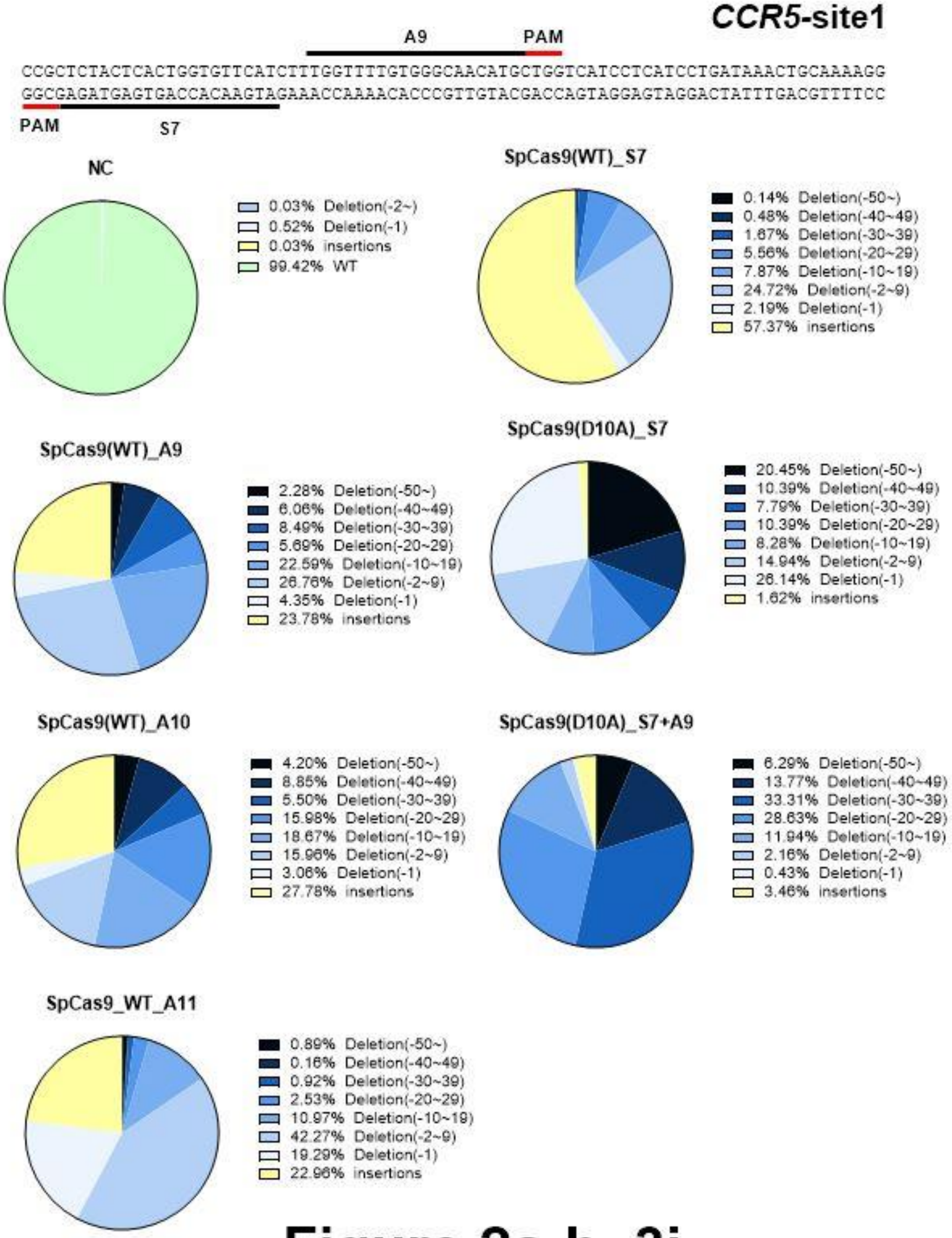

Figure 2a-b, 3j

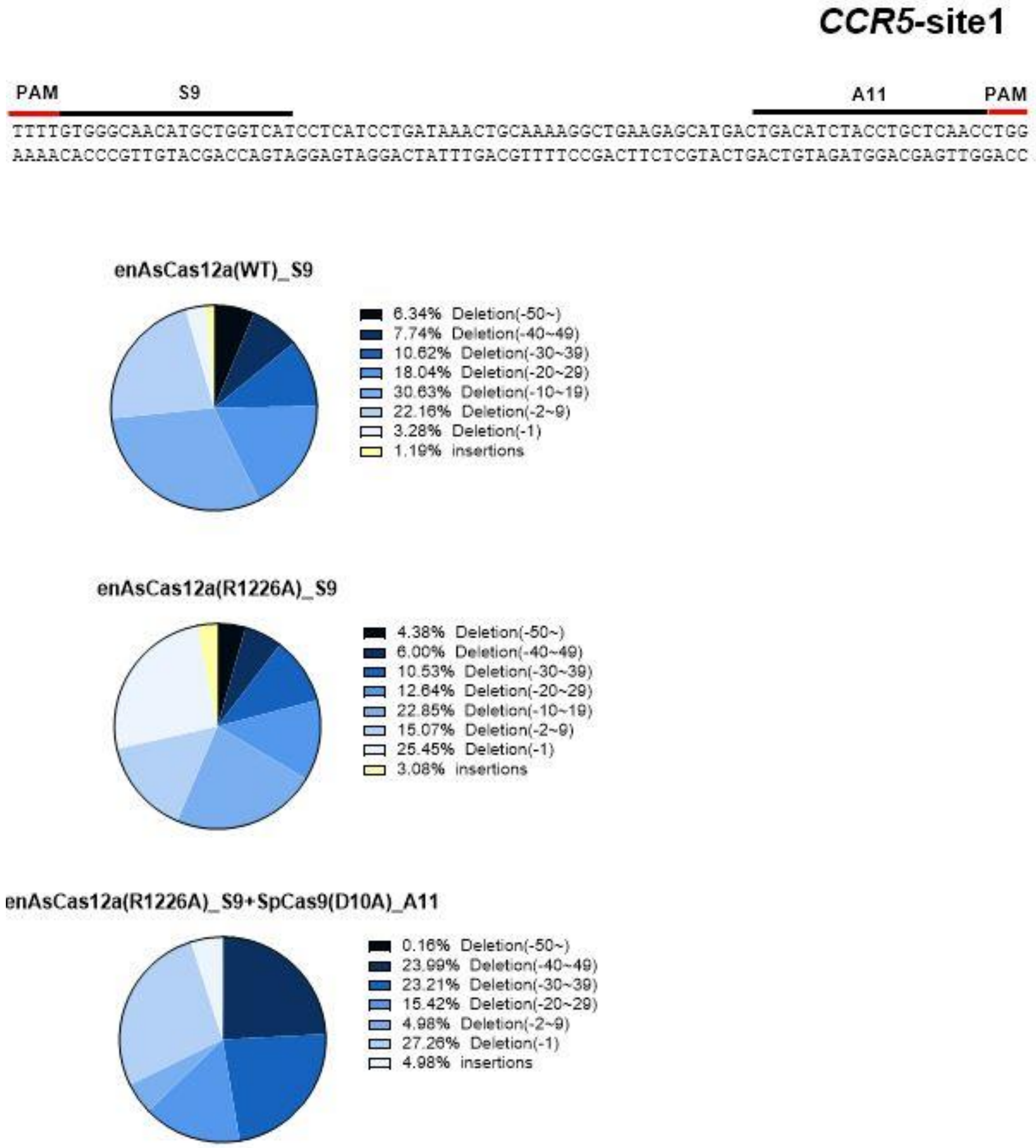

Figure 2c-d, 3k

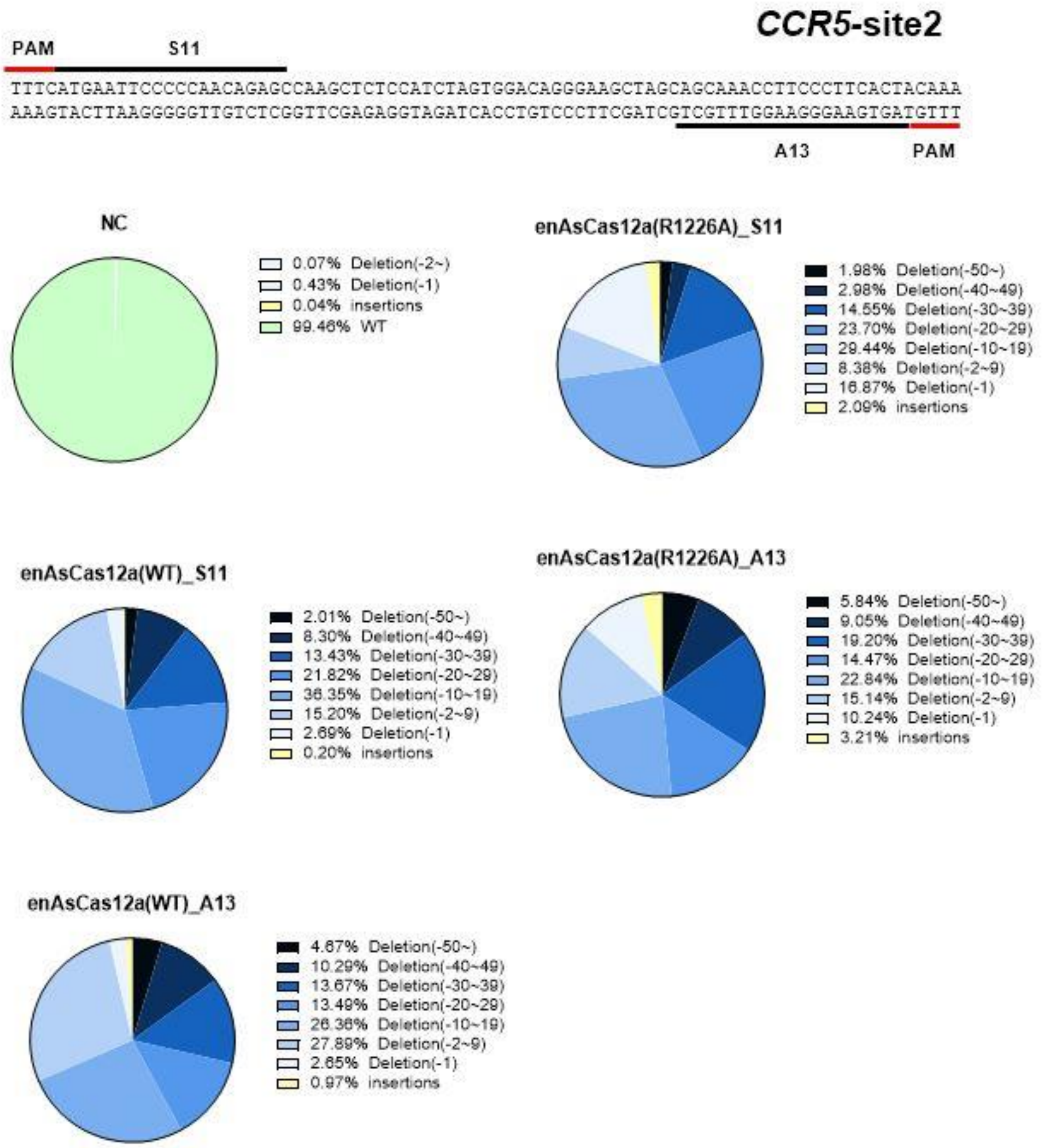

Figure 2e-f, 3l

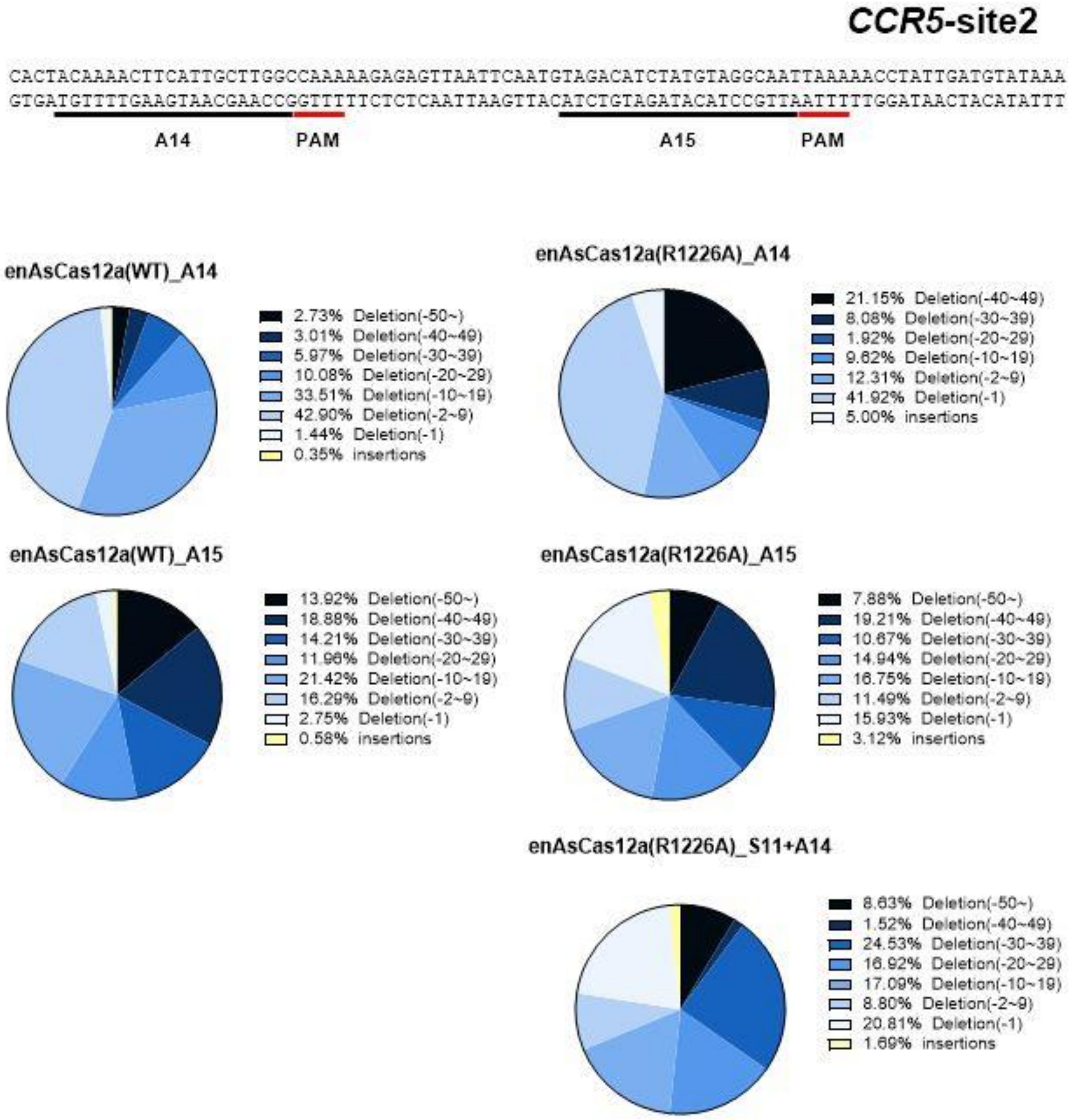

Figure 2e-f, 3l

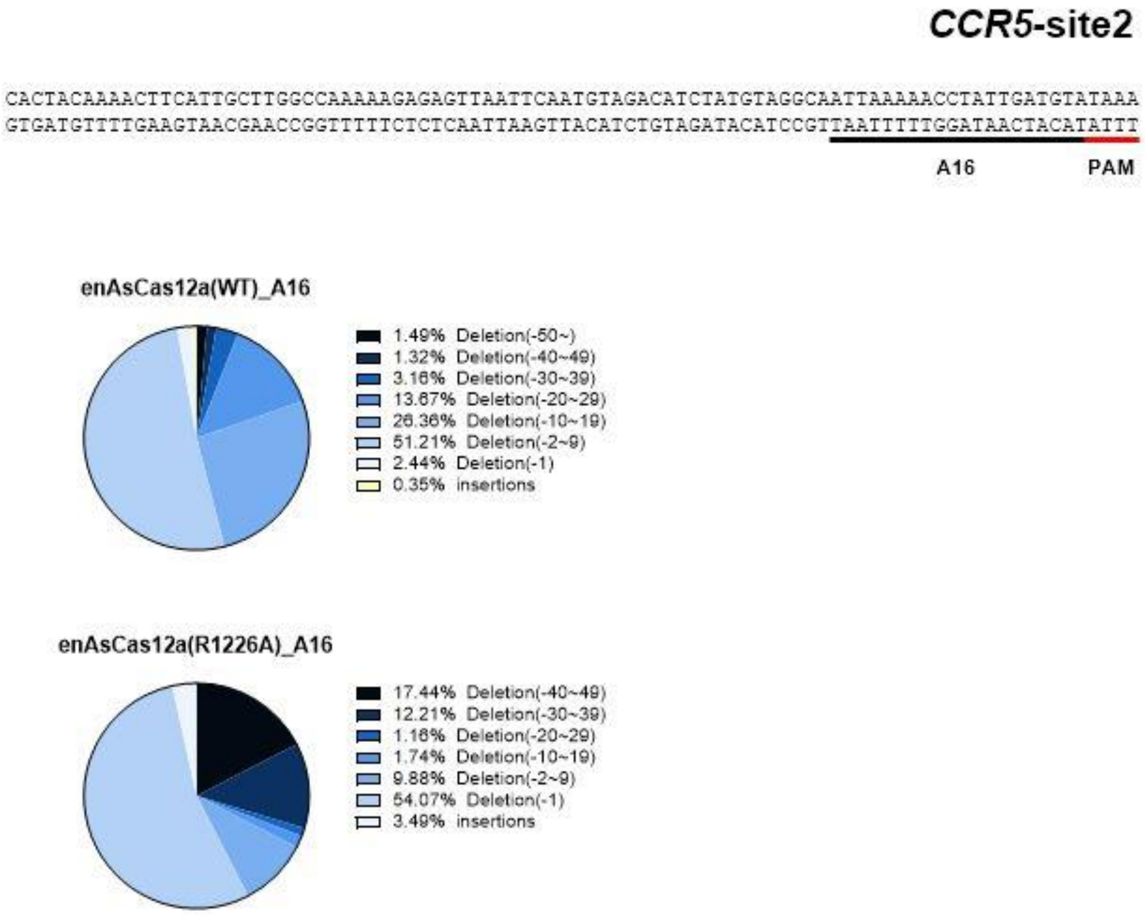

Figure 2e-f, 3l

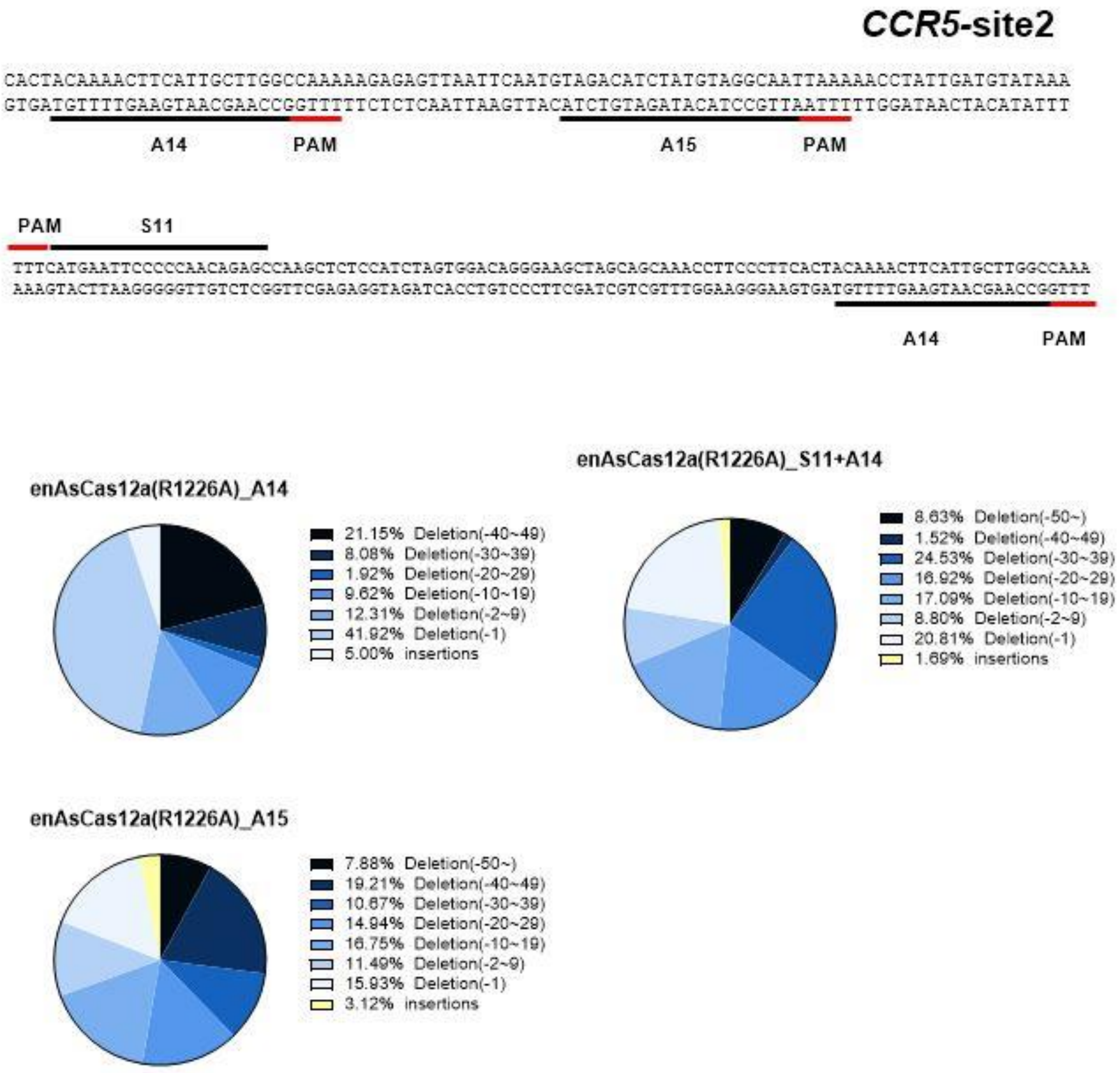

Figure 2e-f, 3l

Supplementary Figure 6. Result of the analysis of indel patterns generated by SpCas9 (D10A) or en-AsCas12a (R1226A) nickases. Top: Schematic of SpCas9 (D10A), SpCas9 (WT), en-AsCas12a (R1226A) and en-AsCas12a (WT) target sequences in each gene (*EMX1* and *CCR5* in Figure 2, 3). The PAM and protospacer sequences recognized by SpCas9 (D10A) or en-AsCas12a (R1226A) nickase are shown in red and black upper line, respectively. Bottom: Results showing the indel pattern in each target (*EMX1* and *CCR5* in Figure 2, 3) generated by SpCas9 (D10A), SpCas9 (WT), en-AsCas12a (R1226A) and en-AsCas12a (WT)

in a pie chart. As the color became darker, the mutation in the deletion form that occurred was largely displayed. NC: negative control, SpCas9 (D10A): SpCas9 nickase, SpCas9 (WT): wild-type SpCas9, en-AsCas12a (R1226A): en-AsCas12a nickase, en-AsCas12a (WT): wild-type en-AsCas12a. In Figures 2 and 3 of the main text, each corresponding indel pattern was analyzed and the corresponding figure number was displayed at the bottom of the pie chart.

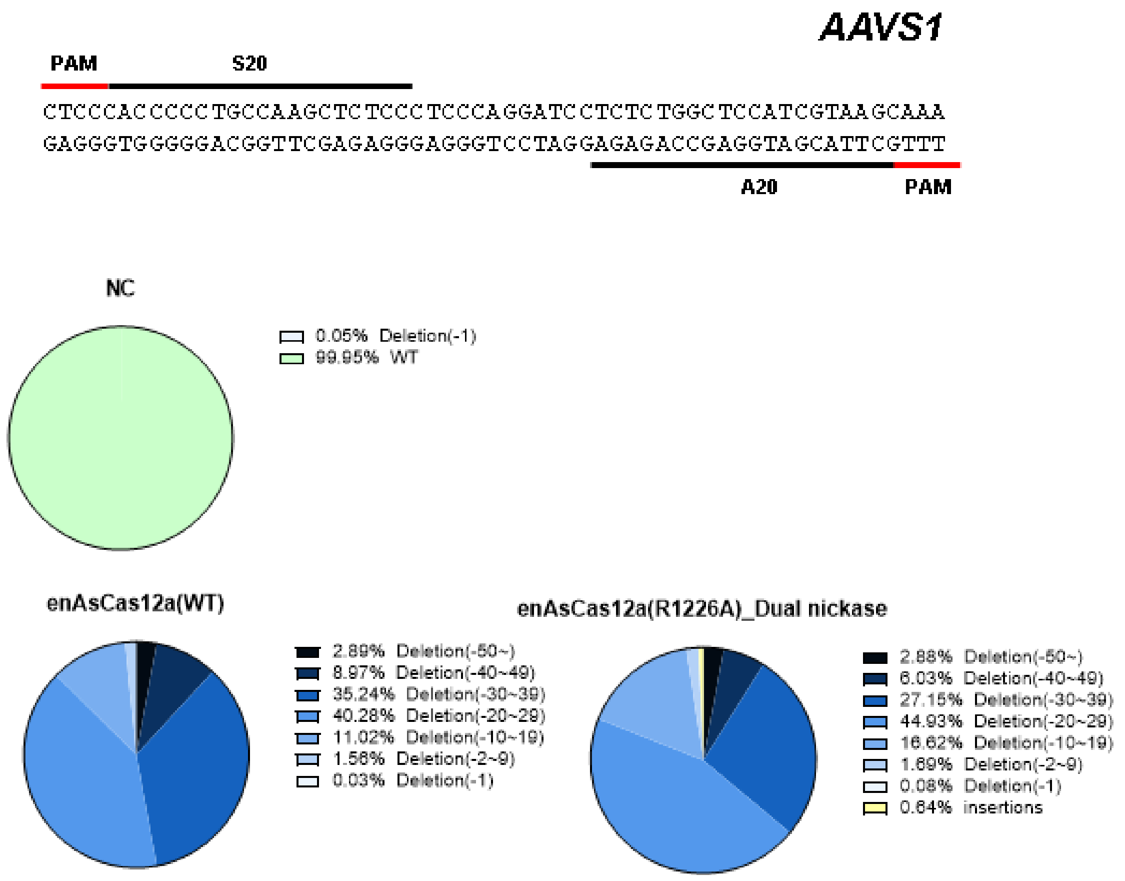

AAVS1
PAM
S20
CTCCCACCCCCTGCCAAGCTCTCCCTCCCAGGATCCTCTCTGGCTCCATCGTAAGCAAA
GAGGGTGGGGGACGGTTCGAGAGGGAGGGTCCTAGGAGAGACCGAGGTAGCATTCGTTT
A20
PAM
NC
0.05% Deletion(-1)
99.95% WT
enAsCas12a(WT)
2.89% Deletion(-50~)
8.97% Deletion(-40~49)
35.24% Deletion(-30~39)
40.28% Deletion(-20~29)
11.02% Deletion(-10~19)
1.56% Deletion(-2~9)
0.03% Deletion(-1)
enAsCas12a(R1226A)_Dual nickase
2.88% Deletion(-50~)
6.03% Deletion(-40~49)
27.15% Deletion(-30~39)
44.93% Deletion(-20~29)
16.62% Deletion(-10~19)
1.69% Deletion(-2~9)
0.08% Deletion(-1)
0.64% insertions

Figure 4a

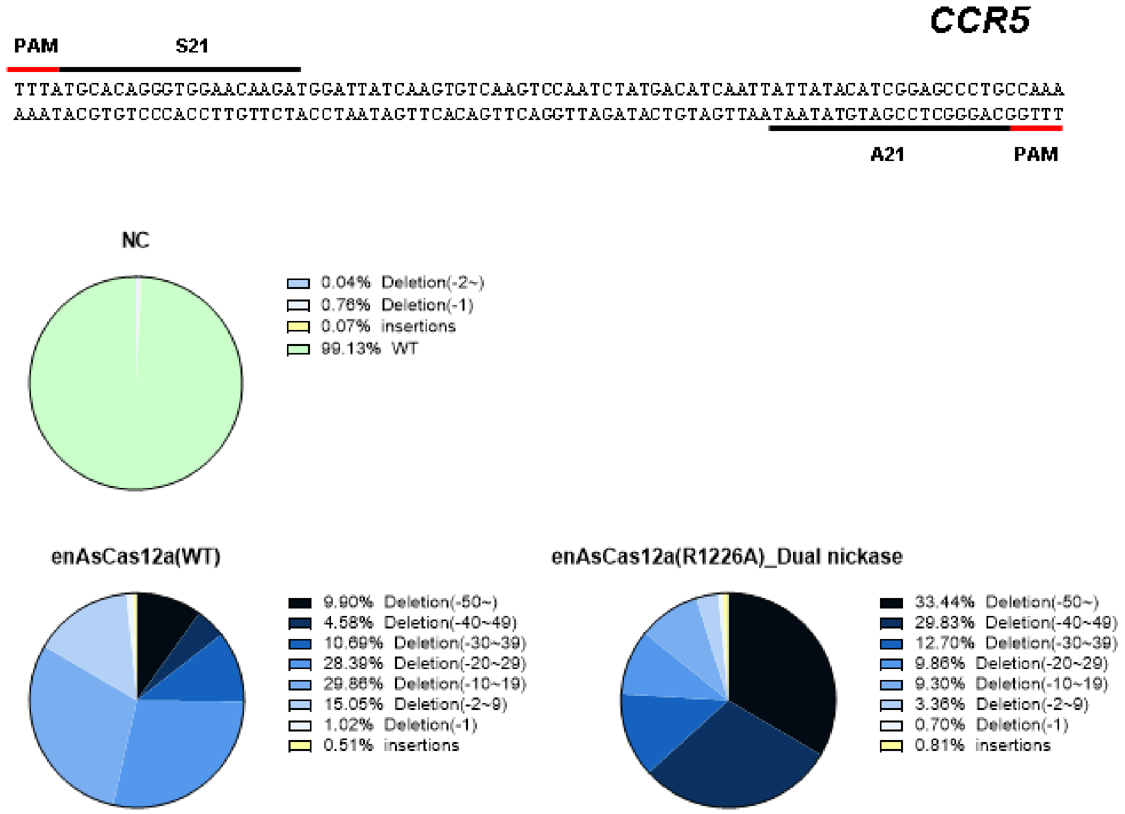
CCR5
PAM
S21
TTTATGCACAGGGTGGAACAAGATGGATTATCAAGTGTCAAGTCCAATCTATGACATCAATTATTATACATCGGAGCCCTGCCAAA
AAATACGTGTCCCACCTTGTTCTACCTAATAGTTCACAGTTCAGGTTAGATACTGTAGTTAATAATATGTAGCCTCGGGACGGTTT
A21
PAM
NC
0.04% Deletion(-2~)
0.76% Deletion(-1)
0.07% insertions
99.13% WT
enAsCas12a(WT)
9.90% Deletion(-50~)
4.58% Deletion(-40~49)
10.69% Deletion(-30~39)
28.39% Deletion(-20~29)
29.86% Deletion(-10~19)
15.05% Deletion(-2~9)
1.02% Deletion(-1)
0.51% insertions
enAsCas12a(R1226A)_Dual nickase
33.44% Deletion(-50~)
29.83% Deletion(-40~49)
12.70% Deletion(-30~39)
9.86% Deletion(-20~29)
9.30% Deletion(-10~19)
3.36% Deletion(-2~9)
0.70% Deletion(-1)
0.81% insertions

Figure 4b

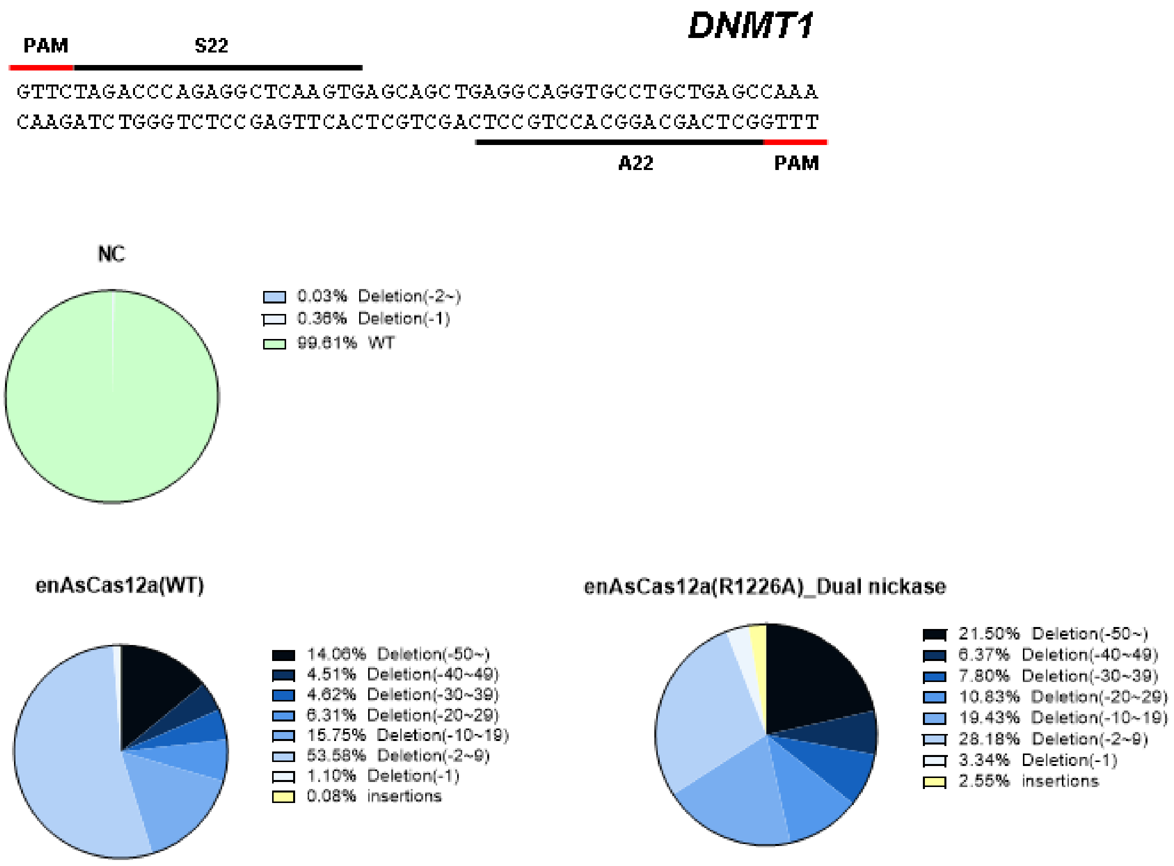

Figure 4c

Supplementary Figure 7. Result of the analysis of indel patterns generated by en-AsCas12a (R1226A) nickase. Top: Schematic of en-AsCas12a (R1226A) or en-AsCas12a (WT) target sequences in each gene (*AAVS1, CCR5* and *DNMT1* in Figure 4). The PAM and protospacer sequences recognized by en-AsCas12a (R1226A) nickase are shown in red and black, respectively. Bottom: Results showing the indel pattern in each target (*AAVS1, CCR5* and *DNMT1* in Figure 4) generated by en-AsCas12a (R1226A) nickase in a pie chart. As the color became darker, the mutation in the deletion form that occurred was largely displayed. NC: negative control, en-AsCas12a (R1226A): en-AsCas12a nickase, en-AsCas12a (WT): wild-type en-AsCas12a. In Figures 4 of the main text, each corresponding

indel pattern was analyzed and the corresponding figure number was displayed at the bottom of the pie-chart.

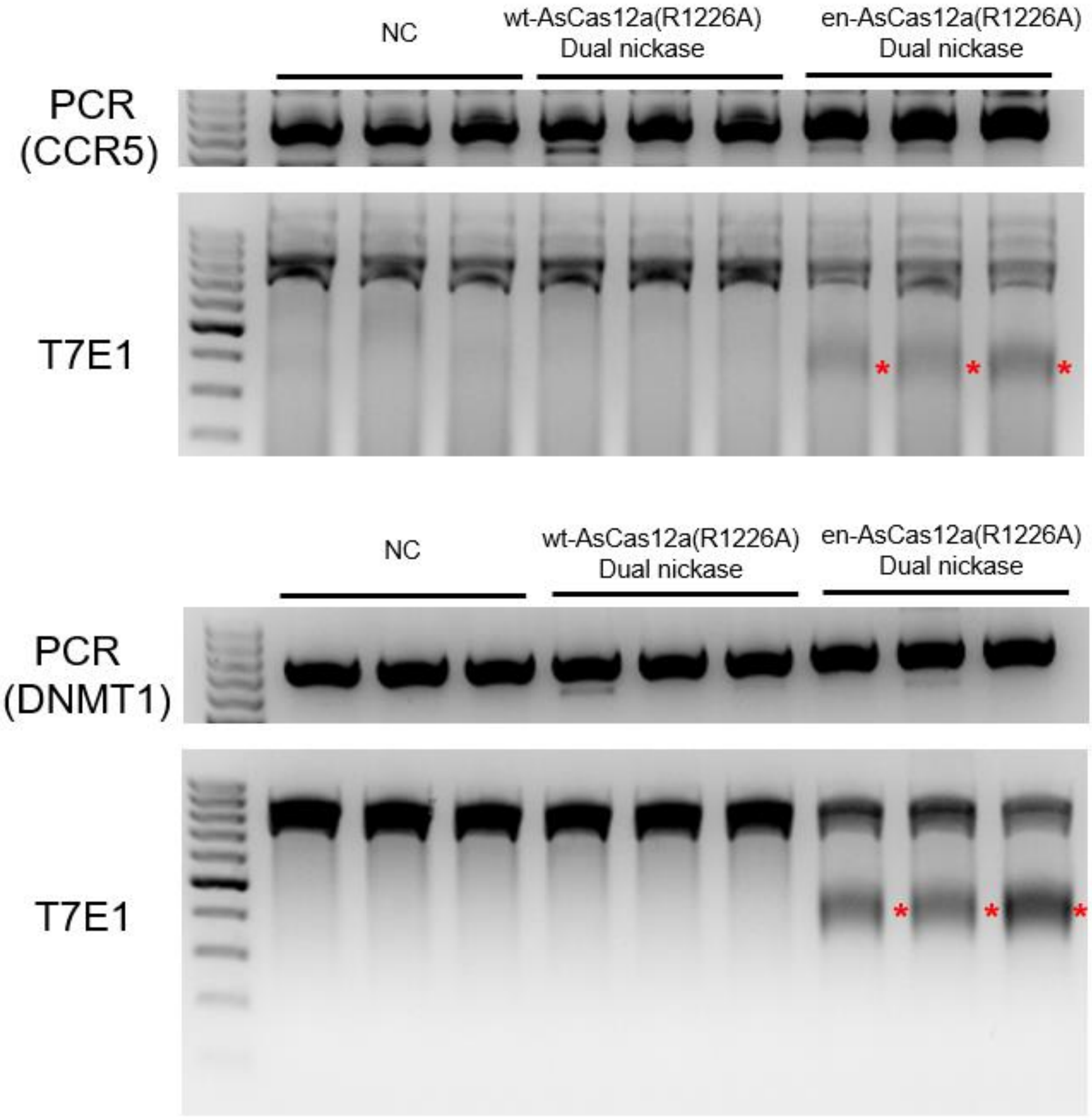

Supplementary Figure 8. Comparative analysis of genome editing efficiency of wt-AsCas12a (R1226A) and en-AsCas12a (R1226A) for endogenous sites in HeLa cell line. All results were analyzed by T7E1 cleavage assay, and red asterisks indicate cleaved DNA fragments.

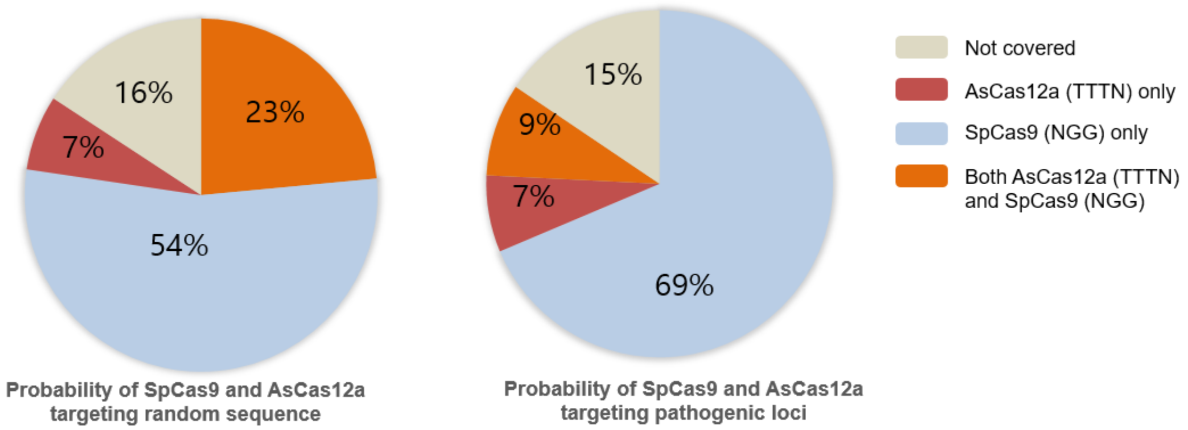

Supplementary Figure 9. Comparing gene targeting coverage of Cas12a and Cas9 using *in-silico*-based assay. In a pie-chart representing the target coverage of a CRISPR-Cas effector calculated *in-silico*, the left shows the range of SpCas9 and AsCas12a that can be covered when targeting a random sequence. The right shows the range of SpCas9 and AsCas12a that can be covered when targeting pathogenic loci in the Clinvar database (https://www.ncbi.nlm.nih.gov/clinvar/). In each pie-chart, a specific color represents the area covered by the Cas12a or Cas9 effector on the target DNA. Gray: Percentage (%) not recognized as both Cas12a and Cas9 effectors, Magenta: Percentage (%) recognized as Cas12a effector only, Light blue: Percentage (%) recognized as Cas9 effector only, Orange: Percentage (%) recognized as either Cas12a or Cas9 effector.

Main text Figure 1c

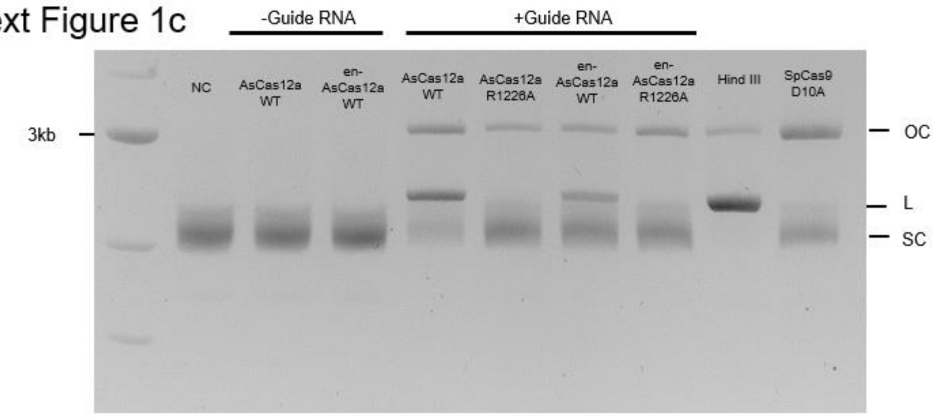

Main text Figure 1f

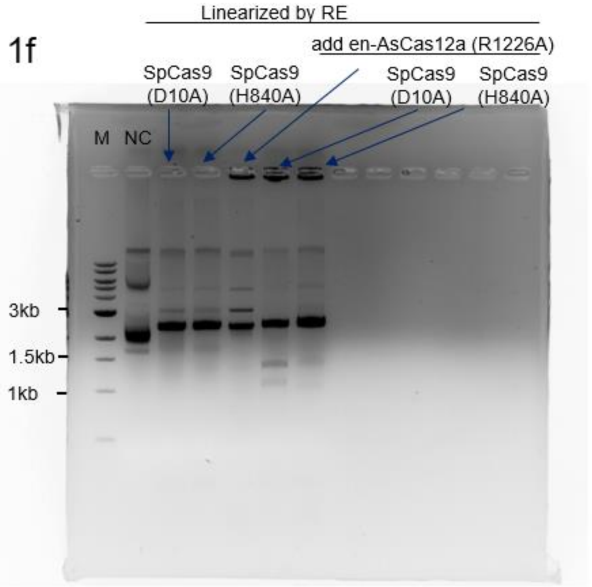

Supplementary Figure 1b

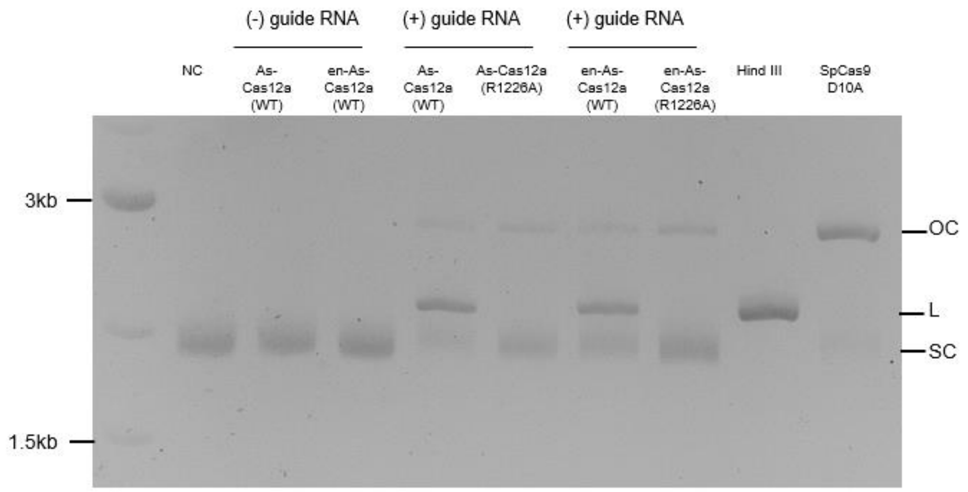

Supplementary Figure 8.

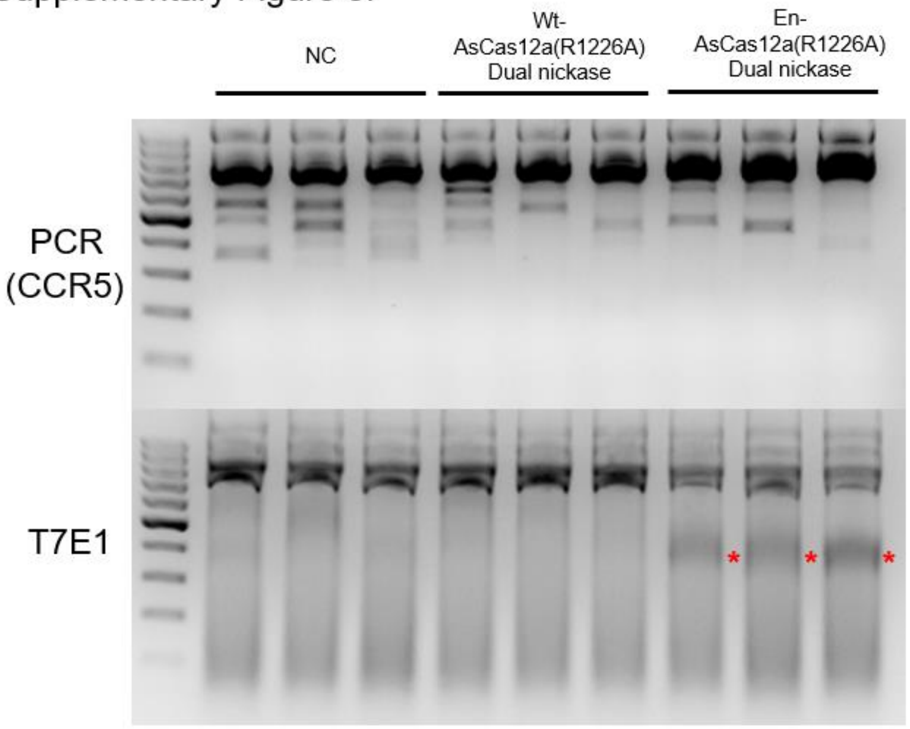

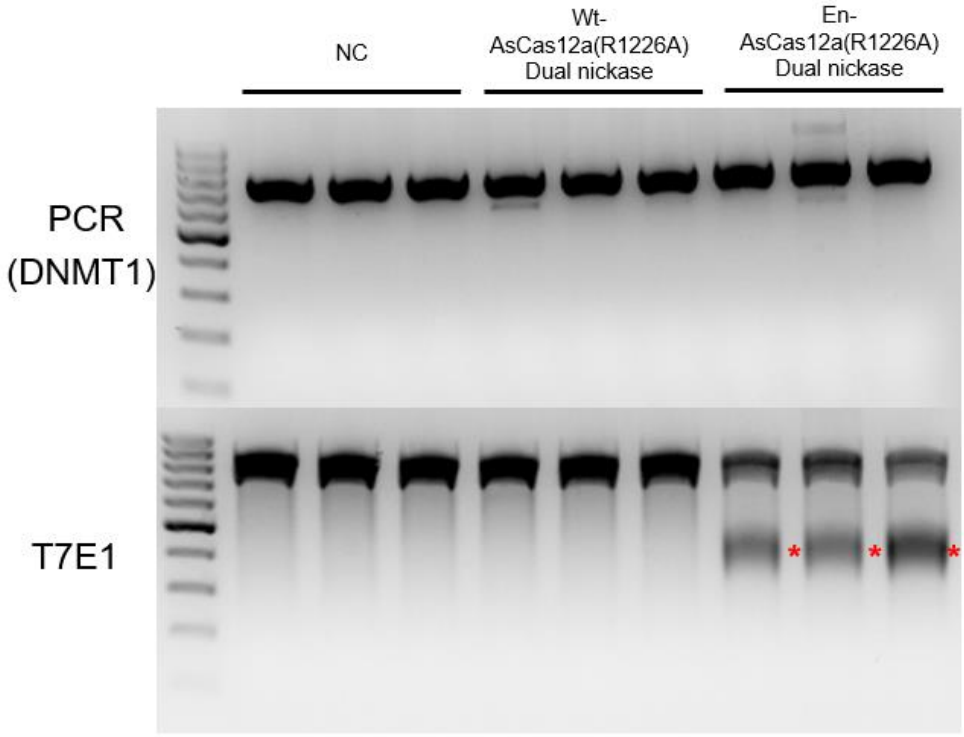

Supplementary Figure 10. Full image of gel photo data used in the main text and supplementary information. Images of the gels used (Main Figure 1c, 1f, Supplementary Figure 1b, Supplementary Figure 8) are listed in order.
